# Supplementary material for: Rates of obstetric intervention and associated perinatal mortality and morbidity among low-risk women giving birth in private and public hospitals in NSW (2000–2008): a linked data population-based cohort study
Source: BMJ Open. 2014 May 21;4(5):e004551. doi: 10.1136/bmjopen-2013-004551 (PMC4039844; doi:10.1136/bmjopen-2013-004551)
Supplement: Author's manuscript [file bmjopen-2013-004551.draft_revisions.pdf]

Rates of obstetric intervention and associated perinatal mortality and morbidity amongst low risk women giving birth in private and public hospitals in NSW (2000-2008): A linked data population based cohort study.

For peer review only

Journal:	*BMJ Open*
Manuscript ID:	bmjopen-2013-004551
Article Type:	Research
Date Submitted by the Author:	25-Nov-2013
Complete List of Authors:	Dahlen, Hannah; University of Western Sydney, School of Nursing and Midwifery<br>Tracy, Sally; University of Sydney, Nursing and Midwifery<br>Tracy, Mark; University of Sydney, Medicine<br>Bisits, Andrew; Royal Hospital for Women, Royal Hopsital for Women<br>Brown, Chris; University of Sydney, NHMRC Clinical Trials Centre<br>Thornton, Charlene; University of Western Sydney, School of Nursing and Midwifery
**Primary Subject Heading**:	Obstetrics and gynaecology
Secondary Subject Heading:	Epidemiology, Health policy, Paediatrics
Keywords:	Health policy < HEALTH SERVICES ADMINISTRATION & MANAGEMENT, NEONATOLOGY, OBSTETRICS

SCHOLARONE™
Manuscripts

Rates of obstetric intervention and associated perinatal mortality and morbidity amongst low risk women giving birth in private and public hospitals in NSW (2000-2008): A linked data population based cohort study.

Hannah G. Dahlen RN, RM, BN (Hons), MCommN, PhD, FACM

Professor of Midwifery

School of Nursing and Midwifery

Family and Community Health Group

Locked Bag 1797

Penrith South DC

NSW 2751

Australia

Ph: +61 2 96859118

Fax:+61 2 96859599

h.dahlen@uws.edu.au

Professor Sally Tracy

Professor Midwifery

Professor of Midwifery

University of Sydney,

Royal Hospital for Women, Sydney

For peer review only

For peer review only - http://bmjopen.bmj.com/site/about/guidelines.xhtml

Tel: 0420277106

Dr Mark Tracy PhD MSc (Epi Lond) FRACP(Paeds) MBBS DLSTHM

Senior Newborn Intensive Care Specialist and HOD

CNBC Westmead Hospital

Senior Clinical Lecturer Paediatrics and Child Health

University of Sydney

Tel (02) 9845 6568

Conjoint Professor Andrew Bisits MB bs(UNSW)FRANZCOG ,MMed Stat ,Dip Clin Epi

Acting Medical Co Director of Maternity, Lead Clinician Birthing Suite

Royal Hospital for Women

Randwick NSW 2033

0419418492

Mr Chris Brown, BSc, MBiostat

Biostatistician

NHMRC Clinical Trials Centre

For peer review only - http://bmjopen.bmj.com/site/about/guidelines.xhtml

For peer review only

Locked Bag 77

Camperdown NSW 1450

Dr Charlene Thornton BN, Grad.Dip.Mid, M Sc Med (Research), M Sc Med (Clin Epi), PhD

Post Doctoral Research Fellow

School of Nursing and Midwifery

Family and Community Health Group

Locked Bag 1797

Penrith South DC

NSW, Australia

Keywords: low risk, low risk, induction, vaginal birth, obstetric care, caesarean, birth trauma

Word count excluding title page, abstract, figures and tables: 3975

For peer review only - http://bmjopen.bmj.com/site/about/guidelines.xhtml

Abstract

Objectives: To examine the rates of obstetric intervention and associated perinatal mortality and morbidity in the first 28 days amongst low risk women giving birth in private and public hospitals in NSW (2000-2008) using linked data.

Design: Linked data population based cohort study involving five data sets.

Setting: New South Wales, Australia.

Participants: 691,738 women giving birth to a singleton baby during the period 2000 to 2008.

Main outcome measures: Rates of neonatal resuscitation, perinatal mortality, neonatal admission following birth and readmission to hospital in the first 28 days of life in public and private obstetric units.

Results: Among low risk women rates of obstetric intervention were significantly higher in private hospitals. Neonates born in private hospitals were more likely to be less than 40 weeks gestation compared to those born in public hospitals, more likely to have some form of resuscitation, less likely to have an Apgar <7 at five minutes and less likely to be transferred between hospitals. Neonates born in private hospitals to low risk mothers were more likely to have a morbidity attached to the birth admission and to be readmitted to hospital in the first 28 days for birth trauma; hypoxia; jaundice; feeding difficulties; sleep /behavioural issues; respiratory conditions and circumcision. Neonates born in a private hospital were less likely to be admitted for prophylactic antibiotics and for socioeconomic circumstances (eg. housing, distance, adoption, assumption of care). Rates of perinatal mortality were not statistically different between the two groups.

Conclusion:

For peer review only - http://bmjopen.bmj.com/site/about/guidelines.xhtml

High rates of obstetric intervention amongst low risk women in private hospitals appear to be associated with higher rates of morbidity seen in the neonate and no evidence of a reduction in perinatal mortality.

Keywords: low risk primip, low risk multip, induction, vaginal birth, obstetric care, caesarean, birth trauma

Article focus:

- To examine the rates of obstetric intervention and associated perinatal mortality and morbidity in the first 28 days amongst low risk women giving birth in private and public hospitals in NSW (2000-2008) using linked data.
- Rates of neonatal resuscitation, perinatal mortality, neonatal admission following birth and readmission to hospital in the first 28 days of life in public and private obstetric units.

Key messages

- Babies born to low risk mothers in private hospitals are more likely to require some resuscitation compared to those born in public hospitals. They are also more likely to have a morbidity coded on their birth admission and to be readmitted within 28 days following birth
- For babies born in private hospitals to low risk mothers there are higher rates of morbidity including birth trauma, hypoxia, jaundice, feeding difficulties, sleep/behavioural issues, respiratory conditions and circumcision. For babies born in public hospitals there are higher rates of antibiotic use and admission for socio-economic reasons (eg housing, distance, adoption and assumption of care)
- Rates of perinatal mortality were not statistically different between babies born to low risk women giving birth in private and public hospitals

Strengths and limitations of this study

- The strength of this study lies in the large sample size of birth data from over half a million women and babies and the >1.1 million admissions associated with these births.
- The use of data from five population based datasets which have been linked to enhance validity and ascertainment
- Limitations are the restricted number of variables that are included and the scarcity of specific information on potential confounders.
- Body mass index and key sociodemographic risk factors could not be controlled for and this would have added risk to women giving birth in public hospitals.

For peer review only - http://bmjopen.bmj.com/site/about/guidelines.xhtml

Introduction

In Australia, the national statistics reveal that 32.8% (n =70,332) of women giving birth in 2011 elected private status, with 29.9% of women giving birth in private hospitals directly under private obstetric care [1]. The remaining 3% of privately insured women received a combination of midwifery and medical care in public hospitals. The remaining (n=139, 486) 65% of women receive care as public patients in public hospitals in Australia. Women who are privately insured have been reported to have better maternal and perinatal outcomes compared to women who give birth in public hospitals as public patients [2]; but it has been argued that these women tend to be less socioeconomically disadvantaged and healthier [3] and therefore might be expected to have better outcomes. Arguments about the impact of private status on health outcomes are in reality complex.

What is not disputed are the much higher rates of obstetric intervention that occur in private hospitals in Australia. At a national level, the intervention rates in childbirth, such as caesarean section, are significantly higher in the private sector (43.1% vs 28.4%) and the rates of normal vaginal birth significantly lower (42.7% vs 61%) [1]. Despite the rising intervention rates over the past decade, the perinatal mortality rate overall has not shown a corresponding decline. There is also growing concern that the short and long term morbidity associated with major obstetric interventions, such as caesarean, may not be insignificant for the mother [4] and the baby [5, 6]. The cost to the tax payer of the rising intervention in childbirth is also significant [7, 8].

A recent study in New South Wales, Australia [9] found among 293,840 low-risk women, rates of obstetric intervention were highest in private hospitals and lowest in public hospitals. Low-risk primiparous women giving birth in a private hospital compared to a public hospital had higher rates of induction (31% vs 23%); instrumental birth (29% vs 18%); caesarean section (27% vs 18%), epidural (53% vs 32%), episiotomy (28% vs 12%) and lower normal vaginal birth rates (44% vs 64%). Low-risk multiparous women had higher rates of instrumental birth (7% vs 3%), caesarean section (27% vs 16%), epidural (35% vs 12%), episiotomy (8% vs 2%) and lower normal vaginal birth rates (66% vs 81%). Following a

For peer review only - http://bmjopen.bmj.com/site/about/guidelines.xhtml

For peer review only

comparison with data from the decade previously [10], these interventions were found to have increased by 5% for women in public hospitals and by over 10% for women in private hospitals. Among low-risk primiparous women giving birth in private hospitals, 15 per 100 women had a vaginal birth with no obstetric intervention compared to 35 per 100 women giving birth in a public hospital [9]. Concern was expressed that perinatal mortality and morbidity was not reported in that paper [11]. In this study we aim to examine the rates of obstetric intervention and associated perinatal mortality and morbidity attached to the birth admission and readmission to hospital in the first 28 days of life for low risk women giving birth in private and public hospitals in NSW (2000-2008).

Methods

Data Sources

The New South Wales Centre conducted linkage of several datasets via the Health Record Linkage (CHeReL). Pregnancy and birth data for the time period July 1st 2000 till June 2nd 2008 of all singleton births were provided by New South Wales (NSW), Ministry of Health as recorded in the NSW Midwives Data Collection (MDC), now the Perinatal Data Collection (PDC). This population based surveillance system contains maternal and infant data on all births of greater than 400 grams birth weight and/or 20 weeks gestation. The NSW PDC contains statistics on one third of all births which occur in Australia annually and provided data on maternal age, maternal hypertension, maternal diabetes, parity, private/public patient status, fetal presentation, onset of labour, gestation at birth, delivery type, Apgar scores and admission to neonatal intensive care and resuscitation details for the neonate. This dataset (NSW PDC was linked to the Admitted Patient Data Collection (APDC) for the time period July 1st 2000 – June 30th 2008. The APDC records all admitted patient services provided by NSW Public Hospitals, Public Psychiatric Hospitals, Public Multi-Purpose Services, Private Hospitals, and Private Day Procedures Centres. The APDC provided additional information, such as data on maternal medical conditions, which was used to exclude further maternal cases and was used to calculate admission and readmission details for neonates. Further linkage occurred to the NSW Registry of Births, Deaths and Marriages (RBDM) and

For peer review only - http://bmjopen.bmj.com/site/about/guidelines.xhtml

the Australian Bureau of Statistics Death Data, which provided mortality data. The NSW Register of congenital conditions provided cases of congenital conditions, as did the coding in the APDC. Any neonate (and mother pair) with a recorded congenital condition (ICD-10-AM codes Q0.0-Q99.9) on either dataset was removed from the dataset due to their high-risk status. Probabilistic data linkage techniques were utilised for data linkage and de-identified datasets were provided for analysis. Probabilistic record linkage software assigns a 'linkage weight' to pairs of records. For example, records that match perfectly or nearly perfectly on first name, surname, date of birth and address have a high linkage weight, and records that match only on date of birth have a low linkage weight. If the linkage weight is high it is likely that the records truly match, and if the linkage weight is low it is likely that the records are not truly a match. This technique has been shown to have a false positive rate of 0.3% of records [12].

Gestation is recorded at birth and is also recorded in the database according to the woman's menstrual history, usually combined with a routine scan at 12-13 weeks.

Admission to neonatal intensive care refers to admission to special care nursery (SCN) or neonatal intensive care unit (NICU).

Any resuscitation includes suction of the mouth or nostrils at birth; oxygen administered by mask; intermittent positive pressure respiration (IPPR) by bag and mask or by intubation; external cardiac massage and ventilation.

Ethical approval was obtained from the NSW Population and Health Services Research Ethics Committee, Protocol No.2010/12/291.

Subjects

We classified the low risk Primipara as a primiparous woman aged 20-34 years, who had no pre-existing or pregnancy related hypertension or diabetes, was a non-smoker, and gave birth at 37-41 weeks gestation to a baby in a cephalic presentation within the 10th and 90th centiles for birth weight. The low risk multipara was a multiparous woman aged 20-34 years, who had no pre-existing or pregnancy related hypertension

For peer review only - http://bmjopen.bmj.com/site/about/guidelines.xhtml

or diabetes, was a non-smoker, gave birth at 37-41 weeks gestation to a fetus in a cephalic presentation within the 10th and 90th centiles for birth weight. We excluded women with a previous caesarean section or who were induced for a medical indication, or who underwent a caesarean section for a medical indication or gave birth without a trained birth attendant (born before arrival). These characteristics were defined firstly from the PDC with additional medical conditions identified in the APDC being used to exclude cases.

Outcomes

Any neonatal admission including the ICD-10-AM codes Z37.0 (single live birth), Z37.1 (single stillbirth) or Z38.0 (singleton born in hospital) was deemed the birth admission and any ICD-10-AM codes referring to conditions which arise in the perinatal period (P00-P96) and those referring to factors influencing health status and contact with health services (Z00-Z99) which were included in this admission were deemed morbidities associated with the birth admission. Any other admission following this discharge from the initial birth admission to home or another hospital was deemed a readmission and included transfers to a hospital other than that where the birth occurred. When examining readmission data, all ICD-10-AM codes recorded were reviewed and those where ≥10 events occurred was marked for analysis.

Stillbirth and neonatal deaths were calculated from multiple sources. A detected case on any of the following four datasets was deemed a death. The PDC 'Discharge status' variable or admissions in the APDC where the case mode separation was coded as 'Died' or the NSW RBDM or ABS Death Data where a death had been recorded. The maternal admission data for any admission that occurred during the pregnancy, as well as the birth admission for all cases of stillbirth or neonatal death were examined to determine any maternal medical or pregnancy related condition. Reasons for stillbirth and neonatal death were taken from the principal cause noted on the death registration. If the death was not yet registered, the principal diagnosis as recorded in the neonate's birth or subsequent admission was utilised. In any case where either of these two methods did not supply a reason for death or principal diagnosis, no reason for death was recorded. This methodology of utilising

For peer review only - http://bmjopen.bmj.com/site/about/guidelines.xhtml

multiple data sources to confirm a diagnosis has been shown by Lain et al (2012) to be the most reliable way to increase ascertainment of cases (13).

Obstetric intervention was defined to include induction, epidural use, episiotomy, instrumental delivery (requiring the use of forceps or vacuum) and delivery via caesarean section.

Data analysis

The cohort was divided into primiparous and multiparous women for the primary analysis of birth outcomes. When examining neonatal status at birth and mortality odds ratios were calculated using logistic regression with and without adjustment for age and gestation. For neonatal morbidity at birth and readmission, chi-square statistics were calculated for observed, non-mutually exclusive, events. Taking into account the size of the cohort and the number of analyses undertaken, results were considered significant at the level $p<0.01$. Analysis was undertaken with IBM SPSS v.20®

Results

Maternal characteristics, interventions and outcomes

The PDC dataset for the time period January 1st 2000 to 31st December 2008 contained the antenatal, birth and postnatal details on 691 738 births. The APDC for the same time period contained >1.1 million admissions for the neonates of these women.

From the total population of primiparous women (288,309 women), 29,597 low risk primiparous women gave birth in private hospitals in NSW and 79,792 low risk primiparous women gave birth in public hospitals. The rate of obstetric intervention was significantly higher amongst those who gave birth in private hospitals compared to those who gave birth in public hospitals when all interventions for pre-specified medical reasons were removed. Low risk primiparous women giving birth in private hospitals had higher rates of induction for no medical reason (19%

For peer review only - http://bmjopen.bmj.com/site/about/guidelines.xhtml

vs 7%), instrumental birth (30% vs 20%), caesarean section (25% vs 16%), epidural (71% vs 35%) and episiotomy (42% vs 23%). Severe perineal trauma was slightly lower in a private hospital in first time mothers (4.7% vs 5.4%) (Table 1).

Amongst the total population of multiparous women (403,429 women), 28,703 low risk multiparous women gave birth in private hospitals and 99,212 low risk multiparous women gave birth in public hospitals The rate of obstetric intervention was significantly higher amongst those who gave birth in private hospitals in NSW compared to those who gave birth in public hospitals when all interventions for specific medical reasons were removed. Low risk multiparous women who gave birth in private hospitals had higher rates of induction for no medical reason (32% vs 10%), instrumental birth (8% vs 3%), epidural (34% vs 10%) and episiotomy (16% vs 5.%) and similar rates of severe perineal trauma (0.9%). The caesarean section rate still remained higher in the private cohort (5.9% vs 4.5%) though this was mostly associated with elective caesarean section (Table 2).

Perinatal characteristics, interventions and outcomes

There was no difference in birth weight between babies born in a private and public hospital. Babies born in a private hospital were significantly more likely to be born at 37, 38, 39 and 40 weeks and less likely to be born at 41 weeks gestation (Figure 1.).

Babies of primiparous women born in a private hospital were less likely to have an Apgar of <7 at five minutes (AOR 1.34 95% CI 1.77-1.530; p<0.001) less likely to have no resuscitation (AOR 0.354 95% CI 0.354-0.374; p<0.001). Babies born to low risk primiparous women in a private hospital were no more likely to be admitted to special care and/or neonatal intensive care (AOR 1.03 95% CI 0.984-1.075; p 0.210) and were less likely to have their baby transferred to another hospital (AOR 7.55 95% CI 6.522-8.738; p<0.001). There was no difference in the perinatal mortality rate for babies of primiparous women born in private or public hospitals (AOR 1.49 95% CI 0.926-2.410; p 0.100) (Table 3). Similar outcomes were seen for babies born to multiparous women in private and public hospitals (Table 4).

For peer review only - http://bmjopen.bmj.com/site/about/guidelines.xhtml

Reason for birth admission of neonate

We examined neonatal morbidity as coded on the neonatal birth admission record and found fewer babies overall had a morbidity record in the public sector compared to the private sector (40% vs 53.9%). There were some significant differences noted under the main ICD-10-AM Grouping *Certain conditions originating in the perinatal period (P00-P96)*. Babies born in a private hospital were more likely to have been affected by a forceps or vacuum delivery and were more likely to have trauma to the scalp (8.1% vs 4.1%), intrauterine hypoxia (4.3% vs 2.2%), jaundice (11.7% vs 2.9%), minor cardiac murmurs (0.6% vs 0.3%), conjunctivitis (7.5% vs 2.3%), respiratory conditions (2.4% vs 1.1%), temperature regulation issues (5.2% vs 2.4%) feeding difficulties (9.6% vs 4.3%), carbohydrate metabolism issues (2.2% vs 1.0%), vomiting (1.4% vs 0.7%) and be circumcised immediately following birth (6.7% vs 0.2%). Babies born in a public hospital were more likely to be admitted for observation and evaluation (11.8% vs 9.4%) have prophylactic antibiotics (1.0% vs 0.4%) and be admitted for socioeconomic circumstances (eg. housing, distance, adoption, assumption of care) (1.1% vs 0.1%) (Table 5)

Reason for birth re-admission of neonate up to 28 days of age

We examined the reasons for readmission of babies up until 28 days of age and found, that though the numbers are small, more babies born in private hospitals were readmitted compared to babies born in a public hospital (0.95% vs 0.65% (Table 6). Babies born in private hospitals were more likely to be readmitted for infectious diseases (18.1% vs 14.4%), endocrine, nutritional and metabolic disorders (6.4% vs 3%), behavioural disorders (4.8% vs 2.5%), sleep disorders (2.6% vs 0.6%), hypoglycaemia (2% vs 0.9%), birth trauma such as cephalohaematoma (2.0% vs 0.9%); trauma involving the scalp (4.4% vs 2.1%) and excessive crying (12.1% vs 7.8%). Babies born in public hospitals were more likely to be readmitted with respiratory disorders (23.3% vs 8%), injury and poisoning (eg burns) (6.2% vs 2.2%), for observation (6.8% vs 4.9%), antibiotic therapy (3.3% vs 0.7%) and socioeconomic circumstances (housing, distance, adoption, assumption of care (13.5% vs 3.9%).

Combined birth and readmission neonatal morbidity for selected codes

For peer review only - http://bmjopen.bmj.com/site/about/guidelines.xhtml

When we combined major birth and readmission morbidities for key selected codes we found that in the first 28 days following birth, babies born in private hospitals were significantly more likely to be admitted for feeding difficulties (9.6% vs 4.4%), circumcision (6.7% vs 0.3%), birth trauma (mostly scalp trauma) (12.2% vs 6.6%), hypoxia (4.2% vs 2.2%), respiratory disorders (3% vs 1.4%) and seep/behavioural issues (0.5% vs 0.2%). Babies born in public hospitals were more likely to be admitted for socioeconomic circumstances such as housing, distance, adoption or assumption of care (1.2% vs 0.2%) and prophylactic antibiotics (1.1% vs 0.4%) (Table 7 & Figure 2).

Discussion

Intervention rates

Despite being an extremely low risk cohort, less than half the primiparous women in this study giving birth in a private hospital had a normal vaginal birth (45% vs 65%); this was 20% lower than in the public cohort. One in five primiparous women giving birth in a private hospital were induced and nearly one in two had an episiotomy. For low risk multiparous women giving birth in a private hospital nearly one in three were induced. The trend for higher intervention rates has been reported for low risk women giving birth in the private sector in Australia previously and continues to show an increase [9, 14, 15]. In a recent publication we showed that the rate of caesarean section had increased in both the private and public sector in the past decade in low risk women [9]. It has been argued in a previous publication that these high intervention rates in the private sector led to better perinatal outcomes than in the public sector [2]. This publication received significant criticism in letters to the editor [3, 16, 17] for several methodological flaws, including most significantly the failure to adjust for low birth weight, inadequate ascertainment of congenital abnormalities and failure to look at perinatal morbidity. In this study we included only low risk women, adjusting for maternal age and gestational age differences. We also removed all babies with congenital abnormalities from this data set. We found that the perinatal mortality rate was not statistically different when the populations were matched as much as is possible for maternal risk.

Neonatal resuscitation and admission to SCN/NICU

For peer review only - http://bmjopen.bmj.com/site/about/guidelines.xhtml

We found that babies born in a private hospital were much more likely to experience some form of resuscitation, in particular twice the rate of suctioning at birth. Routine suctioning for infants born with clear and/or meconium stained amniotic fluid is not recommended [18] as it can cause a bradycardia [19] and there is no evidence of benefit. We are unsure why such a high rate of newborn suctioning continues in the private sector. While rates of Apgar scores of ≤7 at five minutes were slightly higher amongst women who gave birth in public hospital, and this has been demonstrated in another recent Australian publication [15], overall the babies were no more likely to be admitted to SCN/NICU compared to babies born in private hospitals.

Neonatal admission and readmission

We found some interesting differences in morbidity however when examining morbidity attached to the birth admission and readmission to hospital in the first 28 days for codes that may be associated with the higher rates of obstetric intervention in the private sector and a different sociodemographic profile in the public sector.

Birth trauma, in particular injuries to the scalp, were twice as high in the private sector and these are generally associated with instrumental birth, including vacuum extraction [20-22]. With more women (nearly one in three primiparous women) experiencing an instrumental birth in the private cohort and one in five women in the public sector this is not surprising. Birth trauma is associated with a longer hospital stay and increased risk of admission to SCN/NICU as well as higher rates of neonatal morbidity including neurological morbidity (hypotonia, jitteriness, convulsions and hypoxic ischaemic encephalopathy) and jaundice [22].

Jaundice was observed to be higher in the private sector, which may be related to several factors, such as the elective delivery of babies at an earlier gestation, the increased scalp trauma due to the high instrumental birth rate, as discussed above, and potential breastfeeding difficulties due to higher use of epidural analgesia. Jaundice has been associated with birth trauma, in particular delivery by vacuum extraction, and problems with feeding, especially supplementary feeding [22, 23]. Earlier gestational age <39 weeks has also be found to be associated

For peer review only - http://bmjopen.bmj.com/site/about/guidelines.xhtml

with jaundice, with this decreasing with each week of additional gestation [24]. The gestational age of babies born in private hospitals in this study was significantly lower than in the public sector due to the high rates of non-medical induction of labour and non-medically indicated caesarean section before the onset of labour.

While there have been studies associating difficulties with breastfeeding and higher rates of jaundice, the recent publication from the Universal Screening for Hyperbilirubinemia Study Group found this was a minimal risk factor.

Twice as many babies who were born in a private hospital in this study were admitted or re-admitted with feeding problems compared to babies born in a public hospital. Feeding difficulties are associated with operative birth interventions and being early term [25, 26]. Breastfeeding outcomes are positively associated with uncomplicated unassisted vaginal birth where the mother and infant remain together and breastfeeding is started within an hour of the birth and following skin-to-skin contact. Interventions during labour and birth can impact on the initiation and duration of breastfeeding. Caesarean section [27-30], instrumental birth [31], epidural anaesthesia and opioid analgesia [32-34] use have all been associated with breastfeeding difficulties. All these birth interventions were higher in the private cohort in this study. In addition early term birth, which is mainly due to induction of labour and elective caesarean section (35, 36) is associated with increased breastfeeding difficulties along with other serious morbidities [37]. Unmedicated newborns are more highly aroused immediately following the birth [38] and able to breastfeed without assistance if given skin to skin contact and freedom from intrusive procedures [39, 40]. Following caesarean section there can be a significantly longer period of time until a mother touches and holds her newborn compared to an unassisted vaginal birth [41].

In a previous paper [6] using national Australian population data we found that among low-risk women who had an unassisted vaginal birth with spontaneous onset of labour and no labour augmentation, the odds of admission to neonatal intensive care or special care nursery were significantly increased when the baby was 37 weeks gestation at the time of birth compared to later gestations. Some claim that during the

For peer review only - http://bmjopen.bmj.com/site/about/guidelines.xhtml

final weeks of gestation the fetal brain goes through a marked increase in mass and nerve growth (corticoneurogenesis) which may be best left undisturbed [42]. In this study low risk women giving birth in private hospitals in NSW were much more likely to give birth at earlier gestations than their public hospital counterparts for every week up to and including 40 weeks, but they were significantly less likely to deliver at 41 weeks. This may also help to explain why more babies born in a private hospital were readmitted with, respiratory, feeding, jaundice and sleep and behavioural problems.

Circumcision

Babies born in a private hospital were significantly more likely to be circumcised in the first 28 days of life (around 23 times more likely). This may be due to different information being given in private hospitals about the procedure or easy access to providers who perform the procedure. Circumcision rates are estimated to be between 10-20% in Australia [43] and are decreasing. A recent position statement of the Royal Australian College of Physicians states “that the frequency of diseases modifiable by circumcision, the level of protection offered by circumcision and the complication rates of circumcision do not warrant routine infant circumcision in Australia and New Zealand” [43].

Socioeconomic circumstances

The difference in the socioecononomic status of the women giving birth in public compared to private hospitals appears is demonstrated by the significantly higher rates of public hospital babies with a morbidity attached to the birth admission or readmission in the first 28 days for socioeconomic circumstances, including housing, distance, adoption and assumption of care. This again confirms what is already known that the two populations are very different sociodemographically with greater disadvantage in the public sector.

Limitations

For peer review only - http://bmjopen.bmj.com/site/about/guidelines.xhtml

Our study is limited to providing a snapshot of perinatal outcomes in one state in Australia in a defined time period for women who have no indicated risk at birth. However, this study provides useful data following on from our previous paper looking at obstetric intervention in private and public hospitals in NSW providing the reader with a detailed picture of perinatal mortality and morbidity. The advantages of using population-based datasets such as the PDC and the linkage to four other population-based databases include the size of the sample and the high level of accuracy of a validated dataset. The limitations are the restricted number of variables that are included and the scarcity of specific information on potential influencing variables. Previous validation studies have reported high levels of data accuracy for the majority of diagnoses and procedures conducted during labour and delivery in the state-wide data base [44, 45], although the recording of medical conditions and smoking are overall generally underreported [44, 46]. Having a linked data set provides a much richer picture than we have had previously of the morbidity and mortality associated with birth interventions. While we could not control for obesity due to lack of data, women who have private health insurance have lower rates of obesity and higher socioeconomic status, hence these health disadvantages are most likely over represented in the public women [47]. There are also several other socio-demographic factors we could not control for, such as education and income, that increase risk for the women giving birth in public hospitals. This study can only provide an overview of possible associations between obstetric interventions and neonatal outcomes and does not imply causality, which could be better obtained from prospective cohort studies.

Conclusion

The continual rise in obstetric intervention for low risk women in Australia may be contributing to increased morbidity for women and babies and higher cost of health care. The fact that these procedures which were initially life-saving are now so commonplace and do not appear to be associated with improved rates of perinatal mortality or morbidity demands close review. Early term delivery and instrumental births may be associated with increased morbidity in neonates and this requires urgent attention.

For peer review only - http://bmjopen.bmj.com/site/about/guidelines.xhtml

References

1 Li Z, Zeki R, Hilder L, Sullivan EA. Australia's mothers and babies 2010. Perinatal statistics series no. 27. Cat. no. PER 57. In: Unit ANPEaS, editor. Canberra2012.
2 Robson SJ, Laws P, Sullivan EA. Adverse outcomes of labour in public and private hospitals in Australia: a population based descriptive study. The Medical Journal of Australia 2009;190(9):474-7.
3 Tracy S, Welsh A, Dahlen H, Tracy M. Letter to the Editor re Robson SJ, Laws P, Sullivan EA. Adverse outcomes of labour in public and private hospitals in Australia: a population-based descriptive study. Med J Aust 2009; 190: 474-477. Medical Journal of Australia. 2009;191(10):579-80.
4 Clark E.A.S, Silver RM. Long-term maternal morbidity associated with repeat caesarean delivery. American Journal of Obstetrics and Gynecology. 2011;S2(December).
5 Hyde MJ, Mostyn A, Modi N, Kemp PR. The health implications of birth by caesarean section. Biological Reviews. 2012;87(1):229-43.
6 Tracy S, Tracy M, Sullivan E. Admission of Term Infants to Neonatal Intensive Care: A Population-Based Study. Birth 2007;34(4):301-7.
7 Tracy S, K , Tracy M. Costing the cascade: estimating the cost of increased obstetric intervention in childbirth using population data. British Journal of Obstetrics and Gynaecology. 2003;110:717-24.
8 Allen VM, O'Connell CM, Farrell SA, Baskett TF. Economic implications of method of delivery. Am J Obstet Gynecol 2005;193(1):192-7.
9 Dahlen HG, Tracy S, Tracy M, Bisits A, Brown C, Thornton C. Rates of obstetric intervention among low-risk women giving birth in private and public hospitals in NSW: a population-based descriptive study. BMJ Open. 2012;2:e001723 doi:10.1136/bmjopen-2012-001723.
10 Roberts C, L,, Tracy S, Peat B. Rates of obstetric intervention among private and public patients in Australia: population based descriptive study. British Medical Journal. 2000;312:137-41.
11 Buist R. Letter to the Editor: Private Obstetrics-again. BMJ Open. 2012;2(5):e001723 doi:10.1136/bmjopen-2012-.
12 CHeReL. Centre for Health Record Linkage (CHeReL). Quality Assurance Report 2012. http://wwwcherelorgau/media/24160/qa_report_2012pdf. 2012.
13 Lain SJ, Hadfield RM, Raynes-Greenow CH, Ford JB, Mealing NM, Algert CS, et al. Quality of data in perinatal population health databases: a systematic review. Med Care. 2012;50(4):e7-e20.
14 Roberts CL, Tracy S, Peat B. Rates for obstetric intervention among private and public patients in Australia: population based descriptive study. British Medical Journal. 2000 2000;321(7254):137-41.
15 Einarsdóttir K, Stock S, Haggar F, Hammond G, Langridge AT, Preen DB, et al. Neonatal complications in public and private patients: a retrospective cohort study. BMJ Open. 2013;Downloaded from bmjopen.bmj.com on November 11, 2013.
16 Evans N, Malcolm G, Gordon A. Letter to the Editor re Adverse outcomes of labour in public and private hospitals in Australia. MJA. 2009;191(10):579.
17 Chambers D. Letter to the Editor re Adverse outcomes of labour in public and private hospitals in Australia. MJA. 2009;191(10):578-9.
18 Wyllie J. Recent changes to UK newborn resuscitation guidelines. Arch Dis Child Fetal Neonatal Ed. 2012;97(1):F4-F7.
19 Kannapiran R, MKennea N. Resuscitation of the newborn. Obstetrics, Gynecology & Reproductive Medicine. 2012;22(4):92-7.
20 Doumouchtsis SK, Arulkumaran S. Head injuries after instrumental vaginal deliveries. Current Opion in Obstetrics and Gynaecology. 2006;18:129-34.

For peer review only - http://bmjopen.bmj.com/site/about/guidelines.xhtml

21 Towner D, Castro MA, Wilkens EE, Gilbert WM. Effect of Mode of Delivery in Nulliparous Women on Neonatal Intracranial Injury. The New England Journal of Medicine 1999;341:1709-14.
22 Linder N, Linder I, Fridman E, Kouadio F, Lubin D, Merlob P, et al. Birth trauma – risk factors and short-term neonatal outcome. The journal of Maternal-fetal & Neonatal Medicine. 2013;DOI: 10.3109/14767058.2013.789850.
23 Bertini G, Dani C, Tronchin M, Rubaltelli FF. Is breastfeeding really favouring early neonatal jaundice? Pediatrics. 2006;107(3):e41.
24 Bhutani VK, Stark AR, Lazzeroni LC, Poland R, Gourley GR, Kazmierczak S, et al. Predischarge Screening for Severe Neonatal Hyperbilirubinemia Identifies Infants Who Need Phototherapy. The Journal of Pediatrics. 2013;162(3):477-82.
25 Young PC, Korgenski K, Buchi KF. Early Readmission of Newborns in a Large Health Care System. Pediatrics. 2013;131(5):e1538-e44.
26 Brown A, Jordan S. Impact of birth complications on breastfeeding duration: an internet survey. Journal of Advanced Nursing. 2012;69(4):828-39.
27 Chapman D.J, Perez-Escamilla R. Identification of risk factors for delayed onset oflactation. Journal of the American Dietetic Association. 2003;99:450-4.

28 Dewey K.G., Nommsen-Rivers L.A., Heinig M.J, Cohen R.J. Risk factors for suboptimal infant breastfeeding behavior, delayed onset of lactation and excess neonatal weight loss. Pediatrics 2003;112(3):607-19.

29 Scott J.A., Binns C.W, Oddy W.H. Predictors of delayed onset of lactation. Maternal and Child Nutrition. 2007;3(3):186-93.

30 Jordan S., Emery S., Watkins A., Evans J.D., Storey M, Morgan G. Associations of drugs routinely given in labour with breastfeeding at 48 hours: analysis of the Cardiff Births Survey. British Journal of Gynecology. 2009;116(12):1622-9.
31 Leung G.M, Lam T.H, Ho L.M. Breast-feeding and its relation to smoking and mode of delivery. Obstetrics and Gynecology 2002;99(5):785-94.
32 Jordan S. Infant feeding and analgesia in labour: the evidence is accumulating. International Breastfeeding Journal Research. 2006;1(25):doi: 10.1186/746-4358-1-25.
33 Henderson J.J., Dickinson J.E., Evans S.F., McDonald S.J, M.J. P. Impact of intrapartum epidural analgesia on breast- feeding duration. Australian and New Zealand Journal of Obstetrics and Gynaecology. 2003;43(5):372–7.
34 Beilin Y., Bodian C., Weiser J., Sabera H., Ittamar A., Feierman D., et al. Effect of labor analgesia with and without fentanyl on infant breastfeeding: a prospective, randomized double-blind study. Anesthesiology. 2005;103(6):1211– 7.
35 Ohnsorg T, Schiff J. Preventing elective induction before 39 weeks. , 93(11), 44-46. Minnesota Medicine. 2010;93(11):44-6.
36 Oshiro BT, Henry E, Wilson J, Branch DW, Varner MW. Decreasing elective deliveries before 39 weeks of gestation in an integrated health care system. Obstetrics & Gynecology. 2009;113:804-11.
37 Reddy UM, Ko CW, Willinger M. “Early term” births (37-38 weeks) are associated with increased mortality. American Journal of Obstetrics & Gynecology. 2006;195(S202).
38 McLaughlin FJ, O’Connor S, Deni R. Infant state and behavior during the first postpartum hour. The Psychological Record 1981;31:455–8.
39 Widstrom A-M, Ransjo-Arvidson AB, Christensson K, Matthiesen A- S, Winberg J, Uvnas-Moberg K. Gastric suction in healthy newborn infants. Effects on circulation and developing feeding behaviour. Acta Paediatr. 1987;76:566–72.
40 Righard L. How do newborns find their mother’s breast? . Birth. 1995;22:174–5.

For peer review only - http://bmjopen.bmj.com/site/about/guidelines.xhtml

41 Fisher J, Astbury J, Smith A. Adverse psychological impact of opera- tive obstetric interventions: a prospective study. Aust NZ J Psychiatry 1997;31:728–38.
42 Adams-Chapman I. Insults to the developing brain and impact on neurode- velopmental outcome. Journal of Communication Disorders. Journal of Communication Disorders. 2009;42:256-62.
43 The Royal Australian College of Physicians. Circumcision of infant males. In: Physicians TRACo, editor. Sydney: The Royal Australasian College of Physicians; 2010.
44 Taylor L, Travis S, Pym M, Olive E, Henderson-Smart D. How useful are hospital morbidity data for monitoring conditions occurring in the perinatal period? Australian & New Zealand Journal of Obstetrics & Gynaecology. 2005;45:36 –41.
45 Roberts C, Bell J, Ford J, Morris J. Monitoring the quality of maternity care: how well are labour and delivery events reported in population health data? . Paediatirc and Perinatal Epidemiology. 2008;23:144-52.
46 Thornton C, Makris A, Ogle R, Hennessy A. Generic obstetric database systems are unreliable for reporting the Hypertensive Disorders of Pregnancy. Australian and New Zealand Journal Obstetrics and Gynaecology. 2004;44(505-509).
47 Finkelstein A, Fiebelkorn IC, Wang G. National medical Spendig attributable to overweight and obesity: How much and who's paying. Health Affairs, 2003 Project HOPE–The People-to-People Health Foundation, Inc., 219-226. 2003.

For peer review only - http://bmjopen.bmj.com/site/about/guidelines.xhtml

Table 1. Maternal characteristics, interventions and outcomes for low risk primiparous women in NSW (2000-2008)

**Low risk Primiparous women**	**Private Hospital (n=29597)**	**Public Hospital (n=79792)**
**Maternal age (years)**		
20-24	6.2%	28.9%
25-29	39.9%	40.9%
30-34	53.9%	30.2%
Weeks gestation at delivery		
37	4.5%	4.3%
38	15.4%	11.8%
39	27.8%	25.0%
40	43.2%	39.7%
41	9.1%	19.2%
**Type of labour**		
Spontaneous	71.9%	89.9%
Induced	19.2%	7.1%
No labour	8.9%	3.0%
**Delivery**		
Normal vaginal	44.9%	64.8%
Forceps	11.5%	6.7%
Vacuum	18.9%	12.9%
Total caesarean section	24.7%	15.6%
Caesarean section (after labour)	15.9%	12.6%
Caesarean section before the onset of labour	8.8%	3.0%
**Epidural**	70.8%	35.4%
**Episiotomy**	42.4%	23.3%

For peer review only

For peer review only - http://bmjopen.bmj.com/site/about/guidelines.xhtml

**Severe perineal trauma**	4.7%	5.4%

Table 2. Maternal characteristics, interventions and outcomes for low risk multiparous women in NSW (2000-2008)

Low risk Multiparous women	Private Hospital (n=28703)	Public Hospital (n=99212)
**Maternal age (years)**		
20-24	2.1%	16.8%
25-29	25.8%	38.8%
30-34	72.1%	44.4%
Weeks gestation at delivery		
37	4.1%	4.0%
38	18.7%	13.0%
39	31.6%	26.9%
40	40.2%	40.8%
41	5.4%	15.3%
**Type of labour**		
Spontaneous	64.0%	87.4%
Induced	32.1%	10.1%
No labour	3.9%	2.5%
**Delivery**		
Normal vaginal	86.1%	92.7%
Forceps	1.9%	0.7%
Vacuum	6.1%	2.1%
Total caesarean section	5.9%	4.5%
Caesarean section after labour	2.0%	2.0%
Caesarean section before the onset of labour	3.9%	2.5%#
**Epidural**	34.4%	9.5%

For peer review only - http://bmjopen.bmj.com/site/about/guidelines.xhtml

**Episiotomy**	16.2%	5.1%
**Severe perineal trauma**	0.9%	0.9%

Figure 1. Comparison of gestation at delivery between all women stratified by hospital type

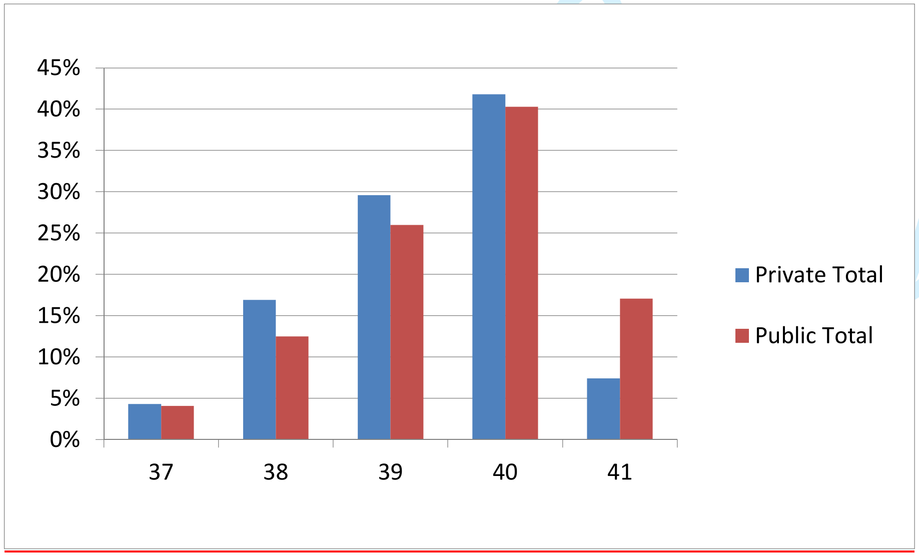

For peer review only - http://bmjopen.bmj.com/site/about/guidelines.xhtml

Table 3. Perinatal outcomes adjusted for maternal age and gestation at birth for primiparous women

	Private	Public			
	(n=29597)	(n=79791)	OR**	AOR**	p
Apgar<7 at 5 minutes	296 (1.0%)	1037 (1.3%)	1.36 (1.198-1.542)	1.34(1.177-1.530)	<0.001
Any resuscitation*	18498 (62.5%)	30560 (38.3%)	0.372 (0.363-0.382)	0.364 (0.354-0.374)	<0.001
Admitted to SCN and/or NICU	3078 (10.4%)	8139 (10.2%)	1.00 (0.964-1.049)	1.03 (0.984-1.075)	0.210
Transferred	178 (0.6%)	3351 (4.2%)	7.30 (6.289-8.403)	7.55 (6.522-8.738)	<0.001
Total Perinatal Mortality	22 (0.74/1000)	85 (1.06/1000)	1.40 (0.930-2.006)	1.49 (0.926-2.410)	0.100

*Any resuscitation includes: Suction, oxygen, IPPR by bag and mask, Intubation and IPPR, external cardiac massage and ventilation and other

** Private hospital is the reference category

For peer review only

Table 4. Perinatal outcomes adjusted for maternal age and gestation at birth for multiparous women

	Private	Public			
	(n=28703)	(n=99212)	OR**	AOR**	p
Apgar<7 at 5 minutes	67 (0.7%)	149 (0.5%)	1.314 (1.100-1.570)	1.389 (1.155-1.668)	<0.001
Any resuscitation*	14820 (51.6%)	29867 (30.1%)	0.404 (0.393-0.415)	0.399 (0.388-0.460)	<0.001
Admitted to SCN and/or NICU	1775 (6.2%)	5870 (5.9%)	0.957 (0.906-1.011)	1.027 (0.970-1.087)	0.363
Transferred	232 (0.8%)	4375 (4.4%)	5.661 (4.958-6.465)	6.516 (5.699-7.450)	<0.001
Total Perinatal Mortality	17 (0.59/1000)	76 (0.77/1000)	1.294 (0.765-2.189)	1.294 (0.750-2.232)	0.355

*Any resuscitation includes: Suction, oxygen, IPPR by bag and mask, Intubation and IPPR, external cardiac massage and ventilation and other

** Private hospital is the reference category

For peer review only

For peer review only - http://bmjopen.bmj.com/site/about/guidelines.xhtml

Table 5. Morbidity associated with birth admission coded on neonatal birth admission record

		Private		Public		p
**Total neonates with morbidity codes**		**23330**	**40.0%**	**96508**	**53.9%**	<0.001
**ICD-10-AM Grouping**						
**Certain conditions originating in the perinatal period (P00-P96)**						
	Fetus and newborn affected by maternal infectious and parasitic diseases	41	0.2%	989	1.0%	<0.001
	Fetus and newborn affected by forceps delivery	473	2.0%	1108	1.1%	<0.001
	Fetus and newborn affected by delivery by vacuum extractor	511	2.2%	1509	1.6%	<0.001
	**Birth trauma (all body systems)**	**2948**	**12.6%**	**6447**	**6.7%**	<0.001
	- specifically to scalp (included in above total)	1880	8.1%	3965	4.1%	<0.001
	Intrauterine hypoxia	993	4.3%	2170	2.2%	<0.001
	Other specified respiratory conditions of newborn	562	2.4%	1015	1.1%	<0.001
	Benign and innocent cardiac murmurs in newborn	139	0.6%	303	0.3%	<0.001
	Neonatal conjunctivitis specific to the perinatal period	1740	7.5%	2267	2.3%	<0.001
	**Jaundice related conditions**	**2728**	**11.7%**	**5166**	**2.9%**	<0.001
	Transitory disorders of carbohydrate metabolism specific to fetus and newborn	502	2.2%	942	1.0%	<0.001
	Temperature regulation	1214	5.2%	2275	2.4%	<0.001
	Vomiting in newborn	320	1.4%	693	0.7%	<0.001
	Feeding difficulties	2231	9.6%	4157	4.3%	<0.001
**factors influencing health status and contact with health services (Z00-Z99)**						
	Routine and ritual circumcision	1552	6.7%	187	0.2%	<0.001
	Observation and evaluation of newborn	2187	9.4%	11372	11.8%	<0.001
	Prophylactic chemotherapy (antibiotics)	93	0.4%	935	1.0%	<0.001
	Socioeconomic circumstances (housing, distance, adoption, assumption of care)	32	0.1%	1020	1.1%	<0.001
		3184	13.7%	49988	51.8%	

For peer review only - http://bmjopen.bmj.com/site/about/guidelines.xhtml

Table 6. Morbidity associated with readmission of the baby <28 days of age

		Private		Public		p
**Total admissions**		**689**		**1507**		
**Total neonates**		**552 (0.95%)**		**1155 (0.65%)**		<0.001
**ICD-10-AM Grouping**						
**Certain infectious and parasitic diseases**		**121**	**18.1%**	**217**	**14.4%**	<0.001
**Endocrine nutritional and metabolic diseases**		**30**	**4.4%**	**38**	**2.5%**	<0.001
	Volume depletion	18	2.6%	12	0.8%	<0.001
**Mental and behavioural disorders**		**33**	**4.8%**	**30**	**2.0%**	<0.001
	Non-organic hypersomnia	17	2.5%	19	1.3%	<0.001
**Diseases of the nervous system**		**34**	**4.9%**	**38**	**2.5%**	<0.001
	Disorders of the sleep wake schedule	18	2.6%	9	0.6%	<0.001
**Diseases of the ear and mastoid process**		**19**	**2.8%**	**23**	**1.5%**	<0.001
**Diseases of the respiratory system**		**155**	**8.0%**	**351**	**23.3%**	<0.001
	Acute obstructive laryngitis (croup)	6	0.9%	16	1.1%	=0.76
	Acute upper respiratory infection unspecified	16	2.3%	52	3.5%	0.84
	Pneumonia	6	0.9%	19	1.3%	=0.86
	Acute bronchiolitis	70	10.2%	175	11.6%	=0.17
	Unspecified acute lower respiratory tract infection	3	0.4%	12	0.8%	0.52
**Diseases of the digestive system**		**53**	**7.7%**	**92**	**6.1%**	<0.001
	Gastro-oesophageal reflux disease	26	3.8%	37	2.5%	0.01
**Certain conditions originating in the perinatal period**		**474**	**68.8%**	**1011**	**67.1%**	<0.001
	Cephalohaematoma due to birth trauma	14	2.0%	13	0.9%	<0.01
	Total birth trauma to scalp	30	4.4%	32	2.1%	<0.001

For peer review only - http://bmjopen.bmj.com/site/about/guidelines.xhtml

	Intrauterine hypoxia	4	0.6%	25	1.7%	0.43
	Other neonatal hypoglycaemia	14	2.0%	14	0.9%	<0.01
	Other transitory neonatal electrolyte and metabolic disturbances	5	0.7%	21	1.4%	=0.69
	Fever of newborn	5	0.7%	18	1.2%	0.92
	Feeding problems of newborn	40	5.8%	100	6.6%	=0.31
	**Neonatal jaundice**	**193**	**28%**	**90**	**6%**	<0.001
**Symptoms, signs and abnormal findings not elsewhere classified**		**198**	**28.7%**	**340**	**22.6%**	<0.001
	Fever	19	2.8%	27	1.8%	=0.02
	Feeding difficulties and mismanagement	25	3.6%	37	2.5%	<0.01
	Excessive crying	83	12.1%	117	7.8%	<0.001
**injury, poisoning and certain other consequences of external causes**		**15**	**2.2%**	**94**	**6.2%**	<0.001
	Burns	0	0.0%	30	2.0%	<0.001
**factors influencing health status and contact with health services**		**192**	**28.9%**	**691**	**45.9%**	<0.001
	Observation	34	4.9%	102	6.8%	=0.92
	Other prophylactic antibiotic therapy	5	0.7%	49	3.3%	<0.01
	Routine and ritual circumcision	68	9.9%	119	7.9%	<0.001
	Attention to surgical dressings and sutures	0	0.0%	28	1.9%	
	Socio-economic circumstances (housing, distance, adoption, assumption of care)	22	3.9%	156	13.5%	<0.001

For peer review only - http://bmjopen.bmj.com/site/about/guidelines.xhtml

Table 7. Combined birth and readmission neonatal morbidity for selected codes

	Private	Public	P*
Total feeding difficulties	2314 (9.6%)	4306 (4.4%)	<0.0001
Total circumcision	1620 (6.7%)	306 (0.3%)	<0.0001
Total socio-economic circumstances	54 (0.2%)	1176 (1.2%)	<0.0001
Total birth trauma	2922 (12.2%)	6492 (6.6%)	<0.0001
Total hypoxia	997 (4.2%)	2195 (2.2%)	<0.0001
Total jaundice	2818 (11.8%)	5359 (5.5%)	<0.0001
Total respiratory	717 (3.0%)	1366 (1.4%)	<0.0001
Total sleep/behavioural issues	118 (0.5%)	145 (0.2%)	<0.0001
Prophylactic antibiotics	98 (0.4%)	982 (1.1%)	<0.001

*chi-square

Figure 2. Birth trauma as a percentage of all births in Private and Public Hospitals

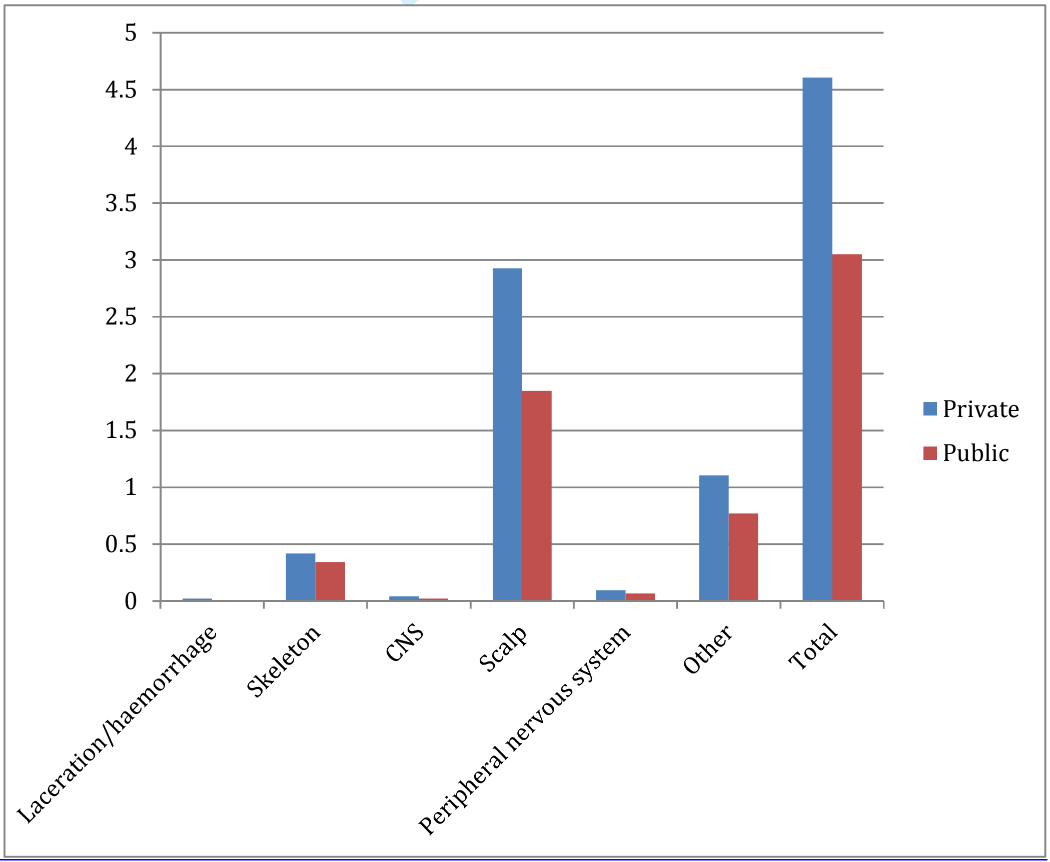

CNS-Central Nervous System, PNS- Peripheral Nervous System

Rates of obstetric intervention and associated perinatal mortality and morbidity amongst low risk women giving birth in private and public hospitals in NSW (2000-2008): A linked data population based cohort study.

Journal:	*BMJ Open*
Manuscript ID:	bmjopen-2013-004551.R1
Article Type:	Research
Date Submitted by the Author:	07-Feb-2014
Complete List of Authors:	Dahlen, Hannah; University of Western Sydney, School of Nursing and Midwifery<br>Tracy, Sally; University of Sydney, Nursing and Midwifery<br>Tracy, Mark; University of Sydney, Medicine<br>Bisits, Andrew; Royal Hospital for Women, Royal Hopsital for Women<br>Brown, Chris; University of Sydney, NHMRC Clinical Trials Centre<br>Thornton, Charlene; University of Western Sydney, School of Nursing and Midwifery
**Primary Subject Heading**:	Obstetrics and gynaecology
Secondary Subject Heading:	Epidemiology, Health policy, Paediatrics
Keywords:	Health policy < HEALTH SERVICES ADMINISTRATION & MANAGEMENT, NEONATOLOGY, OBSTETRICS

SCHOLARONE™
Manuscripts

For peer review only

Rates of obstetric intervention and associated perinatal mortality and morbidity amongst low risk women giving birth in private and public hospitals in NSW (2000-2008): A linked data population based cohort study.

Hannah G. Dahlen RN, RM, BN (Hons), MCommN, PhD, FACM

Professor of Midwifery

School of Nursing and Midwifery

Family and Community Health Group

Locked Bag 1797

Penrith South DC

NSW 2751

Australia

Ph: +61 2 96859118

Fax:+61 2 96859599

h.dahlen@uws.edu.au

Professor Sally Tracy

Professor Midwifery

Professor of Midwifery

University of Sydney,

Royal Hospital for Women, Sydney

For peer review only

For peer review only - http://bmjopen.bmj.com/site/about/guidelines.xhtml

Tel: 0420277106

Dr Mark Tracy PhD MSc (Epi Lond) FRACP(Paeds) MBBS DLSTHM

Senior Newborn Intensive Care Specialist and HOD

CNBC Westmead Hospital

Senior Clinical Lecturer Paediatrics and Child Health

University of Sydney

Tel (02) 9845 6568

Conjoint Professor Andrew Bisits MB bs(UNSW)FRANZCOG ,MMed Stat ,Dip Clin Epi

Acting Medical Co Director of Maternity, Lead Clinician Birthing Suite

Royal Hospital for Women

Randwick NSW 2033

0419418492

Mr Chris Brown, BSc, MBiostat

Biostatistician

NHMRC Clinical Trials Centre

For peer review only

Locked Bag 77

Camperdown NSW 1450

Dr Charlene Thornton BN, Grad.Dip.Mid, M Sc Med (Research), M Sc Med (Clin Epi), PhD

Post Doctoral Research Fellow

School of Nursing and Midwifery

Family and Community Health Group

Locked Bag 1797

Penrith South DC

NSW, Australia

Keywords: low risk, low risk, induction, vaginal birth, obstetric care, caesarean, birth trauma

Word count excluding title page, abstract, figures and tables: 4531

For peer review only - http://bmjopen.bmj.com/site/about/guidelines.xhtml

Rates of obstetric intervention and associated perinatal mortality and morbidity amongst low risk women giving birth in private and public hospitals in NSW (2000-2008): A linked data population based cohort study.

Abstract

Objectives: To examine the rates of obstetric intervention and associated perinatal mortality and morbidity in the first 28 days amongst low risk women giving birth in private and public hospitals in NSW (2000-2008) using linked data.

Design: Linked data population based cohort study involving five data sets.

Setting: New South Wales, Australia.

Participants: 691,738 women giving birth to a singleton baby during the period 2000 to 2008.

Main outcome measures: Rates of neonatal resuscitation, perinatal mortality, neonatal admission following birth and readmission to hospital in the first 28 days of life in public and private obstetric units.

Results: Rates of obstetric intervention amongst low risk women were significantly higher in private hospitals, with primiparous women 20% less likely to have a normal vaginal birth compared to the public sector. Neonates born in private hospitals were more likely to be less than 40 weeks gestation compared to those born in public hospitals and more likely to have some form of resuscitation, less likely to have an Apgar <7 at five minutes (if primiparous) and less likely to be transferred between hospitals. Neonates born in private hospitals to low risk mothers were more likely to have a morbidity attached to the birth admission and to be readmitted to hospital in the first 28 days for birth trauma; hypoxia; jaundice; feeding difficulties; sleep /behavioural issues; respiratory conditions and circumcision. Neonates born in a private hospital were less

For peer review only - http://bmjopen.bmj.com/site/about/guidelines.xhtml

likely to be admitted for prophylactic antibiotics and for socioeconomic circumstances (eg. housing, distance, adoption, assumption of care). Rates of perinatal mortality were not statistically different between the two groups.

Conclusion:

High rates of obstetric intervention amongst low risk women in private hospitals appear to be associated with higher rates of certain morbidity seen in the neonate and no evidence of a reduction in perinatal mortality.

Keywords: low risk primip, low risk multip, induction, vaginal birth, obstetric care, caesarean, birth trauma

Article focus:

- To examine the rates of obstetric intervention and associated perinatal mortality and morbidity in the first 28 days amongst low risk women giving birth in private and public hospitals in NSW (2000-2008) using linked data.
- Rates of neonatal resuscitation, perinatal mortality, neonatal admission following birth and readmission to hospital in the first 28 days of life in public and private obstetric units.

Key messages

- Babies born to low risk mothers in private hospitals are more likely to require some resuscitation compared to those born in public hospitals.
- For babies born in private hospitals to low risk mothers there are higher rates of morbidity such as birth trauma, hypoxia, jaundice, feeding difficulties, sleep/behavioural issues, respiratory conditions and circumcision. For babies born in public hospitals there are higher rates of antibiotic use and admission for socio-economic reasons (eg housing, distance, adoption and assumption of care)

For peer review only - http://bmjopen.bmj.com/site/about/guidelines.xhtml

For peer review only

- Rates of perinatal mortality were not statistically different between babies born to low risk women giving birth in private and public hospitals

Strengths and limitations of this study

- The strength of this study lies in the large sample size of birth data from over half a million women and babies and the >1.1 million admissions associated with these births.
- The use of data from five population based datasets which have been linked to enhance validity and ascertainment
- Limitations are the restricted number of variables that are included and the scarcity of specific information on potential confounders.
- Body mass index and key sociodemographic risk factors could not be controlled for and this would have added risk to women giving birth in public hospitals.

For peer review only - http://bmjopen.bmj.com/site/about/guidelines.xhtml

Introduction

In Australia, the national statistics reveal that 32.8% (n =70,332) of women giving birth in 2011 elected private status, with 29.9% of women giving birth in private hospitals directly under private obstetric care [1]. The remaining 3% of privately insured women received a combination of midwifery and medical care in public hospitals. The remaining (n=139, 486) 65% of women receive care as public patients in public hospitals in Australia. Women who are privately insured have been reported to have better maternal and perinatal outcomes compared to women who give birth in public hospitals as public patients [2]; but it has been argued that these women tend to be less socioeconomically disadvantaged and healthier [3] and therefore might be expected to have better outcomes. Arguments about the impact of private status on health outcomes are in reality complex.

What is not disputed are the much higher rates of obstetric intervention that occur in private hospitals in Australia. At a national level, the intervention rates in childbirth, such as caesarean section, are significantly higher in the private sector (43.1% vs 28.4%) and the rates of normal vaginal birth significantly lower (42.7% vs 61%) [1]. Despite the rising intervention rates over the past decade, the perinatal mortality rate overall has not shown a corresponding decline. There is also growing concern that the short and long term morbidity associated with major obstetric interventions, such as caesarean, may not be insignificant for the mother [4] and the baby [5, 6]. The cost to the tax payer of the rising intervention in childbirth is also significant [7, 8].

A recent study in New South Wales, Australia [9] found among 293,840 low-risk women, rates of obstetric intervention were highest in private hospitals and lowest in public hospitals. Low-risk primiparous women giving birth in a private hospital compared to a public hospital had higher rates of induction (31% vs 23%); instrumental birth (29% vs 18%); caesarean section (27% vs 18%), epidural (53% vs 32%), episiotomy (28% vs 12%) and lower normal vaginal birth rates (44% vs 64%). Low-risk multiparous women had higher rates of instrumental birth (7% vs 3%), caesarean section (27% vs 16%), epidural (35% vs 12%), episiotomy (8% vs 2%) and lower normal vaginal birth rates (66% vs 81%). Following a

For peer review only - http://bmjopen.bmj.com/site/about/guidelines.xhtml

comparison with data from the decade previously [10], these interventions were found to have increased by 5% for women in public hospitals and by over 10% for women in private hospitals [9]. Among low-risk primiparous women giving birth in private hospitals, 15 per 100 women had a vaginal birth with no obstetric intervention compared to 35 per 100 women giving birth in a public hospital [9]. Concern was expressed that perinatal mortality and morbidity was not reported in that paper [11]. In this study we aim to examine the rates of obstetric intervention and associated perinatal mortality and morbidity attached to the birth admission and readmission to hospital in the first 28 days of life for low risk women giving birth in private and public hospitals in NSW (2000-2008).

Methods

Data Sources

The New South Wales Centre for Health Record Linkage conducted linkage of several datasets via the Health Record Linkage (CHeReL). Pregnancy and birth data for the time period July 1st 2000 till June 2nd 2008 of all singleton births were provided by New South Wales (NSW), Ministry of Health as recorded in the NSW Midwives Data Collection (MDC), now the Perinatal Data Collection (PDC). This population based surveillance system contains maternal and infant data on all births of greater than 400 grams birth weight and/or 20 completed weeks gestation. Hospitals are coded either as private or public in the data set. However, the data identifying women who received care in public hospitals under private accommodation status is no longer collected as it had been in the years 1996-97 and for this reason patients who are under private obstetric care in public hospitals are not able to be differentiated from their public counterparts, so for this study we analysed the data by hospital (private/public). A previous study published in 2000 [10] showed that there was a moderating factor on intervention rates when women with private insurance status gave birth in a public hospital, leading to lower intervention rates than when they gave birth in private hospitals.

For peer review only - http://bmjopen.bmj.com/site/about/guidelines.xhtml

For peer review only

The NSW PDC contains statistics on one third of all births which occur in Australia annually and provided data on maternal age, maternal hypertension, maternal diabetes, parity, private/public patient status, fetal presentation, onset of labour, gestation at birth, delivery type, Apgar scores and admission to neonatal intensive care and resuscitation details for the neonate. This dataset (NSW PDC was linked to the Admitted Patient Data Collection (APDC) for the time period July 1st 2000 – June 30th 2008. The APDC records all admitted patient services provided by NSW Public Hospitals, Public Psychiatric Hospitals, Public Multi-Purpose Services, Private Hospitals, and Private Day Procedures Centres. The APDC provided additional information, such as data on maternal medical conditions, which was used to exclude further maternal cases and was used to calculate admission and readmission details for neonates. Further linkage occurred to the NSW Registry of Births, Deaths and Marriages (RBDM) and the Australian Bureau of Statistics Death Data, which provided mortality data. The NSW Register of congenital conditions provided cases of congenital conditions, as did the coding in the APDC. Any neonate (and mother pair) with a recorded congenital condition (ICD-10-AM codes Q0.0-Q99.9) on either dataset was removed from the dataset due to their high-risk status. Probabilistic data linkage techniques were utilised for data linkage and de-identified datasets were provided for analysis. Probabilistic record linkage software assigns a 'linkage weight' to pairs of records. For example, records that match perfectly or nearly perfectly on first name, surname, date of birth and address have a high linkage weight, and records that match only on date of birth have a low linkage weight. If the linkage weight is high it is likely that the records truly match, and if the linkage weight is low it is likely that the records are not truly a match. This technique has been shown to have a false positive rate of 0.3% of records [12].

Gestation is recorded at birth and is also recorded in the database according to the woman's menstrual history, usually combined with a routine scan at 12-13 weeks.

Admission to neonatal intensive care refers to admission to special care nursery (SCN) or neonatal intensive care unit (NICU).

For peer review only - http://bmjopen.bmj.com/site/about/guidelines.xhtml

Any resuscitation includes suction of the mouth or nostrils at birth; oxygen administered by mask; intermittent positive pressure respiration (IPPR) by bag and mask or by intubation; external cardiac massage and ventilation.

Ethical approval was obtained from the NSW Population and Health Services Research Ethics Committee, Protocol No.2010/12/291.

Subjects

We classified the low risk Primipara as a primiparous woman aged 20-34 years, who had no pre-existing or pregnancy related hypertension or diabetes, was a non-smoker, and gave birth at 37-41 completed weeks gestation to a singleton baby in a cephalic presentation within the 10th and 90th centiles for birth weight. The low risk multipara was a multiparous woman aged 20-34 years, who had no pre-existing or pregnancy related hypertension or diabetes, was a non-smoker, gave birth at 37-41 completed weeks gestation to a singleton baby in a cephalic presentation within the 10th and 90th centiles for birth weight. We excluded women with a previous caesarean section or who were induced for a medical indication, or who underwent a caesarean section for a pre-existing medical indication or gave birth without a trained birth attendant (born before arrival). If a caesarean section was undertaken during labour however for non-reassuring heart rate, dystocia etc these women were included in the study. These characteristics were defined firstly from the PDC with additional medical conditions identified in the APDC being used to exclude cases.

Outcomes

Any neonatal admission including the ICD-10-AM codes Z37.0 (single live birth), Z37.1 (single stillbirth) or Z38.0 (singleton born in hospital) was deemed the birth admission and any ICD-10-AM codes referring to conditions which arise in the perinatal period (P00-P96) and those referring to factors influencing health status and contact with health services (Z00-Z99) which were included in this admission were deemed morbidities

For peer review only - http://bmjopen.bmj.com/site/about/guidelines.xhtml

associated with the birth admission. Any other admission following this discharge from the initial birth admission to home or another hospital was deemed a readmission and included transfers to a hospital other than that where the birth occurred. When examining readmission data, all ICD-10-AM codes recorded were reviewed and those where ≥10 events occurred was marked for analysis. Events were grouped in body systems where appropriate or under headings such as infection for ease of analysis and interpretation.

Stillbirth and neonatal deaths were calculated from multiple sources but were limited to those that occurred 28 days from the birth and they were only counted once. A detected case on any of the following four datasets was deemed a death. The PDC 'Discharge status' variable or admissions in the APDC where the case mode separation was coded as 'Died' or the NSW RBDM or ABS Death Data where a death had been recorded. The maternal admission data for any admission that occurred during the pregnancy, as well as the birth admission for all cases of stillbirth or neonatal death were examined to determine any maternal medical or pregnancy related condition. Reasons for stillbirth and neonatal death were taken from the principal cause noted on the death registration. If the death was not yet registered, the principal diagnosis as recorded in the neonate's birth or subsequent admission was utilised. In any case where either of these two methods did not supply a reason for death or principal diagnosis, no reason for death was recorded. This methodology of utilising multiple data sources to identify cases has been shown by Lain et al (2012) to be the most reliable way to increase ascertainment of cases (13).

Obstetric intervention was defined to include induction, epidural use, episiotomy, instrumental delivery (requiring the use of forceps or vacuum) and delivery via caesarean section.

Data analysis

The cohort was divided into primiparous and multiparous women for the primary analysis of birth outcomes. When examining neonatal status at birth and mortality odds ratios were calculated using logistic regression with and without adjustment for age and gestation. For neonatal

For peer review only - http://bmjopen.bmj.com/site/about/guidelines.xhtml

morbidity at birth and readmission, chi-square statistics were calculated for observed events. Taking into account the size of the cohort and the number of analyses undertaken, results were considered significant at the level $p<0.01$. Analysis was undertaken with IBM SPSS v.20®

Results

Maternal characteristics, interventions and outcomes

The PDC dataset for the time period July 1st 2000 to 2nd June 2008 contained the antenatal, birth and postnatal details on 691 738 births. The APDC for the time period July 1st 2000 to 30th June 2008 (28 days from birth) contained >1.1 million admissions for the neonates of these women.

From the total population of primiparous women (288,309 women), 29,597 low risk primiparous women gave birth in private hospitals in NSW and 79,792 low risk primiparous women gave birth in public hospitals. The rate of obstetric intervention were much higher amongst those who gave birth in private hospitals compared to those who gave birth in public hospitals when all interventions for pre-specified medical reasons were removed. Low risk primiparous women giving birth in private hospitals had higher rates of induction for no medical reason (19% vs 7%), instrumental birth (30% vs 20%), caesarean section (25% vs 16%), epidural (71% vs 35%) and episiotomy (42% vs 23%). Severe perineal trauma (defined as third and fourth degree perineal trauma) was slightly lower in a private hospital in first time mothers (4.7% vs 5.4%) (Table 1).

Amongst the total population of multiparous women (403,429 women), 28,703 low risk multiparous women gave birth in private hospitals and 99,212 low risk multiparous women gave birth in public hospitals The rate of obstetric intervention was significantly higher amongst those who gave birth in private hospitals in NSW compared to those who gave birth in public hospitals when all interventions for specific medical reasons were removed. Low risk multiparous women who gave birth in private hospitals had higher rates of induction for no medical reason (32% vs

For peer review only - http://bmjopen.bmj.com/site/about/guidelines.xhtml

10%), instrumental birth (8% vs 3%), epidural (34% vs 10%) and episiotomy (16% vs 5.%) and similar rates of severe perineal trauma (0.9%). The caesarean section rate still remained higher in the private cohort (5.9% vs 4.5%) though this was mostly associated with elective caesarean section (Table 2).

Perinatal characteristics, interventions and outcomes

There was no difference in birth weight between babies born in a private and public hospital. Babies born in a private hospital were significantly more likely to be born at 37, 38, 39 and 40 weeks and less likely to be born at 41 weeks gestation (Figure 1.).

Babies of primiparous women born in a private hospital were less likely to have an Apgar of <7 at five minutes (AOR 1.34 95% CI 1.77-1.530; p<0.001) or not to be resuscitated (AOR 0.354 95% CI 0.354-0.374; p<0.001). Babies born to low risk primiparous women in a private hospital were no more likely to be admitted to special care and/or neonatal intensive care (AOR 1.03 95% CI 0.984-1.075; p 0.210) and were less likely to have their baby transferred to another hospital (AOR 7.55 95% CI 6.522-8.738; p<0.001). There was no difference in the perinatal mortality rate for babies of primiparous women born in private or public hospitals (AOR 1.49 95% CI 0.926-2.410; p 0.100) (Table 3). Similar outcomes were seen for babies born to multiparous women in private and public hospitals, except there was no difference in the incidence of Apgars of <7 at five minutes (Table 4).

Reason for birth admission of neonate

We examined neonatal morbidity as coded on the neonatal birth admission record and found fewer babies overall had a morbidity record in the private sector compared to the public sector (40% vs 53.9%) due to the added complexity in the public sector. There were however some significant differences noted under the main ICD-10-AM Grouping *Certain conditions originating in the perinatal period (P00-P96)*. Babies born in a private hospital were more likely to have been affected by a forceps or vacuum delivery and were more likely to have trauma to the

For peer review only - http://bmjopen.bmj.com/site/about/guidelines.xhtml

scalp (8.1% vs 4.1%), intrauterine hypoxia (4.3% vs 2.2%), jaundice (11.7% vs 2.9%), minor cardiac murmurs (0.6% vs 0.3%), conjunctivitis (7.5% vs 2.3%), respiratory conditions (2.4% vs 1.1%), temperature regulation issues (5.2% vs 2.4%) feeding difficulties (9.6% vs 4.3%), carbohydrate metabolism issues (2.2% vs 1.0%), vomiting (1.4% vs 0.7%) and be circumcised during the birth admission (6.7% vs 0.2%). Babies born in a public hospital were more likely to be admitted for observation and evaluation (11.8% vs 9.4%) have prophylactic antibiotics (1.0% vs 0.4%) and be admitted for socioeconomic circumstances (eg. housing, distance, adoption, assumption of care) (1.1% vs 0.1%) (Table 5)

Reason for birth re-admission of neonate up to 28 days of age

We examined the reasons for transfer or readmission of babies up until 28 days of age and found, that though the numbers are small, more babies born in private hospitals were readmitted compared to babies born in a public hospital (0.95% vs 0.65% (Table 6). Babies born in private hospitals were more likely to be readmitted for infectious diseases (18.1% vs 14.4%), endocrine, nutritional and metabolic disorders (6.4% vs 3%), behavioural disorders (4.8% vs 2.5%), sleep disorders (2.6% vs 0.6%), hypoglycaemia (2% vs 0.9%), birth trauma such as cephalohaematoma (2.0% vs 0.9%); trauma involving the scalp (4.4% vs 2.1%) and excessive crying (12.1% vs 7.8%). Babies born in public hospitals were more likely to be readmitted with respiratory disorders (23.3% vs 8%), injury and poisoning (eg burns) (6.2% vs 2.2%), for observation (6.8% vs 4.9%), antibiotic therapy (3.3% vs 0.7%) and socioeconomic circumstances (housing, distance, adoption, assumption of care (13.5% vs 3.9%).

Combined birth and readmission neonatal morbidity for selected codes

When we combined major birth and readmission morbidities for key selected codes we found that in the first 28 days following birth, babies born in private hospitals were significantly more likely to be admitted for feeding difficulties (9.6% vs 4.4%), circumcision (6.7% vs 0.3%), birth trauma (mostly scalp trauma) (12.2% vs 6.6%), hypoxia (4.2% vs 2.2%), respiratory disorders (3% vs 1.4%) and seep/behavioural issues (0.5% vs

For peer review only - http://bmjopen.bmj.com/site/about/guidelines.xhtml

0.2%). Babies born in public hospitals were more likely to be admitted for socioeconomic circumstances such as housing, distance, adoption or assumption of care (1.2% vs 0.2%) and prophylactic antibiotics (1.1% vs 0.4%) (Table 7 & Figure 2).

Discussion

Intervention rates

Despite being an extremely low risk cohort, less than half the primiparous women in this study giving birth in a private hospital had a normal vaginal birth (45% vs 65%); this was 20% lower than in the public cohort. One in five primiparous women giving birth in a private hospital were induced and nearly one in two had an episiotomy. For low risk multiparous women giving birth in a private hospital nearly one in three were induced. The trend for higher intervention rates has been reported for low risk women giving birth in the private sector in Australia previously and continues to show an increase [9, 14, 15]. In a recent publication we showed that the rate of caesarean section had increased in both the private and public sector in the past decade in low risk women [9]. It has been argued in a previous publication that these high intervention rates in the private sector led to better perinatal outcomes than in the public sector [2]. This publication received significant criticism in letters to the editor [3, 16, 17] for several methodological flaws, including most significantly the failure to adjust for low birth weight, inadequate ascertainment of congenital abnormalities and failure to look at perinatal morbidity. In this study we included only low risk women, adjusting for maternal age and gestational age differences. We also removed all babies with congenital abnormalities from this data set. We found that the perinatal mortality rate was not statistically different when the populations were matched in this data set for maternal risk.

Neonatal resuscitation and admission to SCN/NICU

We found that babies born in a private hospital were much more likely to experience some form of resuscitation, in particular twice the rate of suctioning at birth. Routine suctioning for infants born with clear and/or meconium stained amniotic fluid is not recommended [18] as it can cause a bradycardia [19] and there is no evidence of benefit. We are unsure why such a high rate of newborn suctioning continues in the

For peer review only - http://bmjopen.bmj.com/site/about/guidelines.xhtml

For peer review only

private sector. While rates of Apgar scores of ≤7 at five minutes were slightly higher amongst primiparous women who gave birth in public hospital (but not multiparous women), and this has been demonstrated in another recent Australian publication [15], overall the babies were no more likely to be admitted to SCN/NICU compared to babies born in private hospitals.

Neonatal admission and readmission

We found some interesting differences in morbidity however when examining morbidity attached to the birth admission and readmission to hospital in the first 28 days for codes that may be associated with the higher rates of obstetric intervention in the private sector and a different sociodemographic profile in the public sector. While increasingly preterm babies >35 weeks/ and or >2.2k and some cases of jaundice may be managed at the bedside in some hospitals, this is less likely to occur in a private hospital. It is more likely to occur in large maternity units.

Birth trauma, in particular injuries to the scalp, were twice as high in the private sector and these are generally associated with instrumental birth, including vacuum extraction [20-22]. With more women (nearly one in three primiparous women) experiencing an instrumental birth in the private cohort and one in five women in the public sector this is not surprising. Birth trauma is associated with a longer hospital stay and increased risk of admission to SCN/NICU as well as higher rates of neonatal morbidity including neurological morbidity (hypotonia, jitteriness, convulsions and hypoxic ischaemic encephalopathy) and jaundice [22].

Jaundice was observed to be higher in the private sector, which may be related to several factors, such as the elective delivery of babies at an earlier gestation, the increased scalp trauma due to the high instrumental birth rate, as discussed above, and potential breastfeeding difficulties due to higher use of epidural analgesia. Jaundice has been associated with birth trauma, in particular delivery by vacuum extraction, and problems with feeding, especially supplementary feeding [22, 23]. Earlier gestational age <39 weeks has also be found to be associated with jaundice, with this decreasing with each week of additional gestation [24]. The gestational age of babies born in private hospitals in this

For peer review only - http://bmjopen.bmj.com/site/about/guidelines.xhtml

study was significantly lower than in the public sector due to the high rates of non-medical induction of labour and non-medically indicated caesarean section before the onset of labour.

While there have been studies associating difficulties with breastfeeding and higher rates of jaundice, the recent publication from the Universal Screening for Hyperbilirubinemia Study Group found this was a minimal risk factor.

Twice as many babies who were born in a private hospital in this study were admitted or re-admitted with feeding problems compared to babies born in a public hospital. Feeding difficulties are associated with operative birth interventions and being early term [25, 26]. Breastfeeding outcomes are positively associated with uncomplicated unassisted vaginal birth where the mother and infant remain together and breastfeeding is started within an hour of the birth and following skin-to-skin contact. Interventions during labour and birth can impact on the initiation and duration of breastfeeding. Caesarean section [27-30], instrumental birth [31], epidural anaesthesia and opioid analgesia [32-34] use have all been associated with breastfeeding difficulties. All these birth interventions were higher in the private cohort in this study. In addition early term birth, which is mainly due to induction of labour and elective caesarean section (35, 36) is associated with increased breastfeeding difficulties along with other serious morbidities [37]. Unmedicated newborns are more highly aroused immediately following the birth [38] and able to breastfeed without assistance if given skin to skin contact and freedom from intrusive procedures [39, 40]. Following caesarean section there can be a significantly longer period of time until a mother touches and holds her newborn compared to an unassisted vaginal birth [41].

In a previous paper [6] using national Australian population data we found that among low-risk women who had an unassisted vaginal birth with spontaneous onset of labour and no labour augmentation, the odds of admission to neonatal intensive care or special care nursery were significantly increased when the baby was 37 weeks gestation at the time of birth compared to later gestations. Some claim that during the final weeks of gestation the fetal brain goes through a marked increase in mass and nerve growth (corticoneurogenesis) which may be best left

For peer review only - http://bmjopen.bmj.com/site/about/guidelines.xhtml

undisturbed [42]. In this study low risk women giving birth in private hospitals in NSW were much more likely to give birth at earlier gestations than their public hospital counterparts for every week up to and including 40 weeks, but they were significantly less likely to deliver at 41 weeks. This may also help to explain why more babies born in a private hospital were readmitted with, respiratory, feeding, jaundice and sleep and behavioural problems. However there is also evidence that there are increased adverse perinatal outcomes for babies born following 41 completed weeks, but we did not examine this population [43].

Circumcision

Babies born in a private hospital were significantly more likely to be circumcised in the first 28 days of life (around 23 times more likely). This may be due to different information being given in private hospitals about the procedure or easy access to providers who perform the procedure. Circumcision rates are estimated to be between 10-20% in Australia [44] and are decreasing. A recent position statement of the Royal Australian College of Physicians states “that the frequency of diseases modifiable by circumcision, the level of protection offered by circumcision and the complication rates of circumcision do not warrant routine infant circumcision in Australia and New Zealand” [44].

Socioeconomic circumstances

The difference in the socioecononomic status of the women giving birth in public compared to private hospitals appears is demonstrated by the significantly higher rates of public hospital babies with a morbidity attached to the birth admission or readmission in the first 28 days for socioeconomic circumstances, including housing, distance, adoption and assumption of care. This again confirms what is already known that the two populations are very different sociodemographically with greater disadvantage in the public sector.

For peer review only - http://bmjopen.bmj.com/site/about/guidelines.xhtml

Limitations

Our study is limited to providing a snapshot of perinatal outcomes in one state in Australia in a defined time period for women who have no indicated risk at birth. However, this study provides useful data following on from our previous paper looking at obstetric intervention in private and public hospitals in NSW providing the reader with a detailed picture of perinatal mortality and morbidity. The advantages of using population-based datasets such as the PDC and the linkage to four other population-based databases include the size of the sample and the high level of accuracy of a validated dataset. The limitations are the restricted number of variables that are included and the scarcity of specific information on potential influencing variables. A small number of cases with a low linkage rate (0.3%) were not included and so there is the possibility of missing adverse outcomes. A previous study showed that where stillbirths are excluded due to low linkage these are at lower gestational ages and not term infants as were the focus in this study [45]. Previous validation studies have reported high levels of data accuracy for the majority of diagnoses and procedures conducted during labour and delivery in the state-wide data base [46, 47], although the recording of medical conditions and smoking are overall generally underreported [46, 48]. Having a linked data set provides a much richer picture than we have had previously of the morbidity and mortality associated with birth interventions. While we could not control for obesity due to lack of data, women who have private health insurance have lower rates of obesity and higher socioeconomic status, hence these health disadvantages are most likely over represented in the public women [49]. There are also several other socio-demographic factors we could not control for, such as education and income, that increase risk for the women giving birth in public hospitals. This study can only provide an overview of possible associations between obstetric interventions and neonatal outcomes and does not imply causality, which could be better obtained from prospective cohort studies.

For peer review only - http://bmjopen.bmj.com/site/about/guidelines.xhtml

Conclusion

The continual rise in obstetric intervention for low risk women in Australia may be contributing to increased morbidity for healthy women and babies and higher cost of health care. The fact that these procedures which were initially life-saving are now so commonplace and do not appear to be associated with improved rates of perinatal mortality or morbidity demands close review. Early term delivery and instrumental births may be associated with increased morbidity in neonates and this requires urgent attention.

For peer review only

For peer review only - http://bmjopen.bmj.com/site/about/guidelines.xhtml

Contributorship Statement

HD led the study and wrote the paper. ST helped in constructing the study design and writing the paper. MT helped in constructing the study design. AB helped in writing the paper. CB gave biostatistical support and helped in writing the paper. CT helped with study design, analysis and writing of the paper.

Competing Interests

No, there are no competing interests

Data Sharing Statement

There is no additional data available

References

1 Li Z, Zeki R, Hilder L, et al. Australia's mothers and babies 2010. Perinatal statistics series no. 27. Cat. no. PER 57. In: Unit ANPEaS, editor. Canberra2012.

2 Robson SJ, Laws P, Sullivan EA. Adverse outcomes of labour in public and private hospitals in Australia: a population based descriptive study. The Medical Journal of Australia 2009;190(9):474-7.

3 Tracy S, Welsh A, Dahlen H, et al. Letter to the Editor re Robson SJ, Laws P, Sullivan EA. Adverse outcomes of labour in public and private hospitals in Australia: a population-based descriptive study. Med J Aust 2009; 190: 474-477. Medical Journal of Australia. 2009;191(10):579-80.

For peer review only - http://bmjopen.bmj.com/site/about/guidelines.xhtml

4 Clark E.A.S, Silver RM. Long-term maternal morbidity associated with repeat caesarean delivery. American Journal of Obstetrics and Gynecology. 2011;S2(December).

5 Hyde MJ, Mostyn A, Modi N, et al. The health implications of birth by caesarean section. Biological Reviews. 2012;87(1):229-43.

6 Tracy S, Tracy M, Sullivan E. Admission of Term Infants to Neonatal Intensive Care: A Population-Based Study. Birth 2007;34(4):301-7.

7 Tracy S, K , Tracy M. Costing the cascade: estimating the cost of increased obstetric intervention in childbirth using population data. British Journal of Obstetrics and Gynaecology. 2003;110:717-24.

8 Allen VM, O'Connell CM, Farrell SA, et al. Economic implications of method of delivery. Am J Obstet Gynecol 2005;193(1):192-7.

9 Dahlen HG, Tracy S, Tracy M et al. Rates of obstetric intervention among low-risk women giving birth in private and public hospitals in NSW: a population-based descriptive study. BMJ Open. 2012;2:e001723 doi:10.1136/bmjopen-2012-001723.

10 Roberts C, L,, Tracy S, Peat B. Rates of obstetric intervention among private and public patients in Australia: population based descriptive study. British Medical Journal. 2000;312:137-41.

11 Buist R. Letter to the Editor: Private Obstetrics-again. BMJ Open. 2012;2(5):e001723 doi:10.1136/bmjopen-2012-.

12 CHeReL. Centre for Health Record Linkage (CHeReL). Quality Assurance Report 2012. http://wwwcherelorgau/media/24160/qa_report_2012pdf. 2012.

13 Lain SJ, Hadfield RM, Raynes-Greenow CH, et al. Quality of data in perinatal population health databases: a systematic review. Med Care. 2012;50(4):e7-e20.

14 Roberts CL, Tracy S, Peat B. Rates for obstetric intervention among private and public patients in Australia: population based descriptive study. British Medical Journal. 2000 2000;321(7254):137-41.

15 Einarsdóttir K, Stock S, Haggar F, et al. Neonatal complications in public and private patients: a retrospective cohort study. BMJ Open. 2013;Downloaded from bmjopen.bmj.com on November 11, 2013.

16 Evans N, Malcolm G, Gordon A. Letter to the Editor re Adverse outcomes of labour in public and private hospitals in Australia. MJA. 2009;191(10):579.

17 Chambers D. Letter to the Editor re Adverse outcomes of labour in public and private hospitals in Australia. MJA. 2009;191(10):578-9.

For peer review only - http://bmjopen.bmj.com/site/about/guidelines.xhtml

18 Wyllie J. Recent changes to UK newborn resuscitation guidelines. Arch Dis Child Fetal Neonatal Ed. 2012;97(1):F4-F7.

19 Kannapiran R, MKennea N. Resuscitation of the newborn. Obstetrics, Gynecology & Reproductive Medicine. 2012;22(4):92-7.

20 Doumouchtsis SK, Arulkumaran S. Head injuries after instrumental vaginal deliveries. Current Opion in Obstetrics and Gynaecology. 2006;18:129-34.

21 Towner D, Castro MA, Wilkens EE, et al. Effect of Mode of Delivery in Nulliparous Women on Neonatal Intracranial Injury. The New England Journal of Medicine 1999;341:1709-14.

22 Linder N, Linder I, Fridman E, et al. Birth trauma – risk factors and short-term neonatal outcome. The journal of Maternal-fetal & Neonatal Medicine. 2013;DOI: 10.3109/14767058.2013.789850.

23 Bertini G, Dani C, Tronchin M, et al. Is breastfeeding really favouring early neonatal jaundice? Pediatrics. 2006;107(3):e41.

24 Bhutani VK, Stark AR, Lazzeroni LC, et al. Predischarge Screening for Severe Neonatal Hyperbilirubinemia Identifies Infants Who Need Phototherapy. The Journal of Pediatrics. 2013;162(3):477-82.

25 Young PC, Korgenski K, Buchi KF. Early Readmission of Newborns in a Large Health Care System. Pediatrics. 2013;131(5):e1538-e44.

26 Brown A, Jordan S. Impact of birth complications on breastfeeding duration: an internet survey. Journal of Advanced Nursing. 2012;69(4):828-39.

27 Chapman D.J, Perez-Escamilla R. Identification of risk factors for delayed onset oflactation. Journal of the American Dietetic Association. 2003;99:450-4.

28 Dewey K.G., Nommsen-Rivers L.A., Heinig M.J, et al. Risk factors for suboptimal infant breastfeeding behavior, delayed onset of lactation and excess neonatal weight loss. Pediatrics 2003;112(3):607-19.

29 Scott J.A., Binns C.W, Oddy W.H. Predictors of delayed onset of lactation. Maternal and Child Nutrition. 2007;3(3):186-93.

30 Jordan S., Emery S., Watkins A., et al. Associations of drugs routinely given in labour with breastfeeding at 48 hours: analysis of the Cardiff Births Survey. British Journal of Gynecology. 2009;116(12):1622-9.

31 Leung G.M, Lam T.H, Ho L.M. Breast-feeding and its relation to smoking and mode of delivery. Obstetrics and Gynecology 2002;99(5):785-94.

For peer review only - http://bmjopen.bmj.com/site/about/guidelines.xhtml

32 Jordan S. Infant feeding and analgesia in labour: the evidence is accumulating. International Breastfeeding Journal Research. 2006;1(25):doi: 10.1186/746-4358-1-25.

33 Henderson J.J., Dickinson J.E., Evans S.F., et al. Impact of intrapartum epidural analgesia on breast- feeding duration. Australian and New Zealand Journal of Obstetrics and Gynaecology. 2003;43(5):372–7.

34 Beilin Y., Bodian C., Weiser J., et al. Effect of labor analgesia with and without fentanyl on infant breastfeeding: a prospective, randomized double-blind study. Anesthesiology. 2005;103(6):1211– 7.

35 Ohnsorg T, Schiff J. Preventing elective induction before 39 weeks. , 93(11), 44-46. Minnesota Medicine. 2010;93(11):44-6.

36 Oshiro BT, Henry E, Wilson J, et al. Decreasing elective deliveries before 39 weeks of gestation in an integrated health care system. Obstetrics & Gynecology. 2009;113:804-11.

37 Reddy UM, Ko CW, Willinger M. “Early term” births (37-38 weeks) are associated with increased mortality. American Journal of Obstetrics & Gynecology. 2006;195(S202).

38 McLaughlin FJ, O’Connor S, Deni R. Infant state and behavior during the first postpartum hour. The Psychological Record 1981;31:455–8.

39 Widstrom A-M, Ransjo-Arvidson AB, Christensson K, Matthiesen A- S, Winberg J, Uvnas-Moberg K. Gastric suction in healthy newborn infants. Effects on circulation and developing feeding behaviour. Acta Paediatr. 1987;76:566–72.

40 Righard L. How do newborns find their mother’s breast? . Birth. 1995;22:174–5.

41 Fisher J, Astbury J, Smith A. Adverse psychological impact of opera- tive obstetric interventions: a prospective study. Aust NZ J Psychiatry 1997;31:728–38.

42 Adams-Chapman I. Insults to the developing brain and impact on neurode- velopmental outcome. Journal of Communication Disorders. Journal of Communication Disorders. 2009;42:256-62.

43. Gülmezoglu AM, Crowther CA, Middleton P, et al. Induction of labour for improving birth outcomes for women at or beyond term. *CochraneDatabase of Systematic Reviews* 2012;Issue 6. Art.No.:CD004945. DOI: 10.1002/14651858.CD004945.pub3.

44 The Royal Australian College of Physicians. Circumcision of infant males. In: Physicians TRACo, editor. Sydney: The Royal Australasian College of Physicians; 2010.

For peer review only - http://bmjopen.bmj.com/site/about/guidelines.xhtml

45 Bentley JP, Ford JB, Taylor LK, et al. Investigating linkage rates among probabilistic linked births and hospital records. BMC Medical Research Methodology. 12:149, 2012.

46 Taylor L, Travis S, Pym M, Olive E, et al. How useful are hospital morbidity data for monitoring conditions occurring in the perinatal period? Australian & New Zealand Journal of Obstetrics & Gynaecology. 2005;45:36 –41.

47 Roberts C, Bell J, Ford J, Morris J. Monitoring the quality of maternity care: how well are labour and delivery events reported in population health data? . Paediatirc and Perinatal Epidemiology. 2008;23:144-52.

48 Thornton C, Makris A, Ogle R, Hennessy A. Generic obstetric database systems are unreliable for reporting the Hypertensive Disorders of Pregnancy. Australian and New Zealand Journal Obstetrics and Gynaecology. 2004;44(505-509).

49 Finkelstein A, Fiebelkorn IC, Wang G. National medical Spendig attributable to overweight and obesity: How much and who's paying. Health Affairs, 2003 Project HOPE–The People-to-People Health Foundation, Inc., 219-226. 2003.

Table 1. Maternal characteristics, interventions and outcomes for low risk primiparous women in NSW (2000-2008)

**Low risk Primiparous women**	**Private Hospital (n=29597)**	**Public Hospital (n=79792)**
**Maternal age (years)**		
20-24	6.2%	28.9%
25-29	39.9%	40.9%
30-34	53.9%	30.2%
Weeks gestation at delivery		

For peer review only - http://bmjopen.bmj.com/site/about/guidelines.xhtml

37	4.5%	4.3%
38	15.4%	11.8%
39	27.8%	25.0%
40	43.2%	39.7%
41	9.1%	19.2%
**Type of labour**		
Spontaneous	71.9%	89.9%
Induced	19.2%	7.1%
No labour	8.9%	3.0%
**Delivery**		
Normal vaginal	44.9%	64.8%
Forceps	11.5%	6.7%
Vacuum	18.9%	12.9%
Total caesarean section	24.7%	15.6%
Caesarean section (after labour)	15.9%	12.6%
Caesarean section before the onset of labour	8.8%	3.0%
**Epidural**	70.8%	35.4%
**Episiotomy**	42.4%	23.3%

For peer review only

**Severe perineal trauma**	4.7%	5.4%

Table 2. Maternal characteristics, interventions and outcomes for low risk multiparous women in NSW (2000-2008)

**Low risk Multiparous women**	**Private Hospital (n=28703)**	**Public Hospital (n=99212)**
**Maternal age (years)**		
20-24	2.1%	16.8%
25-29	25.8%	38.8%
30-34	72.1%	44.4%
Weeks gestation at delivery		
37	4.1%	4.0%
38	18.7%	13.0%
39	31.6%	26.9%
40	40.2%	40.8%
41	5.4%	15.3%
**Type of labour**		
Spontaneous	64.0%	87.4%
Induced	32.1%	10.1%

For peer review only - http://bmjopen.bmj.com/site/about/guidelines.xhtml

No labour	3.9%	2.5%
**Delivery**		
Normal vaginal	86.1%	92.7%
Forceps	1.9%	0.7%
Vacuum	6.1%	2.1%
Total caesarean section	5.9%	4.5%
Caesarean section after labour	2.0%	2.0%
Caesarean section before the onset of labour	3.9%	2.5%#
**Epidural**	34.4%	9.5%
**Episiotomy**	16.2%	5.1%
**Severe perineal trauma**	0.9%	0.9%

For peer review only

Table 3. Perinatal outcomes adjusted for maternal age and gestation at birth for primiparous women

	Private	Public			
	(n=29597)	(n=79791)	OR**	AOR**	p
Apgar<7 at 5 minutes	296 (1.0%)	1037 (1.3%)	1.36 (1.198-1.542)	1.34(1.177-1.530)	<0.001
Any resuscitation*	18498 (62.5%)	30560 (38.3%)	0.372 (0.363-0.382)	0.364 (0.354-0.374)	<0.001
Admitted to SCN and/or NICU	3078 (10.4%)	8139 (10.2%)	1.00 (0.964-1.049)	1.03 (0.984-1.075)	0.210
Transferred	178 (0.6%)	3351 (4.2%)	7.30 (6.289-8.403)	7.55 (6.522-8.738)	<0.001
Total Perinatal Mortality	22 (0.74/1000)	85 (1.06/1000)	1.40 (0.930-2.006)	1.49 (0.926-2.410)	0.100

*Any resuscitation includes: Suction, oxygen, IPPR by bag and mask, Intubation and IPPR, external cardiac massage and ventilation and other

** Private hospital is the reference category

For peer review only

Table 4. Perinatal outcomes adjusted for maternal age and gestation at birth for multiparous women

	Private (n=28703)	Public (n=99212)	OR**	AOR**	p
Apgar<7 at 5 minutes	67 (0.2%)	149 (0.2%)	1.56 (0.980-2.098)	1.42 (0.96-2.21)	0.562
Any resuscitation*	14820 (51.6%)	29867 (30.1%)	0.404 (0.393-0.415)	0.399 (0.388-0.460)	<0.001
Admitted to SCN and/or NICU	1775 (6.2%)	5870 (5.9%)	0.957 (0.906-1.011)	1.027 (0.970-1.087)	0.363
Transferred	232 (0.8%)	4375 (4.4%)	5.661 (4.958-6.465)	6.516 (5.699-7.450)	<0.001
Total Perinatal Mortality	17 (0.59/1000)	76 (0.77/1000)	1.294 (0.765-2.189)	1.294 (0.750-2.232)	0.355

For peer review only - http://bmjopen.bmj.com/site/about/guidelines.xhtml

*Any resuscitation includes: Suction, oxygen, IPPR by bag and mask, Intubation and IPPR, external cardiac massage and ventilation and other

** Private hospital is the reference category

Table 5. Morbidity associated with birth admission coded on neonatal birth admission record

		Private		Public		p
**Total neonates with morbidity codes**		**23330**	**40.0%**	**96508**	**53.9%**	<0.001
**ICD-10-AM Grouping**						
**Certain conditions originating in the perinatal period (P00-P96)**						
	Fetus and newborn affected by maternal infectious and parasitic diseases	41	0.2%	989	1.0%	<0.001
	Fetus and newborn affected by forceps delivery	473	2.0%	1108	1.1%	<0.001
	Fetus and newborn affected by delivery by vacuum extractor	511	2.2%	1509	1.6%	<0.001
	**Birth trauma (all body systems)**	**2948**	**12.6%**	**6447**	**6.7%**	<0.001
	- specifically to scalp (included in above total)	1880	8.1%	3965	4.1%	<0.001
	Intrauterine hypoxia	993	4.3%	2170	2.2%	<0.001
	Other specified respiratory conditions of newborn	562	2.4%	1015	1.1%	<0.001
	Benign and innocent cardiac murmurs in newborn	139	0.6%	303	0.3%	<0.001
	Neonatal conjunctivitis specific to the perinatal period	1740	7.5%	2267	2.3%	<0.001
	**Jaundice related conditions**	**2728**	**11.7%**	**5166**	**2.9%**	<0.001
	Transitory disorders of carbohydrate metabolism specific to fetus and newborn	502	2.2%	942	1.0%	<0.001
	Temperature regulation	1214	5.2%	2275	2.4%	<0.001
	Vomiting in newborn	320	1.4%	693	0.7%	<0.001

For peer review only - http://bmjopen.bmj.com/site/about/guidelines.xhtml

	Feeding difficulties	2231	9.6%	4157	4.3%	<0.001
**factors influencing health status and contact with health services (Z00-Z99)**						
	Routine and ritual circumcision	1552	6.7%	187	0.2%	<0.001
	Observation and evaluation of newborn	2187	9.4%	11372	11.8%	<0.001
	Prophylactic chemotherapy (antibiotics)	93	0.4%	935	1.0%	<0.001
	Socioeconomic circumstances (housing, distance, adoption, assumption of care)	32	0.1%	1020	1.1%	<0.001
		3184	13.7%	49988	51.8%	

Table 6. Morbidity associated with readmission of the baby ≤28 days of age

		**Private**		**Public**		**p**
**Total admissions**		**689**		**1507**		
**Total neonates**		**552 (0.95%)**		**1155 (0.65%)**		<0.001
**ICD-10-AM Grouping**						
**Certain infectious and parasitic diseases**		**121**	**18.1%**	**217**	**14.4%**	<0.001
**Endocrine nutritional and metabolic diseases**		**30**	**4.4%**	**38**	**2.5%**	<0.001
	Volume depletion	18	2.6%	12	0.8%	<0.001

For peer review only - http://bmjopen.bmj.com/site/about/guidelines.xhtml

**Mental and behavioural disorders**		**33**	**4.8%**	**30**	**2.0%**	<0.001
	Non-organic hypersomnia	17	2.5%	19	1.3%	<0.001
**Diseases of the nervous system**		**34**	**4.9%**	**38**	**2.5%**	<0.001
	Disorders of the sleep wake schedule	18	2.6%	9	0.6%	<0.001
**Diseases of the ear and mastoid process**		**19**	**2.8%**	**23**	**1.5%**	<0.001
**Diseases of the respiratory system**		**155**	**8.0%**	**351**	**23.3%**	<0.001
	Acute obstructive laryngitis (croup)	6	0.9%	16	1.1%	=0.76
	Acute upper respiratory infection unspecified	16	2.3%	52	3.5%	0.84
	Pneumonia	6	0.9%	19	1.3%	=0.86
	Acute bronchiolitis	70	10.2%	175	11.6%	=0.17
	Unspecified acute lower respiratory tract infection	3	0.4%	12	0.8%	0.52
**Diseases of the digestive system**		**53**	**7.7%**	**92**	**6.1%**	<0.001
	Gastro-oesophageal reflux disease	26	3.8%	37	2.5%	0.01
**Certain conditions originating in the perinatal period**		**474**	**68.8%**	**1011**	**67.1%**	<0.001
	Cephalohaematoma due to birth trauma	14	2.0%	13	0.9%	<0.01
	Total birth trauma to scalp	30	4.4%	32	2.1%	<0.001
	Intrauterine hypoxia	4	0.6%	25	1.7%	0.43

For peer review only - http://bmjopen.bmj.com/site/about/guidelines.xhtml

	Other neonatal hypoglycaemia	14	2.0%	14	0.9%	<0.01
	Other transitory neonatal electrolyte and metabolic disturbances	5	0.7%	21	1.4%	=0.69
	Fever of newborn	5	0.7%	18	1.2%	0.92
	Feeding problems of newborn	40	5.8%	100	6.6%	=0.31
	**Neonatal jaundice**	**193**	**28%**	**90**	**6%**	<0.001
**Symptoms, signs and abnormal findings not elsewhere classified**		**198**	**28.7%**	**340**	**22.6%**	<0.001
	Fever	19	2.8%	27	1.8%	=0.02
	Feeding difficulties and mismanagement	25	3.6%	37	2.5%	<0.01
	Excessive crying	83	12.1%	117	7.8%	<0.001
**injury, poisoning and certain other consequences of external causes**		**15**	**2.2%**	**94**	**6.2%**	<0.001
	Burns	0	0.0%	30	2.0%	<0.001
**factors influencing health status and contact with health services**		**192**	**28.9%**	**691**	**45.9%**	<0.001
	Observation	34	4.9%	102	6.8%	=0.92
	Other prophylactic antibiotic therapy	5	0.7%	49	3.3%	<0.01
	Routine and ritual circumcision	68	9.9%	119	7.9%	<0.001
	Attention to surgical dressings and sutures	0	0.0%	28	1.9%	

For peer review only - http://bmjopen.bmj.com/site/about/guidelines.xhtml

	Socio-economic circumstances (housing, distance, adoption, assumption of care)	22	3.9%	156	13.5%	<0.001

For peer review only

For peer review only - http://bmjopen.bmj.com/site/about/guidelines.xhtml

Table 7. Combined birth and readmission neonatal morbidity for selected codes

	Private	Public	P*
Total feeding difficulties	2314 (9.6%)	4306 (4.4%)	<0.0001
Total circumcision	1620 (6.7%)	306 (0.3%)	<0.0001
Total socio-economic circumstances	54 (0.2%)	1176 (1.2%)	<0.0001
Total birth trauma	2922 (12.2%)	6492 (6.6%)	<0.0001
Total hypoxia	997 (4.2%)	2195 (2.2%)	<0.0001
Total jaundice	2818 (11.8%)	5359 (5.5%)	<0.0001
Total respiratory	717 (3.0%)	1366 (1.4%)	<0.0001
Total sleep/behavioural issues	118 (0.5%)	145 (0.2%)	<0.0001
Prophylactic antibiotics	98 (0.4%)	982 (1.1%)	<0.001

*chi-square

Figure 1. Comparison of gestation at delivery between all women stratified by hospital type

Figure 2. Birth trauma as a percentage of all births in Private and Public Hospitals

For peer review only

Rates of obstetric intervention and associated perinatal mortality and morbidity amongst low risk women giving birth in private and public hospitals in NSW (2000-2008): A linked data population based cohort study.

Hannah G. Dahlen RN, RM, BN (Hons), MCommN, PhD, FACM

Professor of Midwifery

School of Nursing and Midwifery

Family and Community Health Group

Locked Bag 1797

Penrith South DC

NSW 2751

Australia

Ph: +61 2 96859118

Fax:+61 2 96859599

h.dahlen@uws.edu.au

Professor Sally Tracy

Professor Midwifery

Professor of Midwifery

University of Sydney,

Royal Hospital for Women, Sydney

For peer review only

For peer review only - http://bmjopen.bmj.com/site/about/guidelines.xhtml

Tel: 0420277106

Dr Mark Tracy PhD MSc (Epi Lond) FRACP(Paeds) MBBS DLSTHM

Senior Newborn Intensive Care Specialist and HOD

CNBC Westmead Hospital

Senior Clinical Lecturer Paediatrics and Child Health

University of Sydney

Tel (02) 9845 6568

Conjoint Professor Andrew Bisits MB bs(UNSW)FRANZCOG ,MMed Stat ,Dip Clin Epi

Acting Medical Co Director of Maternity, Lead Clinician Birthing Suite

Royal Hospital for Women

Randwick NSW 2033

0419418492

Mr Chris Brown, BSc, MBiostat

Biostatistician

NHMRC Clinical Trials Centre

For peer review only - http://bmjopen.bmj.com/site/about/guidelines.xhtml

Locked Bag 77

Camperdown NSW 1450

Dr Charlene Thornton BN, Grad.Dip.Mid, M Sc Med (Research), M Sc Med (Clin Epi), PhD

Post Doctoral Research Fellow

School of Nursing and Midwifery

Family and Community Health Group

Locked Bag 1797

Penrith South DC

NSW, Australia

Keywords: low risk, low risk, induction, vaginal birth, obstetric care, caesarean, birth trauma

Word count excluding title page, abstract, figures and tables: 4531

For peer review only

Rates of obstetric intervention and associated perinatal mortality and morbidity amongst low risk women giving birth in private and public hospitals in NSW (2000-2008): A linked data population based cohort study.

Abstract

Objectives: To examine the rates of obstetric intervention and associated perinatal mortality and morbidity in the first 28 days amongst low risk women giving birth in private and public hospitals in NSW (2000-2008) using linked data.

Design: Linked data population based cohort study involving five data sets.

Setting: New South Wales, Australia.

Participants: 691,738 women giving birth to a singleton baby during the period 2000 to 2008.

Main outcome measures: Rates of neonatal resuscitation, perinatal mortality, neonatal admission following birth and readmission to hospital in the first 28 days of life in public and private obstetric units.

Results: Rates of obstetric intervention amongst low risk women were significantly higher in private hospitals, with primiparous women 20% less likely to have a normal vaginal birth compared to the public sector. Neonates born in private hospitals were more likely to be less than 40 weeks gestation compared to those born in public hospitals and more likely to have some form of resuscitation, less likely to have an Apgar <7 at five minutes (if primiparous) and less likely to be transferred between hospitals. Neonates born in private hospitals to low risk mothers were more likely to have a morbidity attached to the birth admission and to be readmitted to hospital in the first 28 days for birth trauma; hypoxia; jaundice; feeding difficulties; sleep /behavioural issues; respiratory conditions and circumcision. Neonates born in a private hospital were less

For peer review only - http://bmjopen.bmj.com/site/about/guidelines.xhtml

For peer review only

likely to be admitted for prophylactic antibiotics and for socioeconomic circumstances (eg. housing, distance, adoption, assumption of care). Rates of perinatal mortality were not statistically different between the two groups.

Conclusion:

High rates of obstetric intervention amongst low risk women in private hospitals appear to be associated with higher rates of certain morbidity seen in the neonate and no evidence of a reduction in perinatal mortality.

Keywords: low risk primip, low risk multip, induction, vaginal birth, obstetric care, caesarean, birth trauma

Article focus:

- To examine the rates of obstetric intervention and associated perinatal mortality and morbidity in the first 28 days amongst low risk women giving birth in private and public hospitals in NSW (2000-2008) using linked data.
- Rates of neonatal resuscitation, perinatal mortality, neonatal admission following birth and readmission to hospital in the first 28 days of life in public and private obstetric units.

Key messages

- Babies born to low risk mothers in private hospitals are more likely to require some resuscitation compared to those born in public hospitals.
- For babies born in private hospitals to low risk mothers there are higher rates of morbidity such as birth trauma, hypoxia, jaundice, feeding difficulties, sleep/behavioural issues, respiratory conditions and circumcision. For babies born in public hospitals there are higher rates of antibiotic use and admission for socio-economic reasons (eg housing, distance, adoption and assumption of care)

For peer review only - http://bmjopen.bmj.com/site/about/guidelines.xhtml

For peer review only

- Rates of perinatal mortality were not statistically different between babies born to low risk women giving birth in private and public hospitals

Strengths and limitations of this study

- The strength of this study lies in the large sample size of birth data from over half a million women and babies and the >1.1 million admissions associated with these births.
- The use of data from five population based datasets which have been linked to enhance validity and ascertainment
- Limitations are the restricted number of variables that are included and the scarcity of specific information on potential confounders.
- Body mass index and key sociodemographic risk factors could not be controlled for and this would have added risk to women giving birth in public hospitals.

Introduction

In Australia, the national statistics reveal that 32.8% (n =70,332) of women giving birth in 2011 elected private status, with 29.9% of women giving birth in private hospitals directly under private obstetric care [1]. The remaining 3% of privately insured women received a combination of midwifery and medical care in public hospitals. The remaining (n=139, 486) 65% of women receive care as public patients in public hospitals in Australia. Women who are privately insured have been reported to have better maternal and perinatal outcomes compared to women who give birth in public hospitals as public patients [2]; but it has been argued that these women tend to be less socioeconomically disadvantaged

For peer review only - http://bmjopen.bmj.com/site/about/guidelines.xhtml

and healthier [3] and therefore might be expected to have better outcomes. Arguments about the impact of private status on health outcomes are in reality complex.

What is not disputed are the much higher rates of obstetric intervention that occur in private hospitals in Australia. At a national level, the intervention rates in childbirth, such as caesarean section, are significantly higher in the private sector (43.1% vs 28.4%) and the rates of normal vaginal birth significantly lower (42.7% vs 61%) [1]. Despite the rising intervention rates over the past decade, the perinatal mortality rate overall has not shown a corresponding decline. There is also growing concern that the short and long term morbidity associated with major obstetric interventions, such as caesarean, may not be insignificant for the mother [4] and the baby [5, 6]. The cost to the tax payer of the rising intervention in childbirth is also significant [7, 8].

A recent study in New South Wales, Australia [9] found among 293,840 low-risk women, rates of obstetric intervention were highest in private hospitals and lowest in public hospitals. Low-risk primiparous women giving birth in a private hospital compared to a public hospital had higher rates of induction (31% vs 23%); instrumental birth (29% vs 18%); caesarean section (27% vs 18%), epidural (53% vs 32%), episiotomy (28% vs 12%) and lower normal vaginal birth rates (44% vs 64%). Low-risk multiparous women had higher rates of instrumental birth (7% vs 3%), caesarean section (27% vs 16%), epidural (35% vs 12%), episiotomy (8% vs 2%) and lower normal vaginal birth rates (66% vs 81%). Following a comparison with data from the decade previously [10], these interventions were found to have increased by 5% for women in public hospitals and by over 10% for women in private hospitals [9]. Among low-risk primiparous women giving birth in private hospitals, 15 per 100 women had a vaginal birth with no obstetric intervention compared to 35 per 100 women giving birth in a public hospital [9]. Concern was expressed that perinatal mortality and morbidity was not reported in that paper [11]. In this study we aim to examine the rates of obstetric intervention and associated perinatal mortality and morbidity attached to the birth admission and readmission to hospital in the first 28 days of life for low risk women giving birth in private and public hospitals in NSW (2000-2008).

For peer review only - http://bmjopen.bmj.com/site/about/guidelines.xhtml

Methods

Data Sources

The New South Wales Centre for Health Record Linkage conducted linkage of several datasets via the Health Record Linkage (CHeReL). Pregnancy and birth data for the time period July 1st 2000 till June 2nd 2008 of all singleton births were provided by New South Wales (NSW), Ministry of Health as recorded in the NSW Midwives Data Collection (MDC), now the Perinatal Data Collection (PDC). This population based surveillance system contains maternal and infant data on all births of greater than 400 grams birth weight and/or 20 completed weeks gestation. Hospitals are coded either as private or public in the data set. However, the data identifying women who received care in public hospitals under private accommodation status is no longer collected as it had been in the years 1996-97 and for this reason patients who are under private obstetric care in public hospitals are not able to be differentiated from their public counterparts, so for this study we analysed the data by hospital (private/public). A previous study published in 2000 [10] showed that there was a moderating factor on intervention rates when women with private insurance status gave birth in a public hospital, leading to lower intervention rates than when they gave birth in private hospitals.

The NSW PDC contains statistics on one third of all births which occur in Australia annually and provided data on maternal age, maternal hypertension, maternal diabetes, parity, private/public patient status, fetal presentation, onset of labour, gestation at birth, delivery type, Apgar scores and admission to neonatal intensive care and resuscitation details for the neonate. This dataset (NSW PDC was linked to the Admitted Patient Data Collection (APDC) for the time period July 1st 2000 – June 30th 2008. The APDC records all admitted patient services provided by NSW Public Hospitals, Public Psychiatric Hospitals, Public Multi-Purpose Services, Private Hospitals, and Private Day Procedures Centres. The APDC provided additional information, such as data on maternal medical conditions, which was used to exclude further maternal cases and was used to calculate admission and readmission details for neonates. Further linkage occurred to the NSW Registry of Births,

For peer review only - http://bmjopen.bmj.com/site/about/guidelines.xhtml

Deaths and Marriages (RBDM) and the Australian Bureau of Statistics Death Data, which provided mortality data. The NSW Register of congenital conditions provided cases of congenital conditions, as did the coding in the APDC. Any neonate (and mother pair) with a recorded congenital condition (ICD-10-AM codes Q0.0-Q99.9) on either dataset was removed from the dataset due to their high-risk status. Probabilistic data linkage techniques were utilised for data linkage and de-identified datasets were provided for analysis. Probabilistic record linkage software assigns a 'linkage weight' to pairs of records. For example, records that match perfectly or nearly perfectly on first name, surname, date of birth and address have a high linkage weight, and records that match only on date of birth have a low linkage weight. If the linkage weight is high it is likely that the records truly match, and if the linkage weight is low it is likely that the records are not truly a match. This technique has been shown to have a false positive rate of 0.3% of records [12].

Gestation is recorded at birth and is also recorded in the database according to the woman's menstrual history, usually combined with a routine scan at 12-13 weeks.

Admission to neonatal intensive care refers to admission to special care nursery (SCN) or neonatal intensive care unit (NICU).

Any resuscitation includes suction of the mouth or nostrils at birth; oxygen administered by mask; intermittent positive pressure respiration (IPPR) by bag and mask or by intubation; external cardiac massage and ventilation.

Ethical approval was obtained from the NSW Population and Health Services Research Ethics Committee, Protocol No.2010/12/291.

Subjects

We classified the low risk Primipara as a primiparous woman aged 20-34 years, who had no pre-existing or pregnancy related hypertension or diabetes, was a non-smoker, and gave birth at 37-41 completed weeks gestation to a singleton baby in a cephalic presentation within the 10th

For peer review only - http://bmjopen.bmj.com/site/about/guidelines.xhtml

and 90th centiles for birth weight. The low risk multipara was a multiparous woman aged 20-34 years, who had no pre-existing or pregnancy related hypertension or diabetes, was a non-smoker, gave birth at 37-41 completed weeks gestation to a singleton baby in a cephalic presentation within the 10th and 90th centiles for birth weight. We excluded women with a previous caesarean section or who were induced for a medical indication, or who underwent a caesarean section for a pre-existing medical indication or gave birth without a trained birth attendant (born before arrival). If a caesarean section was undertaken during labour however for non-reassuring heart rate, dystocia etc these women were included in the study. These characteristics were defined firstly from the PDC with additional medical conditions identified in the APDC being used to exclude cases.

Outcomes

Any neonatal admission including the ICD-10-AM codes Z37.0 (single live birth), Z37.1 (single stillbirth) or Z38.0 (singleton born in hospital) was deemed the birth admission and any ICD-10-AM codes referring to conditions which arise in the perinatal period (P00-P96) and those referring to factors influencing health status and contact with health services (Z00-Z99) which were included in this admission were deemed morbidities associated with the birth admission. Any other admission following this discharge from the initial birth admission to home or another hospital was deemed a readmission and included transfers to a hospital other than that where the birth occurred. When examining readmission data, all ICD-10-AM codes recorded were reviewed and those where ≥10 events occurred was marked for analysis. Events were grouped in body systems where appropriate or under headings such as infection for ease of analysis and interpretation.

Stillbirth and neonatal deaths were calculated from multiple sources but were limited to those that occurred 28 days from the birth and they were only counted once. A detected case on any of the following four datasets was deemed a death. The PDC 'Discharge status' variable or admissions in the APDC where the case mode separation was coded as 'Died' or the NSW RBDM or ABS Death Data where a death had been recorded. The maternal admission data for any admission that occurred during the pregnancy, as well as the birth admission for all cases of

For peer review only - http://bmjopen.bmj.com/site/about/guidelines.xhtml

stillbirth or neonatal death were examined to determine any maternal medical or pregnancy related condition. Reasons for stillbirth and neonatal death were taken from the principal cause noted on the death registration. If the death was not yet registered, the principal diagnosis as recorded in the neonate's birth or subsequent admission was utilised. In any case where either of these two methods did not supply a reason for death or principal diagnosis, no reason for death was recorded. This methodology of utilising multiple data sources to identify cases has been shown by Lain et al (2012) to be the most reliable way to increase ascertainment of cases (13).

Obstetric intervention was defined to include induction, epidural use, episiotomy, instrumental delivery (requiring the use of forceps or vacuum) and delivery via caesarean section.

Data analysis

The cohort was divided into primiparous and multiparous women for the primary analysis of birth outcomes. When examining neonatal status at birth and mortality odds ratios were calculated using logistic regression with and without adjustment for age and gestation. For neonatal morbidity at birth and readmission, chi-square statistics were calculated for observed events. Taking into account the size of the cohort and the number of analyses undertaken, results were considered significant at the level $p<0.01$. Analysis was undertaken with IBM SPSS v.20®

Results

Maternal characteristics, interventions and outcomes

For peer review only - http://bmjopen.bmj.com/site/about/guidelines.xhtml

For peer review only

The PDC dataset for the time period July 1st 2000 to 2nd June 2008 contained the antenatal, birth and postnatal details on 691 738 births. The APDC for the time period July 1st 2000 to 30th June 2008 (28 days from birth) contained >1.1 million admissions for the neonates of these women.

From the total population of primiparous women (288,309 women), 29,597 low risk primiparous women gave birth in private hospitals in NSW and 79,792 low risk primiparous women gave birth in public hospitals. The rate of obstetric intervention were much higher amongst those who gave birth in private hospitals compared to those who gave birth in public hospitals when all interventions for pre-specified medical reasons were removed. Low risk primiparous women giving birth in private hospitals had higher rates of induction for no medical reason (19% vs 7%), instrumental birth (30% vs 20%), caesarean section (25% vs 16%), epidural (71% vs 35%) and episiotomy (42% vs 23%). Severe perineal trauma (defined as third and fourth degree perineal trauma) was slightly lower in a private hospital in first time mothers (4.7% vs 5.4%) (Table 1).

Amongst the total population of multiparous women (403,429 women), 28,703 low risk multiparous women gave birth in private hospitals and 99,212 low risk multiparous women gave birth in public hospitals The rate of obstetric intervention was significantly higher amongst those who gave birth in private hospitals in NSW compared to those who gave birth in public hospitals when all interventions for specific medical reasons were removed. Low risk multiparous women who gave birth in private hospitals had higher rates of induction for no medical reason (32% vs 10%), instrumental birth (8% vs 3%), epidural (34% vs 10%) and episiotomy (16% vs 5.%) and similar rates of severe perineal trauma (0.9%). The caesarean section rate still remained higher in the private cohort (5.9% vs 4.5%) though this was mostly associated with elective caesarean section (Table 2).

Perinatal characteristics, interventions and outcomes

There was no difference in birth weight between babies born in a private and public hospital. Babies born in a private hospital were significantly more likely to be born at 37, 38, 39 and 40 weeks and less likely to be born at 41 weeks gestation (Figure 1.).

For peer review only - http://bmjopen.bmj.com/site/about/guidelines.xhtml

Babies of primiparous women born in a private hospital were less likely to have an Apgar of <7 at five minutes (AOR 1.34 95% CI 1.77-1.530; p<0.001) or not to be resuscitated (AOR 0.354 95% CI 0.354-0.374; p<0.001). Babies born to low risk primiparous women in a private hospital were no more likely to be admitted to special care and/or neonatal intensive care (AOR 1.03 95% CI 0.984-1.075; p 0.210) and were less likely to have their baby transferred to another hospital (AOR 7.55 95% CI 6.522-8.738; p<0.001). There was no difference in the perinatal mortality rate for babies of primiparous women born in private or public hospitals (AOR 1.49 95% CI 0.926-2.410; p 0.100) (Table 3). Similar outcomes were seen for babies born to multiparous women in private and public hospitals, except there was no difference in the incidence of Apgars of <7 at five minutes (Table 4).

Reason for birth admission of neonate

We examined neonatal morbidity as coded on the neonatal birth admission record and found fewer babies overall had a morbidity record in the private sector compared to the public sector (40% vs 53.9%) due to the added complexity in the public sector. There were however some significant differences noted under the main ICD-10-AM Grouping *Certain conditions originating in the perinatal period (P00-P96)*. Babies born in a private hospital were more likely to have been affected by a forceps or vacuum delivery and were more likely to have trauma to the scalp (8.1% vs 4.1%), intrauterine hypoxia (4.3% vs 2.2%), jaundice (11.7% vs 2.9%), minor cardiac murmurs (0.6% vs 0.3%), conjunctivitis (7.5% vs 2.3%), respiratory conditions (2.4% vs 1.1%), temperature regulation issues (5.2% vs 2.4%) feeding difficulties (9.6% vs 4.3%), carbohydrate metabolism issues (2.2% vs 1.0%), vomiting (1.4% vs 0.7%) and be circumcised during the birth admission (6.7% vs 0.2%). Babies born in a public hospital were more likely to be admitted for observation and evaluation (11.8% vs 9.4%) have prophylactic antibiotics (1.0% vs 0.4%) and be admitted for socioeconomic circumstances (eg. housing, distance, adoption, assumption of care) (1.1% vs 0.1%) (Table 5)

Reason for birth re-admission of neonate up to 28 days of age

For peer review only - http://bmjopen.bmj.com/site/about/guidelines.xhtml

We examined the reasons for transfer or readmission of babies up until 28 days of age and found, that though the numbers are small, more babies born in private hospitals were readmitted compared to babies born in a public hospital (0.95% vs 0.65% (Table 6). Babies born in private hospitals were more likely to be readmitted for infectious diseases (18.1% vs 14.4%), endocrine, nutritional and metabolic disorders (6.4% vs 3%), behavioural disorders (4.8% vs 2.5%), sleep disorders (2.6% vs 0.6%), hypoglycaemia (2% vs 0.9%), birth trauma such as cephalohaematoma (2.0% vs 0.9%); trauma involving the scalp (4.4% vs 2.1%) and excessive crying (12.1% vs 7.8%). Babies born in public hospitals were more likely to be readmitted with respiratory disorders (23.3% vs 8%), injury and poisoning (eg burns) (6.2% vs 2.2%), for observation (6.8% vs 4.9%), antibiotic therapy (3.3% vs 0.7%) and socioeconomic circumstances (housing, distance, adoption, assumption of care (13.5% vs 3.9%).

Combined birth and readmission neonatal morbidity for selected codes

When we combined major birth and readmission morbidities for key selected codes we found that in the first 28 days following birth, babies born in private hospitals were significantly more likely to be admitted for feeding difficulties (9.6% vs 4.4%), circumcision (6.7% vs 0.3%), birth trauma (mostly scalp trauma) (12.2% vs 6.6%), hypoxia (4.2% vs 2.2%), respiratory disorders (3% vs 1.4%) and seep/behavioural issues (0.5% vs 0.2%). Babies born in public hospitals were more likely to be admitted for socioeconomic circumstances such as housing, distance, adoption or assumption of care (1.2% vs 0.2%) and prophylactic antibiotics (1.1% vs 0.4%) (Table 7 & Figure 2).

Discussion

Intervention rates

Despite being an extremely low risk cohort, less than half the primiparous women in this study giving birth in a private hospital had a normal vaginal birth (45% vs 65%); this was 20% lower than in the public cohort. One in five primiparous women giving birth in a private hospital were

For peer review only - http://bmjopen.bmj.com/site/about/guidelines.xhtml

induced and nearly one in two had an episiotomy. For low risk multiparous women giving birth in a private hospital nearly one in three were induced. The trend for higher intervention rates has been reported for low risk women giving birth in the private sector in Australia previously and continues to show an increase [9, 14, 15]. In a recent publication we showed that the rate of caesarean section had increased in both the private and public sector in the past decade in low risk women [9]. It has been argued in a previous publication that these high intervention rates in the private sector led to better perinatal outcomes than in the public sector [2]. This publication received significant criticism in letters to the editor [3, 16, 17] for several methodological flaws, including most significantly the failure to adjust for low birth weight, inadequate ascertainment of congenital abnormalities and failure to look at perinatal morbidity. In this study we included only low risk women, adjusting for maternal age and gestational age differences. We also removed all babies with congenital abnormalities from this data set. We found that the perinatal mortality rate was not statistically different when the populations were matched in this data set for maternal risk.

Neonatal resuscitation and admission to SCN/NICU

We found that babies born in a private hospital were much more likely to experience some form of resuscitation, in particular twice the rate of suctioning at birth. Routine suctioning for infants born with clear and/or meconium stained amniotic fluid is not recommended [18] as it can cause a bradycardia [19] and there is no evidence of benefit. We are unsure why such a high rate of newborn suctioning continues in the private sector. While rates of Apgar scores of ≤7 at five minutes were slightly higher amongst primiparous women who gave birth in public hospital (but not multiparous women), and this has been demonstrated in another recent Australian publication [15], overall the babies were no more likely to be admitted to SCN/NICU compared to babies born in private hospitals.

Neonatal admission and readmission

We found some interesting differences in morbidity however when examining morbidity attached to the birth admission and readmission to

For peer review only - http://bmjopen.bmj.com/site/about/guidelines.xhtml

hospital in the first 28 days for codes that may be associated with the higher rates of obstetric intervention in the private sector and a different sociodemographic profile in the public sector. While increasingly preterm babies >35 weeks/ and or >2.2k and some cases of jaundice may be managed at the bedside in some hospitals, this is less likely to occur in a private hospital. It is more likely to occur in large maternity units.

Birth trauma, in particular injuries to the scalp, were twice as high in the private sector and these are generally associated with instrumental birth, including vacuum extraction [20-22]. With more women (nearly one in three primiparous women) experiencing an instrumental birth in the private cohort and one in five women in the public sector this is not surprising. Birth trauma is associated with a longer hospital stay and increased risk of admission to SCN/NICU as well as higher rates of neonatal morbidity including neurological morbidity (hypotonia, jitteriness, convulsions and hypoxic ischaemic encephalopathy) and jaundice [22].

Jaundice was observed to be higher in the private sector, which may be related to several factors, such as the elective delivery of babies at an earlier gestation, the increased scalp trauma due to the high instrumental birth rate, as discussed above, and potential breastfeeding difficulties due to higher use of epidural analgesia. Jaundice has been associated with birth trauma, in particular delivery by vacuum extraction, and problems with feeding, especially supplementary feeding [22, 23]. Earlier gestational age <39 weeks has also be found to be associated with jaundice, with this decreasing with each week of additional gestation [24]. The gestational age of babies born in private hospitals in this study was significantly lower than in the public sector due to the high rates of non-medical induction of labour and non-medically indicated caesarean section before the onset of labour.

While there have been studies associating difficulties with breastfeeding and higher rates of jaundice, the recent publication from the Universal Screening for Hyperbilirubinemia Study Group found this was a minimal risk factor.

Twice as many babies who were born in a private hospital in this study were admitted or re-admitted with feeding problems compared to babies born in a public hospital. Feeding difficulties are associated with operative birth interventions and being early term [25, 26].

For peer review only - http://bmjopen.bmj.com/site/about/guidelines.xhtml

Breastfeeding outcomes are positively associated with uncomplicated unassisted vaginal birth where the mother and infant remain together and breastfeeding is started within an hour of the birth and following skin-to-skin contact. Interventions during labour and birth can impact on the initiation and duration of breastfeeding. Caesarean section [27-30], instrumental birth [31], epidural anaesthesia and opioid analgesia [32-34] use have all been associated with breastfeeding difficulties. All these birth interventions were higher in the private cohort in this study. In addition early term birth, which is mainly due to induction of labour and elective caesarean section (35, 36) is associated with increased breastfeeding difficulties along with other serious morbidities [37]. Unmedicated newborns are more highly aroused immediately following the birth [38] and able to breastfeed without assistance if given skin to skin contact and freedom from intrusive procedures [39, 40]. Following caesarean section there can be a significantly longer period of time until a mother touches and holds her newborn compared to an unassisted vaginal birth [41].

In a previous paper [6] using national Australian population data we found that among low-risk women who had an unassisted vaginal birth with spontaneous onset of labour and no labour augmentation, the odds of admission to neonatal intensive care or special care nursery were significantly increased when the baby was 37 weeks gestation at the time of birth compared to later gestations. Some claim that during the final weeks of gestation the fetal brain goes through a marked increase in mass and nerve growth (corticoneurogenesis) which may be best left undisturbed [42]. In this study low risk women giving birth in private hospitals in NSW were much more likely to give birth at earlier gestations than their public hospital counterparts for every week up to and including 40 weeks, but they were significantly less likely to deliver at 41 weeks. This may also help to explain why more babies born in a private hospital were readmitted with, respiratory, feeding, jaundice and sleep and behavioural problems. However there is also evidence that there are increased adverse perinatal outcomes for babies born following 41 completed weeks, but we did not examine this population [43].

For peer review only - http://bmjopen.bmj.com/site/about/guidelines.xhtml

Circumcision

Babies born in a private hospital were significantly more likely to be circumcised in the first 28 days of life (around 23 times more likely). This may be due to different information being given in private hospitals about the procedure or easy access to providers who perform the procedure. Circumcision rates are estimated to be between 10-20% in Australia [44] and are decreasing. A recent position statement of the Royal Australian College of Physicians states "that the frequency of diseases modifiable by circumcision, the level of protection offered by circumcision and the complication rates of circumcision do not warrant routine infant circumcision in Australia and New Zealand" [44].

Socioeconomic circumstances

The difference in the socioecononomic status of the women giving birth in public compared to private hospitals appears is demonstrated by the significantly higher rates of public hospital babies with a morbidity attached to the birth admission or readmission in the first 28 days for socioeconomic circumstances, including housing, distance, adoption and assumption of care. This again confirms what is already known that the two populations are very different sociodemographically with greater disadvantage in the public sector.

Limitations

Our study is limited to providing a snapshot of perinatal outcomes in one state in Australia in a defined time period for women who have no indicated risk at birth. However, this study provides useful data following on from our previous paper looking at obstetric intervention in private and public hospitals in NSW providing the reader with a detailed picture of perinatal mortality and morbidity. The advantages of using

For peer review only - http://bmjopen.bmj.com/site/about/guidelines.xhtml

population-based datasets such as the PDC and the linkage to four other population-based databases include the size of the sample and the high level of accuracy of a validated dataset. The limitations are the restricted number of variables that are included and the scarcity of specific information on potential influencing variables. A small number of cases with a low linkage rate (0.3%) were not included and so there is the possibility of missing adverse outcomes. A previous study showed that where stillbirths are excluded due to low linkage these are at lower gestational ages and not term infants as were the focus in this study [45]. Previous validation studies have reported high levels of data accuracy for the majority of diagnoses and procedures conducted during labour and delivery in the state-wide data base [46, 47], although the recording of medical conditions and smoking are overall generally underreported [46, 48]. Having a linked data set provides a much richer picture than we have had previously of the morbidity and mortality associated with birth interventions. While we could not control for obesity due to lack of data, women who have private health insurance have lower rates of obesity and higher socioeconomic status, hence these health disadvantages are most likely over represented in the public women [49]. There are also several other socio-demographic factors we could not control for, such as education and income, that increase risk for the women giving birth in public hospitals. This study can only provide an overview of possible associations between obstetric interventions and neonatal outcomes and does not imply causality, which could be better obtained from prospective cohort studies.

Conclusion

The continual rise in obstetric intervention for low risk women in Australia may be contributing to increased morbidity for healthy women and babies and higher cost of health care. The fact that these procedures which were initially life-saving are now so commonplace and do not appear to be associated with improved rates of perinatal mortality or morbidity demands close review. Early term delivery and instrumental births may be associated with increased morbidity in neonates and this requires urgent attention.

For peer review only - http://bmjopen.bmj.com/site/about/guidelines.xhtml

References

1 Li Z, Zeki R, Hilder L, Sullivan EA. Australia's mothers and babies 2010. Perinatal statistics series no. 27. Cat. no. PER 57. In: Unit ANPEaS, editor. Canberra2012.

2 Robson SJ, Laws P, Sullivan EA. Adverse outcomes of labour in public and private hospitals in Australia: a population based descriptive study. The Medical Journal of Australia 2009;190(9):474-7.

3 Tracy S, Welsh A, Dahlen H, Tracy M. Letter to the Editor re Robson SJ, Laws P, Sullivan EA. Adverse outcomes of labour in public and private hospitals in Australia: a population-based descriptive study. Med J Aust 2009; 190: 474-477. Medical Journal of Australia. 2009;191(10):579-80.

4 Clark E.A.S, Silver RM. Long-term maternal morbidity associated with repeat caesarean delivery. American Journal of Obstetrics and Gynecology. 2011;S2(December).

5 Hyde MJ, Mostyn A, Modi N, Kemp PR. The health implications of birth by caesarean section. Biological Reviews. 2012;87(1):229-43.

6 Tracy S, Tracy M, Sullivan E. Admission of Term Infants to Neonatal Intensive Care: A Population-Based Study. Birth 2007;34(4):301-7.

7 Tracy S, K , Tracy M. Costing the cascade: estimating the cost of increased obstetric intervention in childbirth using population data. British Journal of Obstetrics and Gynaecology. 2003;110:717-24.

8 Allen VM, O'Connell CM, Farrell SA, Baskett TF. Economic implications of method of delivery. Am J Obstet Gynecol 2005;193(1):192-7.

9 Dahlen HG, Tracy S, Tracy M, Bisits A, Brown C, Thornton C. Rates of obstetric intervention among low-risk women giving birth in private and public hospitals in NSW: a population-based descriptive study. BMJ Open. 2012;2:e001723 doi:10.1136/bmjopen-2012-001723.

10 Roberts C, L,, Tracy S, Peat B. Rates of obstetric intervention among private and public patients in Australia: population based descriptive study. British Medical Journal. 2000;312:137-41.

11 Buist R. Letter to the Editor: Private Obstetrics-again. BMJ Open. 2012;2(5):e001723 doi:10.1136/bmjopen-2012-.

12 CHeReL. Centre for Health Record Linkage (CHeReL). Quality Assurance Report 2012. http://wwwcherelorgau/media/24160/qa_report_2012pdf. 2012.

13 Lain SJ, Hadfield RM, Raynes-Greenow CH, Ford JB, Mealing NM, Algert CS, et al. Quality of data in perinatal population health databases: a systematic review. Med Care. 2012;50(4):e7-e20.

For peer review only - http://bmjopen.bmj.com/site/about/guidelines.xhtml

14 Roberts CL, Tracy S, Peat B. Rates for obstetric intervention among private and public patients in Australia: population based descriptive study. British Medical Journal. 2000 2000;321(7254):137-41.

15 Einarsdóttir K, Stock S, Haggar F, Hammond G, Langridge AT, Preen DB, et al. Neonatal complications in public and private patients: a retrospective cohort study. BMJ Open. 2013;Downloaded from bmjopen.bmj.com on November 11, 2013.

16 Evans N, Malcolm G, Gordon A. Letter to the Editor re Adverse outcomes of labour in public and private hospitals in Australia. MJA. 2009;191(10):579.

17 Chambers D. Letter to the Editor re Adverse outcomes of labour in public and private hospitals in Australia. MJA. 2009;191(10):578-9.

18 Wyllie J. Recent changes to UK newborn resuscitation guidelines. Arch Dis Child Fetal Neonatal Ed. 2012;97(1):F4-F7.

19 Kannapiran R, MKennea N. Resuscitation of the newborn. Obstetrics, Gynecology & Reproductive Medicine. 2012;22(4):92-7.

20 Doumouchtsis SK, Arulkumaran S. Head injuries after instrumental vaginal deliveries. Current Opion in Obstetrics and Gynaecology. 2006;18:129-34.

21 Towner D, Castro MA, Wilkens EE, Gilbert WM. Effect of Mode of Delivery in Nulliparous Women on Neonatal Intracranial Injury. The New England Journal of Medicine 1999;341:1709-14.

22 Linder N, Linder I, Fridman E, Kouadio F, Lubin D, Merlob P, et al. Birth trauma – risk factors and short-term neonatal outcome. The journal of Maternal-fetal & Neonatal Medicine. 2013;DOI: 10.3109/14767058.2013.789850.

23 Bertini G, Dani C, Tronchin M, Rubaltelli FF. Is breastfeeding really favouring early neonatal jaundice? Pediatrics. 2006;107(3):e41.

24 Bhutani VK, Stark AR, Lazzeroni LC, Poland R, Gourley GR, Kazmierczak S, et al. Predischarge Screening for Severe Neonatal Hyperbilirubinemia Identifies Infants Who Need Phototherapy. The Journal of Pediatrics. 2013;162(3):477-82.

25 Young PC, Korgenski K, Buchi KF. Early Readmission of Newborns in a Large Health Care System. Pediatrics. 2013;131(5):e1538-e44.

26 Brown A, Jordan S. Impact of birth complications on breastfeeding duration: an internet survey. Journal of Advanced Nursing. 2012;69(4):828-39.

27 Chapman D.J, Perez-Escamilla R. Identification of risk factors for delayed onset oflactation. Journal of the American Dietetic Association. 2003;99:450-4.

For peer review only

For peer review only - http://bmjopen.bmj.com/site/about/guidelines.xhtml

28 Dewey K.G., Nommsen-Rivers L.A., Heinig M.J, Cohen R.J. Risk factors for suboptimal infant breastfeeding behavior, delayed onset of lactation and excess neonatal weight loss. Pediatrics 2003;112(3):607-19.

29 Scott J.A., Binns C.W, Oddy W.H. Predictors of delayed onset of lactation. Maternal and Child Nutrition. 2007;3(3):186-93.

30 Jordan S., Emery S., Watkins A., Evans J.D., Storey M, Morgan G. Associations of drugs routinely given in labour with breastfeeding at 48 hours: analysis of the Cardiff Births Survey. British Journal of Gynecology. 2009;116(12):1622-9.

31 Leung G.M, Lam T.H, Ho L.M. Breast-feeding and its relation to smoking and mode of delivery. Obstetrics and Gynecology 2002;99(5):785-94.

32 Jordan S. Infant feeding and analgesia in labour: the evidence is accumulating. International Breastfeeding Journal Research. 2006;1(25):doi: 10.1186/746-4358-1-25.

33 Henderson J.J., Dickinson J.E., Evans S.F., McDonald S.J, M.J. P. Impact of intrapartum epidural analgesia on breast- feeding duration. Australian and New Zealand Journal of Obstetrics and Gynaecology. 2003;43(5):372–7.

34 Beilin Y., Bodian C., Weiser J., Sabera H., Ittamar A., Feierman D., et al. Effect of labor analgesia with and without fentanyl on infant breastfeeding: a prospective, randomized double-blind study. Anesthesiology. 2005;103(6):1211– 7.

35 Ohnsorg T, Schiff J. Preventing elective induction before 39 weeks. , 93(11), 44-46. Minnesota Medicine. 2010;93(11):44-6.

36 Oshiro BT, Henry E, Wilson J, Branch DW, Varner MW. Decreasing elective deliveries before 39 weeks of gestation in an integrated health care system. Obstetrics & Gynecology. 2009;113:804-11.

37 Reddy UM, Ko CW, Willinger M. “Early term” births (37-38 weeks) are associated with increased mortality. American Journal of Obstetrics & Gynecology. 2006;195(S202).

38 McLaughlin FJ, O’Connor S, Deni R. Infant state and behavior during the first postpartum hour. The Psychological Record 1981;31:455–8.

39 Widstrom A-M, Ransjo-Arvidson AB, Christensson K, Matthiesen A- S, Winberg J, Uvnas-Moberg K. Gastric suction in healthy newborn infants. Effects on circulation and developing feeding behaviour. Acta Paediatr. 1987;76:566–72.

40 Righard L. How do newborns find their mother’s breast? . Birth. 1995;22:174–5.

For peer review only - http://bmjopen.bmj.com/site/about/guidelines.xhtml

41 Fisher J, Astbury J, Smith A. Adverse psychological impact of opera- tive obstetric interventions: a prospective study. Aust NZ J Psychiatry 1997;31:728–38.

42 Adams-Chapman I. Insults to the developing brain and impact on neurode- velopmental outcome. Journal of Communication Disorders. Journal of Communication Disorders. 2009;42:256-62.

43. Gülmezoglu AM, Crowther CA, Middleton P, Heatley E. Induction of labour for improving birth outcomes for women at or beyond term. *CochraneDatabase of Systematic Reviews* 2012;Issue 6. Art.No.:CD004945. DOI: 10.1002/14651858.CD004945.pub3.

44 The Royal Australian College of Physicians. Circumcision of infant males. In: Physicians TRACo, editor. Sydney: The Royal Australasian College of Physicians; 2010.

45 Bentley JP, Ford JB, Taylor LK, Irvine KA, Roberts CL. Investigating linkage rates among probabilistic linked births and hospital records. BMC Medical Research Methodology. 12:149, 2012.

46 Taylor L, Travis S, Pym M, Olive E, Henderson-Smart D. How useful are hospital morbidity data for monitoring conditions occurring in the perinatal period? Australian & New Zealand Journal of Obstetrics & Gynaecology. 2005;45:36 –41.

47 Roberts C, Bell J, Ford J, Morris J. Monitoring the quality of maternity care: how well are labour and delivery events reported in population health data? . Paediatirc and Perinatal Epidemiology. 2008;23:144-52.

48 Thornton C, Makris A, Ogle R, Hennessy A. Generic obstetric database systems are unreliable for reporting the Hypertensive Disorders of Pregnancy. Australian and New Zealand Journal Obstetrics and Gynaecology. 2004;44(505-509).

49 Finkelstein A, Fiebelkorn IC, Wang G. National medical Spendig attributable to overweight and obesity: How much and who's paying. Health Affairs, 2003 Project HOPE–The People-to-People Health Foundation, Inc., 219-226. 2003.

Table 1. Maternal characteristics, interventions and outcomes for low risk primiparous women in NSW (2000-2008)

For peer review only - http://bmjopen.bmj.com/site/about/guidelines.xhtml

For peer review only

Low risk Primiparous women	Private Hospital (n=29597)	Public Hospital (n=79792)
**Maternal age (years)**		
20-24	6.2%	28.9%
25-29	39.9%	40.9%
30-34	53.9%	30.2%
Weeks gestation at delivery		
37	4.5%	4.3%
38	15.4%	11.8%
39	27.8%	25.0%
40	43.2%	39.7%
41	9.1%	19.2%
**Type of labour**		
Spontaneous	71.9%	89.9%
Induced	19.2%	7.1%
No labour	8.9%	3.0%
**Delivery**		
Normal vaginal	44.9%	64.8%
Forceps	11.5%	6.7%
Vacuum	18.9%	12.9%

For peer review only

Total caesarean section	24.7%	15.6%
Caesarean section (after labour)	15.9%	12.6%
Caesarean section before the onset of labour	8.8%	3.0%
**Epidural**	70.8%	35.4%
**Episiotomy**	42.4%	23.3%
**Severe perineal trauma**	4.7%	5.4%

Table 2. Maternal characteristics, interventions and outcomes for low risk multiparous women in NSW (2000-2008)

**Low risk Multiparous women**	**Private Hospital (n=28703)**	**Public Hospital (n=99212)**
**Maternal age (years)**		
20-24	2.1%	16.8%
25-29	25.8%	38.8%
30-34	72.1%	44.4%
Weeks gestation at delivery		
37	4.1%	4.0%

For peer review only - http://bmjopen.bmj.com/site/about/guidelines.xhtml

38	18.7%	13.0%
39	31.6%	26.9%
40	40.2%	40.8%
41	5.4%	15.3%
**Type of labour**		
Spontaneous	64.0%	87.4%
Induced	32.1%	10.1%
No labour	3.9%	2.5%
**Delivery**		
Normal vaginal	86.1%	92.7%
Forceps	1.9%	0.7%
Vacuum	6.1%	2.1%
Total caesarean section	5.9%	4.5%
Caesarean section after labour	2.0%	2.0%
Caesarean section before the onset of labour	3.9%	2.5%#
**Epidural**	34.4%	9.5%
**Episiotomy**	16.2%	5.1%

For peer review only - http://bmjopen.bmj.com/site/about/guidelines.xhtml

**Severe perineal trauma**	0.9%	0.9%

Figure 1. Comparison of gestation at delivery between all women stratified by hospital type

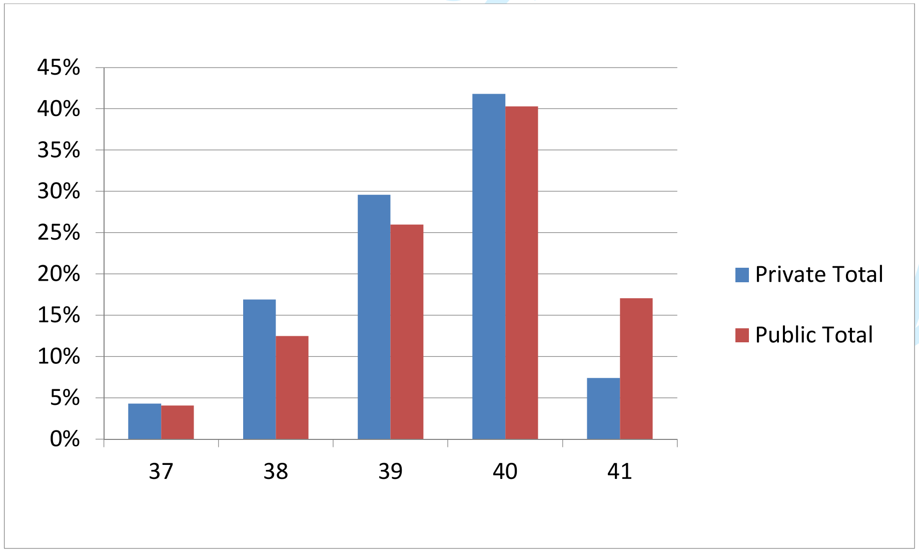

For peer review only - http://bmjopen.bmj.com/site/about/guidelines.xhtml

Table 3. Perinatal outcomes adjusted for maternal age and gestation at birth for primiparous women

	Private	Public			
	(n=29597)	(n=79791)	OR**	AOR**	p
Apgar<7 at 5 minutes	296 (1.0%)	1037 (1.3%)	1.36 (1.198-1.542)	1.34(1.177-1.530)	<0.001
Any resuscitation*	18498 (62.5%)	30560 (38.3%)	0.372 (0.363-0.382)	0.364 (0.354-0.374)	<0.001
Admitted to SCN and/or NICU	3078 (10.4%)	8139 (10.2%)	1.00 (0.964-1.049)	1.03 (0.984-1.075)	0.210
Transferred	178 (0.6%)	3351 (4.2%)	7.30 (6.289-8.403)	7.55 (6.522-8.738)	<0.001
Total Perinatal Mortality	22 (0.74/1000)	85 (1.06/1000)	1.40 (0.930-2.006)	1.49 (0.926-2.410)	0.100

*Any resuscitation includes: Suction, oxygen, IPPR by bag and mask, Intubation and IPPR, external cardiac massage and ventilation and other

** Private hospital is the reference category

For peer review only

Table 4. Perinatal outcomes adjusted for maternal age and gestation at birth for multiparous women

	Private	Public			
	(n=28703)	(n=99212)	OR**	AOR**	p
Apgar<7 at 5 minutes	67 (0.2%)	149 (0.2%)	1.56 (0.980-2.098)	1.42 (0.96-2.21)	0.562
Any resuscitation*	14820 (51.6%)	29867 (30.1%)	0.404 (0.393-0.415)	0.399 (0.388-0.460)	<0.001
Admitted to SCN and/or NICU	1775 (6.2%)	5870 (5.9%)	0.957 (0.906-1.011)	1.027 (0.970-1.087)	0.363
Transferred	232 (0.8%)	4375 (4.4%)	5.661 (4.958-6.465)	6.516 (5.699-7.450)	<0.001
Total Perinatal Mortality	17 (0.59/1000)	76 (0.77/1000)	1.294 (0.765-2.189)	1.294 (0.750-2.232)	0.355

*Any resuscitation includes: Suction, oxygen, IPPR by bag and mask, Intubation and IPPR, external cardiac massage and ventilation and other

** Private hospital is the reference category

For peer review only - http://bmjopen.bmj.com/site/about/guidelines.xhtml

Table 5. Morbidity associated with birth admission coded on neonatal birth admission record

		Private		Public		p
**Total neonates with morbidity codes**		**23330**	**40.0%**	**96508**	**53.9%**	<0.001
**ICD-10-AM Grouping**						
**Certain conditions originating in the perinatal period (P00-P96)**						
	Fetus and newborn affected by maternal infectious and parasitic diseases	41	0.2%	989	1.0%	<0.001
	Fetus and newborn affected by forceps delivery	473	2.0%	1108	1.1%	<0.001
	Fetus and newborn affected by delivery by vacuum extractor	511	2.2%	1509	1.6%	<0.001
	**Birth trauma (all body systems)**	**2948**	**12.6%**	**6447**	**6.7%**	<0.001
	- specifically to scalp (included in above total)	1880	8.1%	3965	4.1%	<0.001
	Intrauterine hypoxia	993	4.3%	2170	2.2%	<0.001
	Other specified respiratory conditions of newborn	562	2.4%	1015	1.1%	<0.001
	Benign and innocent cardiac murmurs in newborn	139	0.6%	303	0.3%	<0.001
	Neonatal conjunctivitis specific to the perinatal period	1740	7.5%	2267	2.3%	<0.001
	**Jaundice related conditions**	**2728**	**11.7%**	**5166**	**2.9%**	<0.001
	Transitory disorders of carbohydrate metabolism specific to fetus and newborn	502	2.2%	942	1.0%	<0.001
	Temperature regulation	1214	5.2%	2275	2.4%	<0.001
	Vomiting in newborn	320	1.4%	693	0.7%	<0.001
	Feeding difficulties	2231	9.6%	4157	4.3%	<0.001
**factors influencing health status and contact with health services**						

For peer review only - http://bmjopen.bmj.com/site/about/guidelines.xhtml

**(Z00-Z99)**						
	Routine and ritual circumcision	1552	6.7%	187	0.2%	<0.001
	Observation and evaluation of newborn	2187	9.4%	11372	11.8%	<0.001
	Prophylactic chemotherapy (antibiotics)	93	0.4%	935	1.0%	<0.001
	Socioeconomic circumstances (housing, distance, adoption, assumption of care)	32	0.1%	1020	1.1%	<0.001
		3184	13.7%	49988	51.8%	

Table 6. Morbidity associated with readmission of the baby <28 days of age

		Private		Public		p
**Total admissions**		**689**		**1507**		
**Total neonates**		**552 (0.95%)**		**1155 (0.65%)**		<0.001
**ICD-10-AM Grouping**						
**Certain infectious and parasitic diseases**		**121**	**18.1%**	**217**	**14.4%**	<0.001
**Endocrine nutritional and metabolic diseases**		**30**	**4.4%**	**38**	**2.5%**	<0.001
	Volume depletion	18	2.6%	12	0.8%	<0.001
**Mental and behavioural disorders**		**33**	**4.8%**	**30**	**2.0%**	<0.001
	Non-organic hypersomnia	17	2.5%	19	1.3%	<0.001

For peer review only - http://bmjopen.bmj.com/site/about/guidelines.xhtml

**Diseases of the nervous system**		**34**	**4.9%**	**38**	**2.5%**	<0.001
	Disorders of the sleep wake schedule	18	2.6%	9	0.6%	<0.001
**Diseases of the ear and mastoid process**		**19**	**2.8%**	**23**	**1.5%**	<0.001
**Diseases of the respiratory system**		**155**	**8.0%**	**351**	**23.3%**	<0.001
	Acute obstructive laryngitis (croup)	6	0.9%	16	1.1%	=0.76
	Acute upper respiratory infection unspecified	16	2.3%	52	3.5%	0.84
	Pneumonia	6	0.9%	19	1.3%	=0.86
	Acute bronchiolitis	70	10.2%	175	11.6%	=0.17
	Unspecified acute lower respiratory tract infection	3	0.4%	12	0.8%	0.52
**Diseases of the digestive system**		**53**	**7.7%**	**92**	**6.1%**	<0.001
	Gastro-oesophageal reflux disease	26	3.8%	37	2.5%	0.01
**Certain conditions originating in the perinatal period**		**474**	**68.8%**	**1011**	**67.1%**	<0.001
	Cephalohaematoma due to birth trauma	14	2.0%	13	0.9%	<0.01
	Total birth trauma to scalp	30	4.4%	32	2.1%	<0.001
	Intrauterine hypoxia	4	0.6%	25	1.7%	0.43
	Other neonatal hypoglycaemia	14	2.0%	14	0.9%	<0.01
	Other transitory neonatal electrolyte and metabolic disturbances	5	0.7%	21	1.4%	=0.69

For peer review only - http://bmjopen.bmj.com/site/about/guidelines.xhtml

	Fever of newborn	5	0.7%	18	1.2%	0.92
	Feeding problems of newborn	40	5.8%	100	6.6%	=0.31
	**Neonatal jaundice**	**193**	**28%**	**90**	**6%**	<0.001
**Symptoms, signs and abnormal findings not elsewhere classified**		**198**	**28.7%**	**340**	**22.6%**	<0.001
	Fever	19	2.8%	27	1.8%	=0.02
	Feeding difficulties and mismanagement	25	3.6%	37	2.5%	<0.01
	Excessive crying	83	12.1%	117	7.8%	<0.001
**injury, poisoning and certain other consequences of external causes**		**15**	**2.2%**	**94**	**6.2%**	<0.001
	Burns	0	0.0%	30	2.0%	<0.001
**factors influencing health status and contact with health services**		**192**	**28.9%**	**691**	**45.9%**	<0.001
	Observation	34	4.9%	102	6.8%	=0.92
	Other prophylactic antibiotic therapy	5	0.7%	49	3.3%	<0.01
	Routine and ritual circumcision	68	9.9%	119	7.9%	<0.001
	Attention to surgical dressings and sutures	0	0.0%	28	1.9%	
	Socio-economic circumstances (housing, distance, adoption, assumption of care)	22	3.9%	156	13.5%	<0.001

For peer review only - http://bmjopen.bmj.com/site/about/guidelines.xhtml

Table 7. Combined birth and readmission neonatal morbidity for selected codes

	Private	Public	P*
Total feeding difficulties	2314 (9.6%)	4306 (4.4%)	<0.0001
Total circumcision	1620 (6.7%)	306 (0.3%)	<0.0001
Total socio-economic circumstances	54 (0.2%)	1176 (1.2%)	<0.0001
Total birth trauma	2922 (12.2%)	6492 (6.6%)	<0.0001
Total hypoxia	997 (4.2%)	2195 (2.2%)	<0.0001
Total jaundice	2818 (11.8%)	5359 (5.5%)	<0.0001
Total respiratory	717 (3.0%)	1366 (1.4%)	<0.0001
Total sleep/behavioural issues	118 (0.5%)	145 (0.2%)	<0.0001
Prophylactic antibiotics	98 (0.4%)	982 (1.1%)	<0.001

*chi-square

Figure 2. Birth trauma as a percentage of all births in Private and Public Hospitals

For peer review only

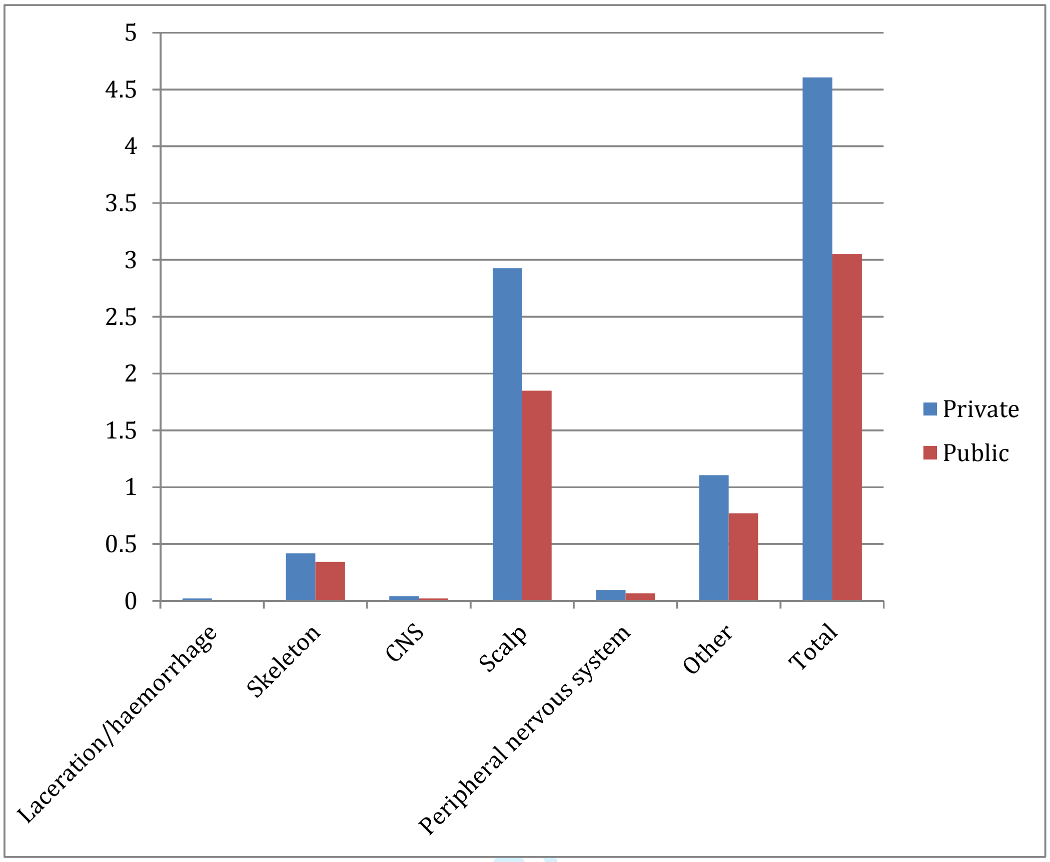

CNS-Central Nervous System, PNS- Peripheral Nervous System

For peer review only - http://bmjopen.bmj.com/site/about/guidelines.xhtml

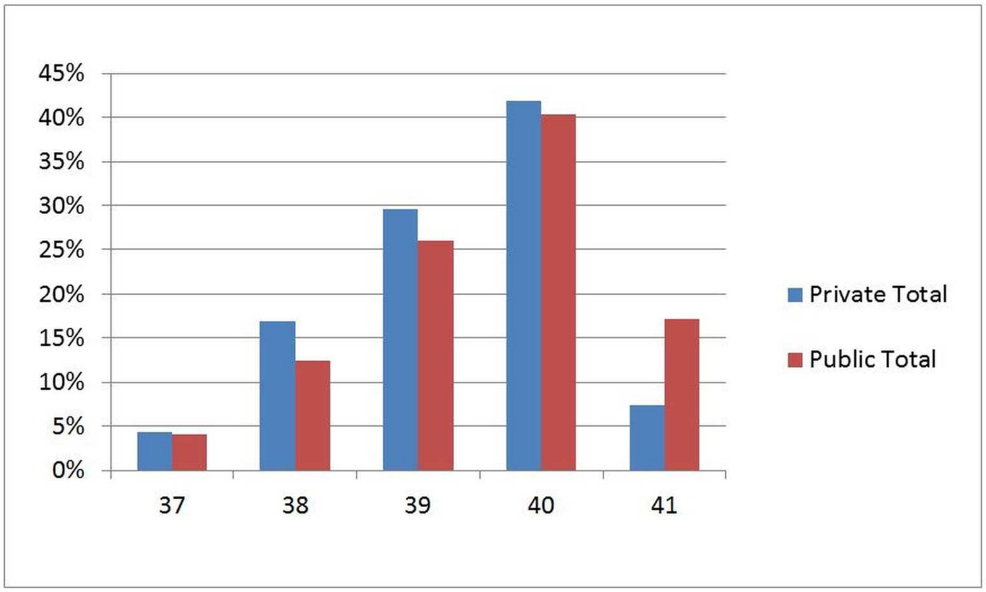

150x90mm (300 x 300 DPI)

review only

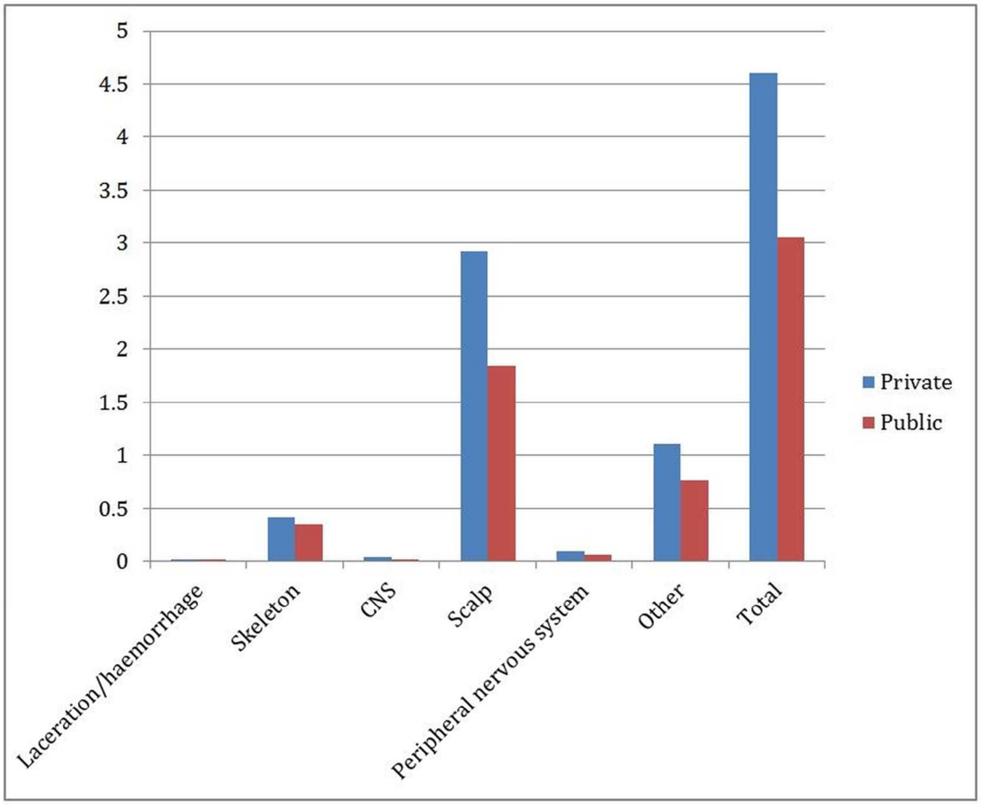

110x90mm (300 x 300 DPI)

For peer review only

Rates of obstetric intervention and associated perinatal mortality and morbidity amongst low risk women giving birth in private and public hospitals in NSW (2000-2008): A linked data population based cohort study.

Journal:	*BMJ Open*
Manuscript ID:	bmjopen-2013-004551.R2
Article Type:	Research
Date Submitted by the Author:	02-Apr-2014
Complete List of Authors:	Dahlen, Hannah; University of Western Sydney, School of Nursing and Midwifery<br>Tracy, Sally; University of Sydney, Nursing and Midwifery<br>Tracy, Mark; University of Sydney, Medicine<br>Bisits, Andrew; Royal Hospital for Women, Royal Hopsital for Women<br>Brown, Chris; University of Sydney, NHMRC Clinical Trials Centre<br>Thornton, Charlene; University of Western Sydney, School of Nursing and Midwifery
**Primary Subject Heading**:	Obstetrics and gynaecology
Secondary Subject Heading:	Epidemiology, Health policy, Paediatrics
Keywords:	Health policy < HEALTH SERVICES ADMINISTRATION & MANAGEMENT, NEONATOLOGY, OBSTETRICS

For peer review only

Rates of obstetric intervention and associated perinatal mortality and morbidity amongst low risk women giving birth in private and public hospitals in NSW (2000-2008): A linked data population based cohort study.

Hannah G. Dahlen RN, RM, BN (Hons), MCommN, PhD, FACM

Associate Professor of Midwifery

School of Nursing and Midwifery

Family and Community Health Group

Locked Bag 1797

Penrith South DC

NSW

Australia

Ph: +61 2 96859118

h.dahlen@uws.edu.au

Sally Tracy

Professor Midwifery

Professor of Midwifery

University of Sydney,

Royal Hospital for Women, Sydney

Tel: 0420277106

For peer review only

For peer review only - http://bmjopen.bmj.com/site/about/guidelines.xhtml

Mark Tracy

Senior Newborn Intensive Care Specialist and HOD

CNBC Westmead Hospital

Senior Clinical Lecturer Paediatrics and Child Health

University of Sydney

Tel (02) 9845 6568

Andrew Bisits Mb bs(unsw)franzcog ,mmed stat ,dip clin epi

Acting Medical Co Director of Maternity, Lead Clinician Birthing Suite

Royal Hospital for Women

Conjoint Associate Professor School of Women and Children's Health University of NSW

Randwick NSW 2033

0419418492

Chris Brown, BSc, MBiostat

Biostatistician

NHMRC Clinical Trials Centre

Locked Bag 77

Camperdown NSW 1450

For peer review only

For peer review only - http://bmjopen.bmj.com/site/about/guidelines.xhtml

Charlene Thornton BN, Grad.Dip.Mid, M Sc Med (Research), M Sc Med (Clin Epi), PhD

Post Doctoral Research Fellow

School of Nursing and Midwifery

Family and Community Health Group

Locked Bag 1797

Penrith South DC

NSW

Australia

Keywords: low risk primiparous, low risk multiparous , induction, vaginal birth, obstetric care, caesarean, birth trauma

Word count 4596

Ethical approval was obtained from the NSW Population and Health Services Research Ethics Committee, Protocol No.2010/12/291.

Ethical approval was obtained from the NSW Population and Health Services Research Ethics Committee, Protocol No.2010/12/291.

Ni funds allocated

Nil extra data

For peer review only - http://bmjopen.bmj.com/site/about/guidelines.xhtml

Rates of obstetric intervention and associated perinatal mortality and morbidity amongst low risk women giving birth in private and public hospitals in NSW (2000-2008): A linked data population based cohort study.

Abstract

Objectives: To examine the rates of obstetric intervention and associated perinatal mortality and morbidity in the first 28 days amongst low risk women giving birth in private and public hospitals in NSW (2000-2008).

Design: Linked data population based retrospective cohort study involving five data sets.

Setting: New South Wales, Australia.

Participants: 691,738 women giving birth to a singleton baby during the period 2000 to 2008.

Main outcome measures: Rates of neonatal resuscitation, perinatal mortality, neonatal admission following birth and readmission to hospital in the first 28 days of life in public and private obstetric units.

Results: Rates of obstetric intervention amongst low risk women were higher in private hospitals, with primiparous women 20% less likely to have a normal vaginal birth compared to the public sector. Neonates born in private hospitals were more likely to be less than 40 weeks; more likely to have some form of resuscitation; less likely to have an Apgar <7 at five minutes (if a first baby). Neonates born in private hospitals to low risk mothers were more likely to have a morbidity attached to the birth admission and to be readmitted to hospital in the first 28 days for birth trauma (5% vs 3.6%); hypoxia (1.7% vs 1.2%) ; jaundice (4.8% vs 3%); feeding difficulties (4% vs 2.4%) ; sleep /behavioural issues (0.2% vs 0.1%); respiratory conditions (1.2% vs 0.8%) and circumcision (5.6 vs 0.3%) but they were less likely to be admitted for prophylactic antibiotics (0.2% vs 0.6%) and for socioeconomic circumstances (0.1% vs 0.7%). Rates of perinatal mortality were not statistically different between the two groups.

For peer review only - http://bmjopen.bmj.com/site/about/guidelines.xhtml

Conclusion:

High rates of obstetric intervention amongst low risk women in private hospitals appear to be associated with higher rates of morbidity seen in the neonate and no evidence of a reduction in perinatal mortality.

Keywords: low risk primiparous, low risk multiparous , induction, vaginal birth, obstetric care, caesarean, birth trauma

For peer review only

Article focus:

For peer review only - http://bmjopen.bmj.com/site/about/guidelines.xhtml

- To examine the rates of obstetric intervention and associated perinatal mortality and morbidity in the first 28 days amongst low risk women giving birth in private and public hospitals in NSW (2000-2008) using linked data.
- Rates of neonatal resuscitation, perinatal mortality, neonatal admission following birth and readmission to hospital in the first 28 days of life amongst those infants born in public and private obstetric units.

Key messages

- Babies born to low risk mothers in private hospitals are more likely to require some resuscitation compared to those born in public hospitals.
- For babies born in private hospitals to low risk mothers there are higher rates of morbidity such as birth trauma, hypoxia, jaundice, feeding difficulties, sleep/behavioural issues, respiratory conditions and circumcision. For babies born in public hospitals there are higher rates of antibiotic use and admission for socio-economic reasons (eg housing, distance, adoption and assumption of care)
- Rates of perinatal mortality were comparable between babies born to low risk women giving birth in private and public hospitals

Strengths and limitations of this study

- The strength of this study lies in the large sample size of linked birth data admissions associated with these births.
- The use of data from five population based datasets which have been linked to enhance validity and ascertainment
- Limitations are the restricted number of variables that are included and the scarcity of specific information on potential confounders.

For peer review only - http://bmjopen.bmj.com/site/about/guidelines.xhtml

For peer review only

- Body mass index and key sociodemographic risk factors could not be controlled for and this would have added risk to women giving birth in public hospitals.

Introduction

In Australia, the national statistics reveal that 29% (n =83,573) of women who gave birth in hospital gave birth in private hospitals directly under private obstetric care [1}. The remaining 71% (n=204,399) of women gave birth in public hospitals in Australia. Women who are privately insured have been reported to have better maternal and perinatal outcomes compared to women who give birth in public hospitals as public patients [2]; but it has been argued that these women tend to be less socioeconomically disadvantaged and healthier [3] and therefore might be expected to have better outcomes. Arguments about the impact of private status on health outcomes are in reality complex.

What is not disputed are the much higher rates of obstetric intervention that occur in private hospitals in Australia. At a national level, the intervention rates in childbirth, such as caesarean section, are significantly higher in the private sector (43.1% vs 28.4%) and the rates of normal vaginal birth significantly lower (42.7% vs 61%) [1]. Despite the rising intervention rates over the past decade, the perinatal mortality rate overall has not shown a corresponding decline. There is also growing concern that the short and long term morbidity associated with major obstetric interventions, such as caesarean, may not be insignificant for the mother [4] and the baby [5, 6]. The cost to the tax payer of the rising intervention in childbirth is also significant [7, 8].

A recent study in New South Wales, Australia [9] found among 293,840 low-risk women, rates of obstetric intervention were highest in private hospitals and lowest in public hospitals. Low-risk primiparous women giving birth in a private hospital compared to a public hospital had higher rates of induction (31% vs 23%); instrumental birth (29% vs 18%); caesarean section (27% vs 18%), epidural (53% vs 32%), episiotomy (28% vs 12%) and lower normal vaginal birth rates (44% vs 64%). Low-risk multiparous women had higher rates

For peer review only - http://bmjopen.bmj.com/site/about/guidelines.xhtml

of instrumental birth (7% vs 3%), caesarean section (27% vs 16%), epidural (35% vs 12%), episiotomy (8% vs 2%) and lower normal vaginal birth rates (66% vs 81%). Following a comparison with data from the decade previously [10], these interventions were found to have increased by 5% for women in public hospitals and by over 10% for women in private hospitals [9]. Among low-risk primiparous women giving birth in private hospitals, 15 per 100 women had a vaginal birth with no obstetric intervention compared to 35 per 100 women giving birth in a public hospital [9]. Concern was expressed that perinatal mortality and morbidity was not reported in that paper [11]. In this study we aim to address this through examining the rates of obstetric intervention and associated perinatal mortality and morbidity attached to the birth admission and readmission to hospital in the first 28 days of life for low risk women giving birth in private and public hospitals in NSW (2000-2008).

Methods

Data Sources

The New South Wales Centre for Health Record Linkage conducted linkage of several datasets via the Health Record Linkage (CHeReL). Pregnancy and birth data for the time period July 1st 2000 till June 2nd 2008 of all singleton births were provided by New South Wales (NSW), Ministry of Health as recorded in the NSW Midwives Data Collection (MDC), now the Perinatal Data Collection (PDC). This population based surveillance system contains maternal and infant data on all births of greater than 400 grams birth weight and/or 20 completed weeks gestation. Hospitals are coded either as private or public in the data set. However, the data identifying women who received care in public hospitals under private accommodation status is no longer collected as it had been in the years 1996-97 and for this reason patients who are under private obstetric care in public hospitals are not able to be differentiated from their public counterparts, so for this study we analysed the data by hospital (private/public). A previous study published in 2000 [10] showed that there was a moderating factor on intervention rates when women with private insurance status gave birth in a public hospital, leading to lower intervention rates than when they gave birth in private hospitals.

For peer review only - http://bmjopen.bmj.com/site/about/guidelines.xhtml

The NSW PDC contains statistics on all births in New South Wales - which amounts to one third of all births which occur in Australia annually. Data is provided for maternal age, maternal hypertension, maternal diabetes, parity, private/public patient status, fetal presentation, onset of labour, gestation at birth, delivery type, Apgar scores and admission to neonatal intensive care and resuscitation details for the neonate. This dataset (NSW PDC) was linked to the Admitted Patient Data Collection (APDC) for the time period July 1st 2000 – June 30th 2008. The APDC records all admitted patient services provided by NSW Public Hospitals, Public Psychiatric Hospitals, Public Multi-Purpose Services, Private Hospitals, and Private Day Procedures Centres. The APDC provided additional information, such as data on maternal medical conditions, which was used to exclude further maternal cases and was used to calculate admission and readmission details for neonates. Further linkage occurred to the NSW Registry of Births, Deaths and Marriages (RBDM) and the Australian Bureau of Statistics Death Data, which provided mortality data. The NSW Register of congenital conditions provided cases of congenital conditions, as did the coding in the APDC. Any neonate (and mother pair) with a recorded congenital condition (ICD-10-AM codes Q0.0-Q99.9) on either dataset was removed from the dataset due to their high-risk status. Probabilistic data linkage techniques were utilised for data linkage and de-identified datasets were provided for analysis. Probabilistic record linkage software assigns a 'linkage weight' to pairs of records. For example, records that match perfectly or nearly perfectly on first name, surname, date of birth and address have a high linkage weight, and records that match only on date of birth have a low linkage weight. If the linkage weight is high it is likely that the records truly match, and if the linkage weight is low it is likely that the records are not truly a match. This technique has been shown to have a false positive rate of 0.3% of records [12].

Gestation is recorded at birth and is also recorded in the database according to the woman's menstrual history, usually combined with a routine scan at 12-13 weeks.

Admission to neonatal intensive care refers to admission to special care nursery (SCN) or neonatal intensive care unit (NICU).

For peer review only - http://bmjopen.bmj.com/site/about/guidelines.xhtml

Any resuscitation includes suction of the mouth or nostrils at birth; oxygen administered by mask; intermittent positive pressure respiration (IPPR) by bag and mask or by intubation; external cardiac massage and ventilation.

Ethical approval was obtained from the NSW Population and Health Services Research Ethics Committee, Protocol No.2010/12/291.

Subjects

We classified the low risk primipara as a first time mother aged 20-34 years, who had no pre-existing or pregnancy related hypertension or diabetes, was a non-smoker, and gave birth at 37-41 completed weeks gestation to a singleton baby in a cephalic presentation within the 10th and 90th centiles for gestation and birth weight. The low risk multipara was a woman having her second or subsequent baby aged 20-34 years, who had no pre-existing or pregnancy related hypertension or diabetes, was a non-smoker, gave birth at 37-41 completed weeks gestation to a singleton baby in a cephalic presentation within the 10th and 90th centiles for gestation and birth weight. We excluded women with a previous caesarean section or who were induced for a medical indication, or who underwent a caesarean section for a pre-existing medical indication or gave birth without a trained birth attendant (born before arrival). If a caesarean section was undertaken during labour however for non-reassuring heart rate, dystocia etc these women were included in the study. These characteristics were defined firstly from the PDC with additional medical conditions identified in the APDC being used to exclude cases.

Outcomes

Any neonatal admission including the ICD-10-AM codes Z37.0 (single live birth), Z37.1 (single stillbirth) or Z38.0 (singleton born in hospital) was deemed the birth admission and any ICD-10-AM codes referring to conditions which arise in the perinatal period (P00-P96) and those referring to factors influencing health status and contact with health services (Z00-Z99) which were included in this

For peer review only - http://bmjopen.bmj.com/site/about/guidelines.xhtml

admission were deemed morbidities associated with the birth admission. Any other admission following this discharge from the initial birth admission to home or another hospital was deemed a readmission and included transfers to a hospital other than that where the birth occurred. When examining readmission data, all ICD-10-AM codes recorded were reviewed and those where ≥10 events occurred in either private or public hospitals were marked for analysis. Events were grouped in body systems where appropriate or under headings such as infection for ease of analysis and interpretation.

Morbidity was recorded with the birth admission and rates of events were calculated using the number of babies who had any morbidity recorded with their birth.

Stillbirth and neonatal deaths were calculated from multiple sources but were limited to those that occurred within 28 days of birth and they were only counted once. Death may have been detected on any one of the following four datasets. The PDC 'Discharge status' variable or admissions in the APDC where the case mode separation was coded as 'Died' or the NSW RBDM or ABS Death Data where a death had been recorded. The maternal admission data for any admission that occurred during the pregnancy, as well as the birth admission for all cases of stillbirth or neonatal death were examined to determine any maternal medical or pregnancy related condition. This methodology of utilising multiple data sources to identify cases has been shown by Lain et al (2012) to be the most reliable way to increase ascertainment of cases [13].

Obstetric intervention was defined to include induction, epidural use, episiotomy, instrumental delivery (requiring the use of forceps or vacuum) and delivery via caesarean section.

Data analysis

The cohort was divided into primiparous and multiparous women for the primary analysis of birth outcomes. When examining neonatal status at birth mortality odds ratios were calculated using logistic regression with and without adjustment for age and

For peer review only - http://bmjopen.bmj.com/site/about/guidelines.xhtml

gestation. For neonatal morbidity at birth and readmission, chi-square statistics were calculated for observed events. The number of babies born in a public or a private hospital were used as the denominator when calculating the percentage of babies born with a morbidity code attached to their birth record or the number of babies readmitted with a designated morbidity code. This methodology provides for comparison between place of birth taking into consideration the fact that up to 55 morbidity codes can be attached to any one birth or readmission record. Taking into account the size of the cohort and the number of analyses undertaken, results were considered significant at the level $p<0.01$. Analysis was undertaken with IBM SPSS v.20®

Results

Maternal characteristics, interventions and outcomes

The PDC dataset for the time period July 1st 2000 to 2nd June 2008 contained the antenatal, birth and postnatal details on 691 738 births. The APDC for the time period July 1st 2000 to 30th June 2008 contained >1.1 million admissions for the neonates/children of these women.

From the total population of primiparous women (288,309 women), 29,597 low risk primiparous women gave birth in private hospitals in NSW and 79,792 low risk primiparous women gave birth in public hospitals. The rates of obstetric intervention were much higher amongst those who gave birth in private hospitals compared to those who gave birth in public hospitals when all interventions for pre-specified medical reasons were removed. Low risk primiparous women giving birth in private hospitals had higher rates of induction for non-medical reasons (19% vs 7%), instrumental birth (30% vs 20%), caesarean section (25% vs 16%), epidural (71% vs 35%) and episiotomy (42% vs 23%). Severe perineal trauma (defined as third and fourth degree perineal trauma) was lower in a private hospital in first time mothers (4.7% vs 5.4%) (Table 1).

For peer review only - http://bmjopen.bmj.com/site/about/guidelines.xhtml

Amongst the total population of multiparous women (403,429 women), 28,703 low risk multiparous women gave birth in private hospitals and 99,212 low risk multiparous women gave birth in public hospitals The rate of obstetric intervention was significantly higher amongst those who gave birth in private hospitals in NSW compared to those who gave birth in public hospitals when all interventions for specific medical reasons were removed. Low risk multiparous women who gave birth in private hospitals had higher rates of induction for non- medical reasons (32% vs 10%), instrumental birth (8% vs 3%), epidural (34% vs 10%) and episiotomy (16% vs 5.%) and similar rates of severe perineal trauma (0.9%). The caesarean section rate still remained higher in the private cohort (5.9% vs 4.5%) though this was mostly associated with elective caesarean section (Table 2).

Perinatal characteristics, interventions and outcomes

There was no difference in birth weight between babies born in a private and public hospital. Babies born in a private hospital were more likely to be born at 37, 38, 39 and 40 weeks and less likely to be born at 41 weeks gestation (Figure 1.).

Babies of primiparous women born in a private hospital were less likely to have an Apgar of <7 at five minutes (AOR 1.34 95% CI 1.77-1.53; p<0.001) or not to be resuscitated (AOR 0.36 95% CI 0.35-0.37; p<0.001). Babies born to low risk primiparous women in a private hospital were no more likely to be admitted to special care and/or neonatal intensive care (AOR 1.03 95% CI 0.98-1.08; p 0.210) and were less likely to have their baby transferred to another hospital (AOR 7.55 95% CI 6.52-8.74; p<0.001). There was no difference in the perinatal mortality rate for babies of primiparous women born in private or public hospitals (AOR 1.49 95% CI 0.93-2.41; p 0.10) (Table 3). Similar outcomes were seen for babies born to multiparous women in private and public hospitals however there was no difference in the incidence of Apgars of <7 at five minutes (Table 4).

Reason for birth admission of neonate

For peer review only - http://bmjopen.bmj.com/site/about/guidelines.xhtml

We examined neonatal morbidity as coded on the neonatal birth admission record and found fewer babies overall had a morbidity recorded (ICD-10-AM code other than the birth code) in the private sector compared to the public sector (40.0% vs 53.9%) due to the added clinical and social maternal complexity in the public sector. There were however some significant differences noted under the main ICD-10-AM Grouping *Certain conditions originating in the perinatal period (P00-P96)*. Babies born in a private hospital were more likely to have been affected by a forceps or vacuum delivery and were more likely to have trauma to the scalp (3.22% vs 2.22%), intrauterine hypoxia (1.70% vs 1.21%), jaundice (4.68% vs 2.89%), minor cardiac murmurs (0.24% vs 0.17%), conjunctivitis (2.98% vs 1.27%), respiratory conditions (0.96% vs 0.57%), temperature regulation issues (2.08% vs 1.27%) feeding difficulties (3.83% vs 2.32%), carbohydrate metabolism issues (0.86% vs 0.53%), vomiting (0.55% vs 0.39%) and to be circumcised during the birth admission if a male (5.31% vs 0.21%)). Babies born in a public hospital were more likely to be admitted for observation and evaluation (6.35% vs 3.75%) have prophylactic antibiotics (0.52% vs 0.16%) and be admitted for socioeconomic circumstances (eg. housing, distance, adoption, assumption of care) (0.57% vs 0.05%) (Table 5)

Reason for birth re-admission of neonate up to 28 days of age

We examined the reasons for transfer or readmission of babies up until 28 days of age and found, that though the numbers are small, more babies born in private hospitals were readmitted compared to babies born in a public hospital (0.95% vs 0.65%) (Table 6). Babies born in private hospitals were more likely to be readmitted for infectious diseases (0.21% vs 0.12%) endocrine, nutritional and metabolic disorders (0.05% vs 0.02%), sleep disorders (0.03% vs 0.01%), hypoglycaemia (0.02% vs 0.01%), birth trauma such as cephalohaematoma (0.02% vs 0.01%); trauma involving the scalp (0.05% vs 0.02%), excessive crying (0.14% vs 0.07%), behavioural disorders (0.06% vs 0.02%) and for circumcision if a male (0.20 vs 0.13)). Babies born in public hospitals were more likely to be readmitted with respiratory disorders (0.27% vs 0.20%), injury and poisoning (eg burns) (0.05% vs 0.03%), antibiotic therapy (0.03% vs 0.01%) and socioeconomic circumstances (housing, distance, adoption, assumption of care (0.09% vs 0.04%).

For peer review only - http://bmjopen.bmj.com/site/about/guidelines.xhtml

Combined birth and readmission neonatal morbidity for selected codes

When we combined major birth and readmission morbidities for key selected codes we found that in the first 28 days following birth, babies born in private hospitals were significantly more likely to be admitted for feeding difficulties (4% vs 2.4%), circumcision if a male (5.6 vs 0.3)), birth trauma (mostly scalp trauma) (5% vs 3.6%), jaundice (4.8% vs 3.0%), hypoxia (1.7% vs 1.2%), respiratory disorders (1.2% vs 0.8%) and sleep/behavioural issues (0.2% vs 0.1%). Babies born in public hospitals were more likely to be admitted for socioeconomic circumstances such as housing, distance, adoption or assumption of care (0.7% vs 0.1%) and prophylactic antibiotics (0.6% vs 0.2%) (Table 7 & Figure 2).

Discussion

Intervention rates

Despite being an extremely low risk cohort, less than half the primiparous women in this study giving birth in a private hospital had a normal vaginal birth (45% vs 65%); this was 20% lower than in the public cohort. One in five primiparous women giving birth in a private hospital were induced and nearly one in two had an episiotomy. For low risk multiparous women giving birth in a private hospital nearly one in three were induced. The trend for higher intervention rates has been reported for low risk women giving birth in the private sector in Australia previously and continues to show an increase [9, 14, 15]. In a recent publication we showed that the rate of caesarean section had increased in both the private and public sector in the past decade in low risk women [9]. It has been argued in a previous publication that these high intervention rates in the private sector led to better perinatal outcomes than in the public sector [2]. This publication received significant criticism in letters to the editor [3, 16, 17] for several methodological flaws, including most significantly the failure to adjust for low birth weight, inadequate ascertainment of congenital abnormalities and failure to look at perinatal morbidity. In this study we included only low risk women, adjusting for maternal age and gestational age differences. We also

For peer review only - http://bmjopen.bmj.com/site/about/guidelines.xhtml

removed all babies with congenital abnormalities from this data set. We found that the perinatal mortality rate was not statistically different when the populations were matched in this data set for maternal risk.

Neonatal resuscitation and admission to SCN/NICU

We found that babies born in a private hospital were much more likely to experience some form of resuscitation, in particular twice the rate of suctioning at birth. Routine suctioning for infants born with clear and/or meconium stained amniotic fluid is not recommended [18] as it can cause a bradycardia [19] and there is no evidence of benefit. We are unsure why such a high rate of newborn suctioning continues in the private sector. While rates of Apgar scores of ≤7 at five minutes were slightly higher amongst primiparous women who gave birth in public hospital (but not multiparous women), and this has been demonstrated in another recent Australian publication [15], overall the babies were no more likely to be admitted to SCN/NICU compared to babies born in private hospitals.

Neonatal admission and readmission

We found some interesting differences in morbidity however when examining morbidity attached to the birth admission and readmission to hospital in the first 28 days for codes that may be associated with the higher rates of obstetric intervention in the private sector and a different sociodemographic profile in the public sector. While increasingly preterm babies >35 weeks/ and or >2.2k and some cases of jaundice may be managed at the bedside in some hospitals, this is less likely to occur in a private hospital. It is more likely to occur in large maternity units.

Birth trauma, in particular injuries to the scalp, were significantly in the private sector and these are generally associated with instrumental birth, including vacuum extraction [20-22]. With more women (nearly one in three primiparous women) experiencing an instrumental birth in the private cohort and one in five women in the public sector this is not surprising. Birth trauma is associated with

For peer review only - http://bmjopen.bmj.com/site/about/guidelines.xhtml

a longer hospital stay and increased risk of admission to SCN/NICU as well as higher rates of neonatal morbidity including neurological morbidity (hypotonia, jitteriness, convulsions and hypoxic ischaemic encephalopathy) and jaundice [22].

Jaundice was observed to be higher in the private sector, which may be related to several factors, such as the elective delivery of babies at an earlier gestation, the increased scalp trauma due to the high instrumental birth rate, as discussed above, and potential breastfeeding difficulties due to higher use of epidural analgesia. Jaundice has been associated with birth trauma, in particular delivery by vacuum extraction, and problems with feeding, especially supplementary feeding [22, 23]. Earlier gestational age <39 weeks has also be found to be associated with jaundice, with this decreasing with each week of additional gestation [24]. The gestational age of babies born in private hospitals in this study was significantly lower than in the public sector due to the high rates of non-medical induction of labour and non-medically indicated caesarean section before the onset of labour.

While there have been studies associating difficulties with breastfeeding and higher rates of jaundice, the recent publication from the Universal Screening for Hyperbilirubinemia Study Group found difficulties with breastfeeding was a minimal risk factor [24].

Nearly twice as many babies who were born in a private hospital in this study were admitted or re-admitted with feeding problems compared to babies born in a public hospital. Feeding difficulties are associated with operative birth interventions and being early term [25, 26]. Breastfeeding outcomes are positively associated with uncomplicated unassisted vaginal birth where the mother and infant remain together and breastfeeding is started within an hour of the birth and following skin-to-skin contact. Interventions during labour and birth can impact on the initiation and duration of breastfeeding. Caesarean section [27-30], instrumental birth [31], epidural anaesthesia and opioid analgesia [32-34] use have all been associated with breastfeeding difficulties. All these birth interventions were higher in the private cohort in this study. In addition early term birth, which is mainly due to induction of labour and elective caesarean section (35, 36) is associated with increased breastfeeding difficulties along with other serious morbidities [37]. Unmedicated newborns are more highly aroused immediately following the birth [38] and able to breastfeed without assistance if given skin to skin contact and

For peer review only - http://bmjopen.bmj.com/site/about/guidelines.xhtml

freedom from intrusive procedures [39, 40]. Following caesarean section there can be a significantly longer period of time until a mother touches and holds her newborn compared to an unassisted vaginal birth [41].

In a previous paper [6] using national Australian population data we found that among low-risk women who had an unassisted vaginal birth with spontaneous onset of labour and no labour augmentation, the odds of admission to neonatal intensive care or special care nursery were significantly increased when the baby was 37 weeks gestation at the time of birth compared to later gestations. Some claim that during the final weeks of gestation the fetal brain goes through a marked increase in mass and nerve growth (corticoneurogenesis) which may be best left undisturbed by allowing the normal gestational length to occur [42]. In this study low risk women giving birth in private hospitals in NSW were much more likely to give birth at earlier gestations than their public hospital counterparts for every week up to and including 40 weeks, but they were significantly less likely to deliver at 41 weeks. This may also help to explain why more babies born in a private hospital were readmitted with, respiratory, feeding, jaundice and sleep and behavioural problems. However there is also evidence that there are increased adverse perinatal outcomes for babies born following 41 completed weeks, but we did not examine this population [43].

Circumcision

Babies born in a private hospital were significantly more likely to be circumcised in the first 28 days of life. This may be due to different information being given in private hospitals about the procedure or easy access to providers who perform the procedure. Circumcision rates are estimated to be between 10-20% in Australia [44] and are decreasing. A recent position statement of the Royal Australian College of Physicians states “that the frequency of diseases modifiable by circumcision, the level of protection offered by circumcision and the complication rates of circumcision do not warrant routine infant circumcision in Australia and New Zealand” [44].

For peer review only - http://bmjopen.bmj.com/site/about/guidelines.xhtml

Socioeconomic circumstances

The difference in the socioecononomic status of the women giving birth in public compared to private hospitals appears to be demonstrated by the significantly higher rates of public hospital babies with a morbidity attached to the birth admission or readmission in the first 28 days for socioeconomic circumstances, including housing, distance, adoption and assumption of care. This again confirms what is already known that the two populations are very different sociodemographically with greater disadvantage in the public sector.

Limitations

Our study is limited to providing a snapshot of perinatal outcomes in the most populous state in Australia in a defined time period for women who have no indicated risk at birth. However, this study provides useful data following on from our previous paper looking at obstetric intervention in private and public hospitals in NSW providing the reader with a detailed picture of perinatal mortality and morbidity. The advantages of using population-based datasets such as the PDC and the linkage to four other population-based databases include the size of the sample and the high level of accuracy of a validated dataset. The limitations are the restricted number of variables that are included and the scarcity of specific information on potential influencing variables. A small number of cases with a low linkage rate (0.3%) were not included and so there is the possibility of missing adverse outcomes. A previous study showed that where stillbirths are excluded due to low linkage these are at lower gestational ages and not term infants as were the focus in this study [45]. Previous validation studies have reported high levels of data accuracy for the majority of diagnoses and procedures conducted during labour and delivery in the state-wide data base [46, 47], although the recording of medical conditions and smoking are overall generally underreported [46, 48]. Having a linked data set provides a much richer picture than we have had previously of the morbidity and

For peer review only - http://bmjopen.bmj.com/site/about/guidelines.xhtml

mortality associated with birth interventions. While we could not control for obesity due to lack of data, women who have private health insurance have lower rates of obesity and higher socioeconomic status, hence these health disadvantages are most likely over represented in the public women [49]. There are also several other socio-demographic factors we could not control for, such as education and income, that increase risk for the women giving birth in public hospitals. This study can only provide an overview of possible associations between obstetric interventions and neonatal outcomes and does not imply causality, which could be better obtained from prospective cohort studies.

Conclusion

The continual rise in obstetric intervention for low risk women in Australia may be contributing to increased morbidity for healthy women and babies and higher cost of health care. The fact that these procedures which were initially life-saving are now so commonplace and do not appear to be associated with improved rates of perinatal mortality or morbidity demands close review. Early term delivery and instrumental births may be associated with increased morbidity in neonates and this requires urgent attention.

For peer review only

Table 1. Maternal characteristics, interventions and outcomes for low risk primiparous women in NSW (2000-2008)

Low risk Primiparous women	Private Hospital (n=29597)	Public Hospital (n=79792)
**Maternal age (years)**		
20-24	6.2%	28.9%
25-29	39.9%	40.9%
30-34	53.9%	30.2%
Weeks gestation at delivery		
37	4.5%	4.3%
38	15.4%	11.8%
39	27.8%	25.0%
40	43.2%	39.7%
41	9.1%	19.2%

For peer review only - http://bmjopen.bmj.com/site/about/guidelines.xhtml

**Type of labour**		
Spontaneous	71.9%	89.9%
Induced	19.2%	7.1%
No labour	8.9%	3.0%
**Delivery**		
Normal vaginal	44.9%	64.8%
Forceps	11.5%	6.7%
Vacuum	18.9%	12.9%
Total caesarean section	24.7%	15.6%
Caesarean section (after labour)	15.9%	12.6%
Caesarean section before the onset of labour	8.8%	3.0%
**Epidural**	70.8%	35.4%
**Episiotomy**	42.4%	23.3%
**Severe perineal trauma**	4.7%	5.4%

For peer review only

For peer review only - http://bmjopen.bmj.com/site/about/guidelines.xhtml

Table 2. Maternal characteristics, interventions and outcomes for low risk multiparous women in NSW (2000-2008)

**Low risk Multiparous women**	**Private Hospital (n=28703)**	**Public Hospital (n=99212)**
**Maternal age (years)**		
20-24	2.1%	16.8%
25-29	25.8%	38.8%
30-34	72.1%	44.4%
Weeks gestation at delivery		
37	4.1%	4.0%
38	18.7%	13.0%
39	31.6%	26.9%
40	40.2%	40.8%
41	5.4%	15.3%

For peer review only

For peer review only - http://bmjopen.bmj.com/site/about/guidelines.xhtml

**Type of labour**		
Spontaneous	64.0%	87.4%
Induced	32.1%	10.1%
No labour	3.9%	2.5%
**Delivery**		
Normal vaginal	86.1%	92.7%
Forceps	1.9%	0.7%
Vacuum	6.1%	2.1%
Total caesarean section	5.9%	4.5%
Caesarean section after labour	2.0%	2.0%
Caesarean section before the onset of labour	3.9%	2.5%#
**Epidural**	34.4%	9.5%
**Episiotomy**	16.2%	5.1%
**Severe perineal trauma**	0.9%	0.9%

For peer review only - http://bmjopen.bmj.com/site/about/guidelines.xhtml

Table 3. Perinatal outcomes adjusted for maternal age and gestation at birth for low risk primiparous women

	Private	Public			
	(n=29597)	(n=79791)	OR**	AOR**	p
Apgar<7 at 5 minutes	296 (1.0%)	1037 (1.3%)	1.36 (1.12-1.54)	1.34(1.18-1.53)	<0.001
Any resuscitation*	18498 (62.5%)	30560 (38.3%)	0.372 (0.36-0.38)	0.364 (0.354-0.37)	<0.001
Admitted to SCN and/or NICU	3078 (10.4%)	8139 (10.2%)	1.00 (0.96-1.05)	1.03 (0.98-1.08)	0.210
Transferred	178 (0.6%)	3351 (4.2%)	7.30 (6.29-8.40)	7.55 (6.52-8.74)	<0.001

For peer review only - http://bmjopen.bmj.com/site/about/guidelines.xhtml

For peer review only

Total Perinatal Mortality	22 (0.74/1000)	85 (1.06/1000)	1.40 (0.93-2.01)	1.49 (0.93-2.41)	0.100

*Any resuscitation includes: Suction, oxygen, IPPR by bag and mask, Intubation and IPPR, external cardiac massage and ventilation and other

** Private hospital is the reference category

For peer review only

Table 4. Perinatal outcomes adjusted for maternal age and gestation at birth for low risk multiparous women

	Private	Public			
	(n=28703)	(n=99212)	OR**	AOR**	p
Apgar<7 at 5 minutes	67 (0.2%)	149 (0.2%)	1.56 (0.980-2.10)	1.42 (0.96-2.21)	0.562

Any resuscitation*	14820 (51.6%)	29867 (30.1%)	0.404 (0.39-0.42)	0.399 (0.39-0.46)	<0.001
Admitted to SCN and/or NICU	1775 (6.2%)	5870 (5.9%)	0.957 (0.91-1.01)	1.027 (0.97-1.09)	0.363
Transferred	232 (0.8%)	4375 (4.4%)	5.661 (4.96-6.47)	6.516 (5.70-7.45)	<0.001
Total Perinatal Mortality	17 (0.59/1000)	76 (0.77/1000)	1.294 (0.77-2.19)	1.294 (0.75-2.23)	0.355

*Any resuscitation includes: Suction, oxygen, IPPR by bag and mask, Intubation and IPPR, external cardiac massage and ventilation and other

** Private hospital is the reference category

For peer review only

For peer review only

Table 5. Morbidity associated with birth admission coded on neonatal birth admission record

		**Private**		**Public**		
**ICD-10-AM Grouping**		n=58300		n=179003		
**Certain conditions originating in the perinatal period (P00-P96)**		Count	%	Count	%	p
	Fetus and newborn affected by maternal infectious and parasitic diseases (P00.2)	41	0.07%	989	0.55%	<0.001
	Fetus and newborn affected by forceps delivery (P03.2)	473	0.81%	1108	0.62%	<0.001
	Fetus and newborn affected by delivery by vacuum extractor (P03.3)	511	0.88%	1509	0.84%	0.46
	**Birth trauma (all body systems) (P10-P15)**	**2948**	5.06%	**6447**	3.60%	<0.001
	- specifically to scalp (included in above total) (P12)	1880	3.22%	3965	2.22%	<0.001
	Intrauterine hypoxia (P20)	993	1.70%	2170	1.21%	<0.001
	Other specified respiratory conditions of newborn (P28)	562	0.96%	1015	0.57%	<0.001
	Benign and innocent cardiac murmurs in newborn (P29.82)	139	0.24%	303	0.17%	=0.001
	Neonatal conjunctivitis specific to the perinatal period (P39.1)	1740	2.98%	2267	1.27%	=0.001
	**Jaundice related conditions (P58-P59)**	**2728**	4.68%	**5166**	2.89%	<0.001
	Transitory disorders of carbohydrate metabolism specific to	502	0.86%	942	0.53%	<0.001

	fetus and newborn (P70)					
	Conditions involving the integument & temperature regulation of fetus and newborn (P80-P83)	1214	2.08%	2275	1.27%	<0.001
	Vomiting in newborn (P92.0)	320	0.55%	693	0.39%	<0.001
	Feeding problems in newborn (P92)	2231	3.83%	4157	2.32%	<0.001
**factors influencing health status and contact with health services (Z00-Z99)**						
	Routine and ritual circumcision (Z41.2)*	1552	5.31%	187	0.21%	<0.001
	Observation and evaluation of newborn (Z03)	2187	3.75%	11372	6.35%	<0.001
	Prophylactic chemotherapy (antibiotics) (Z29.2)	93	0.16%	935	0.52%	<0.001
	Socioeconomic circumstances (housing, distance, adoption, assumption of care) (Z76)	32	0.05%	1020	0.57%	<0.001

* as a % of male babies

For peer review only

Table 6. Morbidity associated with readmission of the baby ≤28 days of age

		Private		Public		
		n=58300		n=179003		
**ICD-10-AM Grouping**		Count	%	Count	%	**p**
**Certain infectious and parasitic diseases (A00-B99)**		**121**	0.21%	**217**	0.12%	<0.001
**Endocrine nutritional and metabolic diseases (E00-E89)**		**30**	0.05%	**38**	0.02%	<0.001
	Volume depletion (E86)	18	0.03%	12	0.01%	<0.001
**Mental and behavioural disorders (F00-F99)**		**33**	0.06%	**30**	0.02%	<0.001
	Non-organic hypersomnia (F51.1)	17	0.03%	19	0.01%	=0.002
**Diseases of the nervous system (G00-G99)**		**34**	0.06%	**38**	0.02%	<0.001
	Disorders of the sleep wake schedule (G47.2)	18	0.03%	9	0.01%	<0.001
**Diseases of the ear and mastoid process (H60-H95)**		**19**	0.03%	**23**	0.01%	=0.002
**Diseases of the respiratory system (J00-J99)**		**155**	0.27%	**351**	0.20%	=0.002
	Acute obstructive laryngitis (croup) (J05)	6	0.01%	16	0.01%	=0.96
	Acute upper respiratory infection unspecified (J06)	16	0.03%	52	0.03%	=0.96

For peer review only - http://bmjopen.bmj.com/site/about/guidelines.xhtml

	Pneumonia (J10-J18)	6	0.01%	19	0.01%	=0.96
	Acute bronchiolitis (J21)	70	0.12%	175	0.10%	=0.17
	Unspecified acute lower respiratory tract infection (J22)	3	0.01%	12	0.01%	*
**Diseases of the digestive system (K00-K93)**		**53**	0.09%	**92**	0.05%	=0.001
	Gastro-oesophageal reflux disease (K21)	26	0.04%	37	0.02%	=0.003
**Certain conditions originating in the perinatal period (P00-P96)**		**474**	0.81%	**1011**	0.56%	<0.001
	Cephalohaematoma due to birth trauma (P12.0)	14	0.02%	13	0.01%	=0.002
	Total birth trauma to scalp (P12)	30	0.05%	32	0.02%	<0.001
	Intrauterine hypoxia (P20)	4	0.01%	25	0.01%	*
	Other neonatal hypoglycaemia (P70.4)	14	0.02%	14	0.01%	=0.002
	Other transitory neonatal electrolyte and metabolic disturbances (P70.8)	5	0.01%	21	0.01%	=0.527
	Fever of newborn (P81.9)	5	0.01%	18	0.01%	0.753
	Feeding problems of newborn (P92)	40	0.07%	100	0.06%	=0.271
	**Neonatal jaundice (P58)**	**193**	0.33%	**90**	0.05%	<0.001
**Symptoms, signs and abnormal findings not elsewhere classified (R00-R99)**		**198**	0.34%	**340**	0.19%	<0.001

For peer review only - http://bmjopen.bmj.com/site/about/guidelines.xhtml

	Fever (R50)	19	0.03%	27	0.02%	=0.008
	Feeding difficulties and mismanagement (R63.3)	25	0.04%	37	0.02%	=0.003
	Excessive crying (R68.1)	83	0.14%	117	0.07%	<0.001
**Injury, poisoning and certain other consequences of external causes (S00-T98)**		**15**	0.03%	**94**	0.05%	=0.009
	Burns (T20-T31)	0	0.00%	30	0.02%	*
**Factors influencing health status and contact with health services (Z00-Z99)**		**192**	0.33%	**691**	0.39%	=0.051
	Observation and evaluation in newborn (Z03)	34	0.06%	102	0.06%	=0.907
	Prophylactic chemotherapy (antibiotics) (Z29.2)	5	0.01%	49	0.03%	=0.009
	Routine and ritual circumcision (Z41.2)	68	0.20%	119	0.13%	<0.001
	Attention to surgical dressings and sutures (Z48.0)	0	0.00%	28	0.02%	*
	Socio-economic circumstances (housing, distance, adoption, assumption of care) (Z76)	22	0.04%	156	0.09%	<0.001

***cell size too small to calculate chi-square ** as a % of male babies**

For peer review only - http://bmjopen.bmj.com/site/about/guidelines.xhtml

Table 7. Combined birth and readmission neonatal morbidity for selected codes

	**Private**	**Public**	**P***
Total feeding difficulties	2314 (4.0%)	4306 (2.4%)	<0.0001
Total circumcision**	1620 (5.6%)	306 (0.3%)	<0.0001
Total socio-economic circumstances	54 (0.1%)	1176 (0.7%)	<0.0001
Total birth trauma	2922 (5.0%)	6492 (3.6%)	<0.0001
Total hypoxia	997 (1.7%)	2195 (1.2%)	<0.0001
Total jaundice	2818 (4.8%)	5359 (3.0%)	<0.0001
Total respiratory	717 (1.2%)	1366 (0.8%)	<0.0001
Total sleep/behavioural issues	118 (0.2%)	145 (0.1%)	<0.0001
Prophylactic antibiotics	98 (0.2%)	982 (0.6%)	<0.001

*chi-square ** as a % of male babies

For peer review only

Contributorship Statement: HD led the study and wrote the paper. ST helped in constructing the study design and writing the paper. MT helped in constructing the study design. AB helped in writing the paper. CB gave biostatistical support and helped in writing the paper. CT helped with study design, analysis and writing of the paper.

Competing Interests: None

Data Sharing Statement: There is no additional data available

For peer review only

References

1 Li Z, Zeki R, Hilder L, Sullivan EA. Australia's mothers and babies 2011. Perinatal statistics series no. 28. Cat. no. PER 59. In: Unit ANPEaS, editor. Canberra2013.

2 Robson SJ, Laws P, Sullivan EA. Adverse outcomes of labour in public and private hospitals in Australia: a population based descriptive study. The Medical Journal of Australia 2009;190(9):474-7.

3 Tracy S, Welsh A, Dahlen H, et al. Letter to the Editor re Robson SJ, Laws P, Sullivan EA. Adverse outcomes of labour in public and private hospitals in Australia: a population-based descriptive study. Med J Aust 2009; 190: 474-477. Medical Journal of Australia. 2009;191(10):579-80.

4 Clark E.A.S, Silver RM. Long-term maternal morbidity associated with repeat caesarean delivery. American Journal of Obstetrics and Gynecology. 2011;S2(December).

5 Hyde MJ, Mostyn A, Modi N, et al. The health implications of birth by caesarean section. Biological Reviews. 2012;87(1):229-43.

6 Tracy S, Tracy M, Sullivan E. Admission of Term Infants to Neonatal Intensive Care: A Population-Based Study. Birth 2007;34(4):301-7.

7 Tracy S, K , Tracy M. Costing the cascade: estimating the cost of increased obstetric intervention in childbirth using population data. British Journal of Obstetrics and Gynaecology. 2003;110:717-24.

8 Allen VM, O'Connell CM, Farrell SA, et al. Economic implications of method of delivery. Am J Obstet Gynecol 2005;193(1):192-7.

9 Dahlen HG, Tracy S, Tracy M, et al. Rates of obstetric intervention among low-risk women giving birth in private and public hospitals in NSW: a population-based descriptive study. BMJ Open. 2012;2:e001723 doi:10.1136/bmjopen-2012-001723.

10 Roberts C, L,, Tracy S, Peat B. Rates of obstetric intervention among private and public patients in Australia: population based descriptive study. British Medical Journal. 2000;312:137-41.

11 Buist R. Letter to the Editor: Private Obstetrics-again. BMJ Open. 2012;2(5):e001723 doi:10.1136/bmjopen-2012-.

12 CHeReL. Centre for Health Record Linkage (CHeReL). Quality Assurance Report 2012. http://wwwcherelorgau/media/24160/qa_report_2012pdf. 2012.

13 Lain SJ, Hadfield RM, Raynes-Greenow CH, et al. Quality of data in perinatal population health databases: a systematic review. Med Care. 2012;50(4):e7-e20.

For peer review only

14 Roberts CL, Tracy S, Peat B. Rates for obstetric intervention among private and public patients in Australia: population based descriptive study. British Medical Journal. 2000 2000;321(7254):137-41.

15 Einarsdóttir K, Stock S, Haggar F, et al. Neonatal complications in public and private patients: a retrospective cohort study. BMJ Open. 2013;Downloaded from bmjopen.bmj.com on November 11, 2013.

16 Evans N, Malcolm G, Gordon A. Letter to the Editor re Adverse outcomes of labour in public and private hospitals in Australia. MJA. 2009;191(10):579.

17 Chambers D. Letter to the Editor re Adverse outcomes of labour in public and private hospitals in Australia. MJA. 2009;191(10):578-9.

18 Wyllie J. Recent changes to UK newborn resuscitation guidelines. Arch Dis Child Fetal Neonatal Ed. 2012;97(1):F4-F7.

19 Kannapiran R, MKennea N. Resuscitation of the newborn. Obstetrics, Gynecology & Reproductive Medicine. 2012;22(4):92-7.

20 Doumouchtsis SK, Arulkumaran S. Head injuries after instrumental vaginal deliveries. Current Opion in Obstetrics and Gynaecology. 2006;18:129-34.

21 Towner D, Castro MA, Wilkens EE, et al. Effect of Mode of Delivery in Nulliparous Women on Neonatal Intracranial Injury. The New England Journal of Medicine 1999;341:1709-14.

22 Linder N, Linder I, Fridman E, et al. Birth trauma – risk factors and short-term neonatal outcome. The journal of Maternal-fetal & Neonatal Medicine. 2013;DOI: 10.3109/14767058.2013.789850.

23 Bertini G, Dani C, Tronchin M, et al. Is breastfeeding really favouring early neonatal jaundice? Pediatrics. 2006;107(3):e41.

24 Bhutani VK, Stark AR, Lazzeroni LC, et al. Predischarge Screening for Severe Neonatal Hyperbilirubinemia Identifies Infants Who Need Phototherapy. The Journal of Pediatrics. 2013;162(3):477-82.

25 Young PC, Korgenski K, Buchi KF. Early Readmission of Newborns in a Large Health Care System. Pediatrics. 2013;131(5):e1538-e44.

26 Brown A, Jordan S. Impact of birth complications on breastfeeding duration: an internet survey. Journal of Advanced Nursing. 2012;69(4):828-39.

27 Chapman D.J, Perez-Escamilla R. Identification of risk factors for delayed onset oflactation. Journal of the American Dietetic Association. 2003;99:450-4.

For peer review only

28 Dewey K.G., Nommsen-Rivers L.A., Heinig M.J, et al. Risk factors for suboptimal infant breastfeeding behavior, delayed onset of lactation and excess neonatal weight loss. Pediatrics 2003;112(3):607-19.

29 Scott J.A., Binns C.W, Oddy W.H. Predictors of delayed onset of lactation. Maternal and Child Nutrition. 2007;3(3):186-93.

30 Jordan S., Emery S., Watkins A., et al. Associations of drugs routinely given in labour with breastfeeding at 48 hours: analysis of the Cardiff Births Survey. British Journal of Gynecology. 2009;116(12):1622-9.

31 Leung G.M, Lam T.H, Ho L.M. Breast-feeding and its relation to smoking and mode of delivery. Obstetrics and Gynecology 2002;99(5):785-94.

32 Jordan S. Infant feeding and analgesia in labour: the evidence is accumulating. International Breastfeeding Journal Research. 2006;1(25):doi: 10.1186/746-4358-1-25.

33 Henderson J.J., Dickinson J.E., Evans S.F., et al. Impact of intrapartum epidural analgesia on breast- feeding duration. Australian and New Zealand Journal of Obstetrics and Gynaecology. 2003;43(5):372–7.

34 Beilin Y., Bodian C., Weiser J., et al. Effect of labor analgesia with and without fentanyl on infant breastfeeding: a prospective, randomized double-blind study. Anesthesiology. 2005;103(6):1211– 7.

35 Ohnsorg T, Schiff J. Preventing elective induction before 39 weeks. , 93(11), 44-46. Minnesota Medicine. 2010;93(11):44-6.

36 Oshiro BT, Henry E, Wilson J, et al. Decreasing elective deliveries before 39 weeks of gestation in an integrated health care system. Obstetrics & Gynecology. 2009;113:804-11.

37 Reddy UM, Ko CW, Willinger M. "Early term" births (37-38 weeks) are associated with increased mortality. American Journal of Obstetrics & Gynecology. 2006;195(S202).

38 McLaughlin FJ, O'Connor S, Deni R. Infant state and behavior during the first postpartum hour. The Psychological Record 1981;31:455–8.

39 Widstrom A-M, Ransjo-Arvidson AB, Christensson K, et al. Gastric suction in healthy newborn infants. Effects on circulation and developing feeding behaviour. Acta Paediatr. 1987;76:566–72.

40 Righard L. How do newborns find their mother's breast? . Birth. 1995;22:174–5.

41 Fisher J, Astbury J, Smith A. Adverse psychological impact of opera- tive obstetric interventions: a prospective study. Aust NZ J Psychiatry 1997;31:728–38.

For peer review only

42 Adams-Chapman I. Insults to the developing brain and impact on neurodevelopmental outcome. Journal of Communication Disorders. Journal of Communication Disorders. 2009;42:256-62.

43. Gülmezoglu AM, Crowther CA, Middleton P, et al. Induction of labour for improving birth outcomes for women at or beyond term. *Cochrane Database of Systematic Reviews* 2012;Issue 6. Art.No.:CD004945. DOI: 10.1002/14651858.CD004945.pub3.

44. The Royal Australian College of Physicians. Circumcision of infant males. In: Physicians TRACo, editor. Sydney: The Royal Australasian College of Physicians; 2010.

45. Bentley JP, Ford JB, Taylor LK, et al. Investigating linkage rates among probabilistic linked births and hospital records. BMC Medical Research Methodology. 12:149, 2012.

46. Taylor L, Travis S, Pym M, et al. How useful are hospital morbidity data for monitoring conditions occurring in the perinatal period? Australian & New Zealand Journal of Obstetrics & Gynaecology. 2005;45:36 –41.

47. Roberts C, Bell J, Ford J, et al. Monitoring the quality of maternity care: how well are labour and delivery events reported in population health data? . Paediatirc and Perinatal Epidemiology. 2008;23:144-52.

48. Thornton C, Makris A, Ogle R, et. al. Generic obstetric database systems are unreliable for reporting the Hypertensive Disorders of Pregnancy. Australian and New Zealand Journal Obstetrics and Gynaecology. 2004;44(505-509).

49. Finkelstein A, Fiebelkorn IC, Wang G. National medical Spending attributable to overweight and obesity: How much and who's paying. Health Affairs, 2003 Project HOPE–The People-to-People Health Foundation, Inc., 219-226. 2003.

Figure legends:

Figure 1: Comparison of gestation at delivery between all low risk women stratified bu hospital type.

Figure 2: Birth trauma as a percentage of all births in Private and Public Hospitals.

For peer review only

Rates of obstetric intervention and associated perinatal mortality and morbidity amongst low risk women giving birth in private and public hospitals in NSW (2000-2008): A linked data population based cohort study.

Hannah G. Dahlen RN, RM, BN (Hons), MCommN, PhD, FACM

Associate Professor of Midwifery

School of Nursing and Midwifery

Family and Community Health Group

Locked Bag 1797

Penrith South DC

NSW

Australia

Ph: +61 2 96859118

h.dahlen@uws.edu.au

Sally Tracy

Professor Midwifery

Professor of Midwifery

University of Sydney,

Royal Hospital for Women, Sydney

Tel: 0420277106

For peer review only - http://bmjopen.bmj.com/site/about/guidelines.xhtml

For peer review only

Mark Tracy

Senior Newborn Intensive Care Specialist and HOD

CNBC Westmead Hospital

Senior Clinical Lecturer Paediatrics and Child Health

University of Sydney

Tel (02) 9845 6568

Andrew Bisits Mb bs(unsw)franzcog ,mmed stat ,dip clin epi

Acting Medical Co Director of Maternity, Lead Clinician Birthing Suite

Royal Hospital for Women

Conjoint Associate Professor School of Women and Children's Health University of NSW

Randwick NSW 2033

0419418492

Chris Brown, BSc, MBiostat

Biostatistician

NHMRC Clinical Trials Centre

Locked Bag 77

Camperdown NSW 1450

For peer review only

For peer review only - http://bmjopen.bmj.com/site/about/guidelines.xhtml

Charlene Thornton BN, Grad.Dip.Mid, M Sc Med (Research), M Sc Med (Clin Epi), PhD

Post Doctoral Research Fellow

School of Nursing and Midwifery

Family and Community Health Group

Locked Bag 1797

Penrith South DC

NSW

Australia

Keywords: low risk primiparous, low risk multiparous , induction, vaginal birth, obstetric care, caesarean, birth trauma

Word count 4596

Ethical approval was obtained from the NSW Population and Health Services Research Ethics Committee, Protocol No.2010/12/291.

Ni funds allocated

Nil extra data

For peer review only - http://bmjopen.bmj.com/site/about/guidelines.xhtml

Rates of obstetric intervention and associated perinatal mortality and morbidity amongst low risk women giving birth in private and public hospitals in NSW (2000-2008): A linked data population based cohort study.

Abstract

Objectives: To examine the rates of obstetric intervention and associated perinatal mortality and morbidity in the first 28 days amongst low risk women giving birth in private and public hospitals in NSW (2000-2008).

Design: Linked data population based retrospective cohort study involving five data sets.

Setting: New South Wales, Australia.

Participants: 691,738 women giving birth to a singleton baby during the period 2000 to 2008.

Main outcome measures: Rates of neonatal resuscitation, perinatal mortality, neonatal admission following birth and readmission to hospital in the first 28 days of life in public and private obstetric units.

Results: Rates of obstetric intervention amongst low risk women were higher in private hospitals, with primiparous women 20% less likely to have a normal vaginal birth compared to the public sector. Neonates born in private hospitals were more likely to be less than 40 weeks; more likely to have some form of resuscitation; less likely to have an Apgar <7 at five minutes (if a first baby). Neonates born in private hospitals to low risk mothers were more likely to have a morbidity attached to the birth admission and to be readmitted to hospital in the first 28 days for birth trauma (5% vs 3.6%); hypoxia (1.7% vs 1.2%) ; jaundice (4.8% vs 3%); feeding difficulties (4% vs 2.4%) ; sleep /behavioural issues (0.2% vs 0.1%); respiratory conditions (1.2% vs 0.8%) and circumcision (5.6 vs 0.3%) but they were less likely to be admitted for prophylactic antibiotics

For peer review only - http://bmjopen.bmj.com/site/about/guidelines.xhtml

(0.2% vs 0.6%) and for socioeconomic circumstances (0.1% vs 0.7%). Rates of perinatal mortality were not statistically different between the two groups.

Conclusion:

High rates of obstetric intervention amongst low risk women in private hospitals appear to be associated with higher rates of morbidity seen in the neonate and no evidence of a reduction in perinatal mortality.

For peer review only

For peer review only - http://bmjopen.bmj.com/site/about/guidelines.xhtml

Article focus:

- To examine the rates of obstetric intervention and associated perinatal mortality and morbidity in the first 28 days amongst low risk women giving birth in private and public hospitals in NSW (2000-2008) using linked data.
- Rates of neonatal resuscitation, perinatal mortality, neonatal admission following birth and readmission to hospital in the first 28 days of life amongst those infants born in public and private obstetric units.

Key messages

- Babies born to low risk mothers in private hospitals are more likely to require some resuscitation compared to those born in public hospitals.
- For babies born in private hospitals to low risk mothers there are higher rates of morbidity such as birth trauma, hypoxia, jaundice, feeding difficulties, sleep/behavioural issues, respiratory conditions and circumcision. For babies born in public hospitals there are higher rates of antibiotic use and admission for socio-economic reasons (eg housing, distance, adoption and assumption of care)
- Rates of perinatal mortality were comparable between babies born to low risk women giving birth in private and public hospitals

Strengths and limitations of this study

- The strength of this study lies in the large sample size of linked birth data admissions associated with these births.
- The use of data from five population based datasets which have been linked to enhance validity and ascertainment
- Limitations are the restricted number of variables that are included and the scarcity of specific information on potential confounders.
- Body mass index and key sociodemographic risk factors could not be controlled for and this would have added risk to women giving birth in public hospitals.

For peer review only - http://bmjopen.bmj.com/site/about/guidelines.xhtml

For peer review only

Introduction

In Australia, the national statistics reveal that 29% (n =83,573) of women who gave birth in hospital gave birth in private hospitals directly under private obstetric care [1}. The remaining 71% (n=204,399) of women gave birth in public hospitals in Australia. Women who are privately insured have been reported to have better maternal and perinatal outcomes compared to women who give birth in public hospitals as public patients [2]; but it has been argued that these women tend to be less socioeconomically disadvantaged and healthier [3] and therefore might be expected to have better outcomes. Arguments about the impact of private status on health outcomes are in reality complex.

What is not disputed are the much higher rates of obstetric intervention that occur in private hospitals in Australia. At a national level, the intervention rates in childbirth, such as caesarean section, are significantly higher in the private sector (43.1% vs 28.4%) and the rates of normal vaginal birth significantly lower (42.7% vs 61%) [1]. Despite the rising intervention rates over the past decade, the perinatal mortality rate overall has not shown a corresponding decline. There is also growing concern that the short and long term morbidity associated with major obstetric interventions, such as caesarean, may not be insignificant for the mother [4] and the baby [5, 6]. The cost to the tax payer of the rising intervention in childbirth is also significant [7, 8].

A recent study in New South Wales, Australia [9] found among 293,840 low-risk women, rates of obstetric intervention were highest in private hospitals and lowest in public hospitals. Low-risk primiparous women giving birth in a private hospital compared to a public hospital had higher rates of induction (31% vs 23%); instrumental birth (29% vs 18%); caesarean section (27% vs 18%), epidural (53% vs 32%), episiotomy (28% vs 12%) and lower normal vaginal birth rates (44% vs 64%). Low-risk multiparous women had higher rates of instrumental birth (7% vs 3%), caesarean section (27% vs 16%), epidural (35% vs 12%), episiotomy (8% vs 2%) and lower normal vaginal birth rates (66% vs 81%). Following a comparison with data from the decade previously [10], these interventions were found to have increased by 5% for women in public hospitals

For peer review only - http://bmjopen.bmj.com/site/about/guidelines.xhtml

and by over 10% for women in private hospitals [9]. Among low-risk primiparous women giving birth in private hospitals, 15 per 100 women had a vaginal birth with no obstetric intervention compared to 35 per 100 women giving birth in a public hospital [9]. Concern was expressed that perinatal mortality and morbidity was not reported in that paper [11]. In this study we aim to address this through examining the rates of obstetric intervention and associated perinatal mortality and morbidity attached to the birth admission and readmission to hospital in the first 28 days of life for low risk women giving birth in private and public hospitals in NSW (2000-2008).

Methods

Data Sources

The New South Wales Centre for Health Record Linkage conducted linkage of several datasets via the Health Record Linkage (CHeReL). Pregnancy and birth data for the time period July 1st 2000 till June 2nd 2008 of all singleton births were provided by New South Wales (NSW), Ministry of Health as recorded in the NSW Midwives Data Collection (MDC), now the Perinatal Data Collection (PDC). This population based surveillance system contains maternal and infant data on all births of greater than 400 grams birth weight and/or 20 completed weeks gestation. Hospitals are coded either as private or public in the data set. However, the data identifying women who received care in public hospitals under private accommodation status is no longer collected as it had been in the years 1996-97 and for this reason patients who are under private obstetric care in public hospitals are not able to be differentiated from their public counterparts, so for this study we analysed the data by hospital (private/public). A previous study published in 2000 [10] showed that there was a moderating factor on intervention rates when women with private insurance status gave birth in a public hospital, leading to lower intervention rates than when they gave birth in private hospitals.

The NSW PDC contains statistics on all births in New South Wales - which amounts to one third of all births which occur in Australia annually. Data is provided for maternal age, maternal hypertension, maternal diabetes, parity, private/public patient status, fetal presentation, onset

For peer review only - http://bmjopen.bmj.com/site/about/guidelines.xhtml

of labour, gestation at birth, delivery type, Apgar scores and admission to neonatal intensive care and resuscitation details for the neonate. This dataset (NSW PDC) was linked to the Admitted Patient Data Collection (APDC) for the time period July 1st 2000 – June 30th 2008. The APDC records all admitted patient services provided by NSW Public Hospitals, Public Psychiatric Hospitals, Public Multi-Purpose Services, Private Hospitals, and Private Day Procedures Centres. The APDC provided additional information, such as data on maternal medical conditions, which was used to exclude further maternal cases and was used to calculate admission and readmission details for neonates. Further linkage occurred to the NSW Registry of Births, Deaths and Marriages (RBDM) and the Australian Bureau of Statistics Death Data, which provided mortality data. The NSW Register of congenital conditions provided cases of congenital conditions, as did the coding in the APDC. Any neonate (and mother pair) with a recorded congenital condition (ICD-10-AM codes Q0.0-Q99.9) on either dataset was removed from the dataset due to their high-risk status. Probabilistic data linkage techniques were utilised for data linkage and de-identified datasets were provided for analysis. Probabilistic record linkage software assigns a 'linkage weight' to pairs of records. For example, records that match perfectly or nearly perfectly on first name, surname, date of birth and address have a high linkage weight, and records that match only on date of birth have a low linkage weight. If the linkage weight is high it is likely that the records truly match, and if the linkage weight is low it is likely that the records are not truly a match. This technique has been shown to have a false positive rate of 0.3% of records [12].

Gestation is recorded at birth and is also recorded in the database according to the woman's menstrual history, usually combined with a routine scan at 12-13 weeks.

Admission to neonatal intensive care refers to admission to special care nursery (SCN) or neonatal intensive care unit (NICU).

Any resuscitation includes suction of the mouth or nostrils at birth; oxygen administered by mask; intermittent positive pressure respiration (IPPR) by bag and mask or by intubation; external cardiac massage and ventilation.

Ethical approval was obtained from the NSW Population and Health Services Research Ethics Committee, Protocol No.2010/12/291.

For peer review only - http://bmjopen.bmj.com/site/about/guidelines.xhtml

For peer review only

Subjects

We classified the low risk primipara as a first time mother aged 20-34 years, who had no pre-existing or pregnancy related hypertension or diabetes, was a non-smoker, and gave birth at 37-41 completed weeks gestation to a singleton baby in a cephalic presentation within the 10th and 90th centiles for gestation and birth weight. The low risk multipara was a woman having her second or subsequent baby aged 20-34 years, who had no pre-existing or pregnancy related hypertension or diabetes, was a non-smoker, gave birth at 37-41 completed weeks gestation to a singleton baby in a cephalic presentation within the 10th and 90th centiles for gestation and birth weight. We excluded women with a previous caesarean section or who were induced for a medical indication, or who underwent a caesarean section for a pre-existing medical indication or gave birth without a trained birth attendant (born before arrival). If a caesarean section was undertaken during labour however for non-reassuring heart rate, dystocia etc these women were included in the study. These characteristics were defined firstly from the PDC with additional medical conditions identified in the APDC being used to exclude cases.

Outcomes

Any neonatal admission including the ICD-10-AM codes Z37.0 (single live birth), Z37.1 (single stillbirth) or Z38.0 (singleton born in hospital) was deemed the birth admission and any ICD-10-AM codes referring to conditions which arise in the perinatal period (P00-P96) and those referring to factors influencing health status and contact with health services (Z00-Z99) which were included in this admission were deemed morbidities associated with the birth admission. Any other admission following this discharge from the initial birth admission to home or another hospital was deemed a readmission and included transfers to a hospital other than that where the birth occurred. When examining readmission data, all ICD-10-AM codes recorded were reviewed and those where ≥10 events occurred in either private or public hospitals were marked for analysis. Events were grouped in body systems where appropriate or under headings such as infection for ease of analysis and interpretation.

For peer review only - http://bmjopen.bmj.com/site/about/guidelines.xhtml

Morbidity was recorded with the birth admission and rates of events were calculated using the number of babies who had any morbidity recorded with their birth.

Stillbirth and neonatal deaths were calculated from multiple sources but were limited to those that occurred within 28 days of birth and they were only counted once. Death may have been detected on any one of the following four datasets. The PDC 'Discharge status' variable or admissions in the APDC where the case mode separation was coded as 'Died' or the NSW RBDM or ABS Death Data where a death had been recorded. The maternal admission data for any admission that occurred during the pregnancy, as well as the birth admission for all cases of stillbirth or neonatal death were examined to determine any maternal medical or pregnancy related condition. This methodology of utilising multiple data sources to identify cases has been shown by Lain et al (2012) to be the most reliable way to increase ascertainment of cases [13].

Obstetric intervention was defined to include induction, epidural use, episiotomy, instrumental delivery (requiring the use of forceps or vacuum) and delivery via caesarean section.

Data analysis

The cohort was divided into primiparous and multiparous women for the primary analysis of birth outcomes. When examining neonatal status at birth mortality odds ratios were calculated using logistic regression with and without adjustment for age and gestation. For neonatal morbidity at birth and readmission, chi-square statistics were calculated for observed events. The number of babies born in a public or a private hospital were used as the denominator when calculating the percentage of babies born with a morbidity code attached to their birth record or the number of babies readmitted with a designated morbidity code. This methodology provides for comparison between place of birth taking into consideration the fact that up to 55 morbidity codes can be attached to any one birth or readmission record. Taking into account the size of the cohort and the number of analyses undertaken, results were considered significant at the level $p<0.01$. Analysis was undertaken with IBM SPSS v.20®

For peer review only - http://bmjopen.bmj.com/site/about/guidelines.xhtml

Results

Maternal characteristics, interventions and outcomes

The PDC dataset for the time period July 1st 2000 to 2nd June 2008 contained the antenatal, birth and postnatal details on 691 738 births. The APDC for the time period July 1st 2000 to 30th June 2008 contained >1.1 million admissions for the neonates/children of these women.

From the total population of primiparous women (288,309 women), 29,597 low risk primiparous women gave birth in private hospitals in NSW and 79,792 low risk primiparous women gave birth in public hospitals. The rates of obstetric intervention were much higher amongst those who gave birth in private hospitals compared to those who gave birth in public hospitals when all interventions for pre-specified medical reasons were removed. Low risk primiparous women giving birth in private hospitals had higher rates of induction for non-medical reasons (19% vs 7%), instrumental birth (30% vs 20%), caesarean section (25% vs 16%), epidural (71% vs 35%) and episiotomy (42% vs 23%). Severe perineal trauma (defined as third and fourth degree perineal trauma) was lower in a private hospital in first time mothers (4.7% vs 5.4%) (Table 1).

Amongst the total population of multiparous women (403,429 women), 28,703 low risk multiparous women gave birth in private hospitals and 99,212 low risk multiparous women gave birth in public hospitals The rate of obstetric intervention was significantly higher amongst those who gave birth in private hospitals in NSW compared to those who gave birth in public hospitals when all interventions for specific medical reasons were removed. Low risk multiparous women who gave birth in private hospitals had higher rates of induction for non- medical reasons (32% vs 10%), instrumental birth (8% vs 3%), epidural (34% vs 10%) and episiotomy (16% vs 5.%) and similar rates of severe perineal trauma (0.9%).

For peer review only - http://bmjopen.bmj.com/site/about/guidelines.xhtml

The caesarean section rate still remained higher in the private cohort (5.9% vs 4.5%) though this was mostly associated with elective caesarean section (Table 2).

Perinatal characteristics, interventions and outcomes

There was no difference in birth weight between babies born in a private and public hospital. Babies born in a private hospital were more likely to be born at 37, 38, 39 and 40 weeks and less likely to be born at 41 weeks gestation (Figure 1.).

Babies of primiparous women born in a private hospital were less likely to have an Apgar of <7 at five minutes (AOR 1.34 95% CI 1.77-1.53; p<0.001) or not to be resuscitated (AOR 0.36 95% CI 0.35-0.37; p<0.001). Babies born to low risk primiparous women in a private hospital were no more likely to be admitted to special care and/or neonatal intensive care (AOR 1.03 95% CI 0.98-1.08; p 0.210) and were less likely to have their baby transferred to another hospital (AOR 7.55 95% CI 6.52-8.74; p<0.001). There was no difference in the perinatal mortality rate for babies of primiparous women born in private or public hospitals (AOR 1.49 95% CI 0.93-2.41; p 0.10) (Table 3). Similar outcomes were seen for babies born to multiparous women in private and public hospitals however there was no difference in the incidence of Apgars of <7 at five minutes (Table 4).

Reason for birth admission of neonate

We examined neonatal morbidity as coded on the neonatal birth admission record and found fewer babies overall had a morbidity recorded (ICD-10-AM code other than the birth code) in the private sector compared to the public sector (40.0% vs 53.9%) due to the added clinical and social maternal complexity in the public sector. There were however some significant differences noted under the main ICD-10-AM Grouping *Certain conditions originating in the perinatal period (P00-P96)*. Babies born in a private hospital were more likely to have been affected by a forceps or vacuum delivery and were more likely to have trauma to the scalp (3.22% vs 2.22%), intrauterine hypoxia (1.70% vs 1.21%),

For peer review only - http://bmjopen.bmj.com/site/about/guidelines.xhtml

jaundice (4.68% vs 2.89%), minor cardiac murmurs (0.24% vs 0.17%), conjunctivitis (2.98% vs 1.27%), respiratory conditions (0.96% vs 0.57%), temperature regulation issues (2.08% vs 1.27%) feeding difficulties (3.83% vs 2.32%), carbohydrate metabolism issues (0.86% vs 0.53%), vomiting (0.55% vs 0.39%) and to be circumcised during the birth admission if a male (5.31% vs 0.21%)). Babies born in a public hospital were more likely to be admitted for observation and evaluation (6.35% vs 3.75%) have prophylactic antibiotics (0.52% vs 0.16%) and be admitted for socioeconomic circumstances (eg. housing, distance, adoption, assumption of care) (0.57% vs 0.05%) (Table 5)

Reason for birth re-admission of neonate up to 28 days of age

We examined the reasons for transfer or readmission of babies up until 28 days of age and found, that though the numbers are small, more babies born in private hospitals were readmitted compared to babies born in a public hospital (0.95% vs 0.65%) (Table 6). Babies born in private hospitals were more likely to be readmitted for infectious diseases (0.21% vs 0.12%) endocrine, nutritional and metabolic disorders (0.05% vs 0.02%), sleep disorders (0.03% vs 0.01%), hypoglycaemia (0.02% vs 0.01%), birth trauma such as cephalohaematoma (0.02% vs 0.01%); trauma involving the scalp (0.05% vs 0.02%), excessive crying (0.14% vs 0.07%), behavioural disorders (0.06% vs 0.02%) and for circumcision if a male (0.20 vs 0.13)). Babies born in public hospitals were more likely to be readmitted with respiratory disorders (0.27% vs 0.20%), injury and poisoning (eg burns) (0.05% vs 0.03%), antibiotic therapy (0.03% vs 0.01%) and socioeconomic circumstances (housing, distance, adoption, assumption of care (0.09% vs 0.04%).

Combined birth and readmission neonatal morbidity for selected codes

When we combined major birth and readmission morbidities for key selected codes we found that in the first 28 days following birth, babies born in private hospitals were significantly more likely to be admitted for feeding difficulties (4% vs 2.4%), circumcision if a male (5.6 vs 0.3)), birth trauma (mostly scalp trauma) (5% vs 3.6%), jaundice (4.8% vs 3.0%), hypoxia (1.7% vs 1.2%), respiratory disorders (1.2% vs 0.8%) and

sleep/behavioural issues (0.2% vs 0.1%). Babies born in public hospitals were more likely to be admitted for socioeconomic circumstances such as housing, distance, adoption or assumption of care (0.7% vs 0.1%) and prophylactic antibiotics (0.6% vs 0.2%) (Table 7 & Figure 2).

Discussion

Intervention rates

Despite being an extremely low risk cohort, less than half the primiparous women in this study giving birth in a private hospital had a normal vaginal birth (45% vs 65%); this was 20% lower than in the public cohort. One in five primiparous women giving birth in a private hospital were induced and nearly one in two had an episiotomy. For low risk multiparous women giving birth in a private hospital nearly one in three were induced. The trend for higher intervention rates has been reported for low risk women giving birth in the private sector in Australia previously and continues to show an increase [9, 14, 15]. In a recent publication we showed that the rate of caesarean section had increased in both the private and public sector in the past decade in low risk women [9]. It has been argued in a previous publication that these high intervention rates in the private sector led to better perinatal outcomes than in the public sector [2]. This publication received significant criticism in letters to the editor [3, 16, 17] for several methodological flaws, including most significantly the failure to adjust for low birth weight, inadequate ascertainment of congenital abnormalities and failure to look at perinatal morbidity. In this study we included only low risk women, adjusting for maternal age and gestational age differences. We also removed all babies with congenital abnormalities from this data set. We found that the perinatal mortality rate was not statistically different when the populations were matched in this data set for maternal risk.

Neonatal resuscitation and admission to SCN/NICU

We found that babies born in a private hospital were much more likely to experience some form of resuscitation, in particular twice the rate of suctioning at birth. Routine suctioning for infants born with clear and/or meconium stained amniotic fluid is not recommended [18] as it can cause a bradycardia [19] and there is no evidence of benefit. We are unsure why such a high rate of newborn suctioning continues in the

For peer review only - http://bmjopen.bmj.com/site/about/guidelines.xhtml

private sector. While rates of Apgar scores of ≤7 at five minutes were slightly higher amongst primiparous women who gave birth in public hospital (but not multiparous women), and this has been demonstrated in another recent Australian publication [15], overall the babies were no more likely to be admitted to SCN/NICU compared to babies born in private hospitals.

Neonatal admission and readmission

We found some interesting differences in morbidity however when examining morbidity attached to the birth admission and readmission to hospital in the first 28 days for codes that may be associated with the higher rates of obstetric intervention in the private sector and a different sociodemographic profile in the public sector. While increasingly preterm babies >35 weeks/ and or >2.2k and some cases of jaundice may be managed at the bedside in some hospitals, this is less likely to occur in a private hospital. It is more likely to occur in large maternity units.

Birth trauma, in particular injuries to the scalp, were significantly in the private sector and these are generally associated with instrumental birth, including vacuum extraction [20-22]. With more women (nearly one in three primiparous women) experiencing an instrumental birth in the private cohort and one in five women in the public sector this is not surprising. Birth trauma is associated with a longer hospital stay and increased risk of admission to SCN/NICU as well as higher rates of neonatal morbidity including neurological morbidity (hypotonia, jitteriness, convulsions and hypoxic ischaemic encephalopathy) and jaundice [22].

Jaundice was observed to be higher in the private sector, which may be related to several factors, such as the elective delivery of babies at an earlier gestation, the increased scalp trauma due to the high instrumental birth rate, as discussed above, and potential breastfeeding difficulties due to higher use of epidural analgesia. Jaundice has been associated with birth trauma, in particular delivery by vacuum extraction, and problems with feeding, especially supplementary feeding [22, 23]. Earlier gestational age <39 weeks has also be found to be associated with jaundice, with this decreasing with each week of additional gestation [24]. The gestational age of babies born in private hospitals in this

For peer review only - http://bmjopen.bmj.com/site/about/guidelines.xhtml

study was significantly lower than in the public sector due to the high rates of non-medical induction of labour and non-medically indicated caesarean section before the onset of labour.

While there have been studies associating difficulties with breastfeeding and higher rates of jaundice, the recent publication from the Universal Screening for Hyperbilirubinemia Study Group found difficulties with breastfeeding was a minimal risk factor [24].

Nearly twice as many babies who were born in a private hospital in this study were admitted or re-admitted with feeding problems compared to babies born in a public hospital. Feeding difficulties are associated with operative birth interventions and being early term [25, 26]. Breastfeeding outcomes are positively associated with uncomplicated unassisted vaginal birth where the mother and infant remain together and breastfeeding is started within an hour of the birth and following skin-to-skin contact. Interventions during labour and birth can impact on the initiation and duration of breastfeeding. Caesarean section [27-30], instrumental birth [31], epidural anaesthesia and opioid analgesia [32-34] use have all been associated with breastfeeding difficulties. All these birth interventions were higher in the private cohort in this study. In addition early term birth, which is mainly due to induction of labour and elective caesarean section (35, 36) is associated with increased breastfeeding difficulties along with other serious morbidities [37]. Unmedicated newborns are more highly aroused immediately following the birth [38] and able to breastfeed without assistance if given skin to skin contact and freedom from intrusive procedures [39, 40]. Following caesarean section there can be a significantly longer period of time until a mother touches and holds her newborn compared to an unassisted vaginal birth [41].

In a previous paper [6] using national Australian population data we found that among low-risk women who had an unassisted vaginal birth with spontaneous onset of labour and no labour augmentation, the odds of admission to neonatal intensive care or special care nursery were significantly increased when the baby was 37 weeks gestation at the time of birth compared to later gestations. Some claim that during the final weeks of gestation the fetal brain goes through a marked increase in mass and nerve growth (corticoneurogenesis) which may be best left

For peer review only - http://bmjopen.bmj.com/site/about/guidelines.xhtml

undisturbed by allowing the normal gestational length to occur [42]. In this study low risk women giving birth in private hospitals in NSW were much more likely to give birth at earlier gestations than their public hospital counterparts for every week up to and including 40 weeks, but they were significantly less likely to deliver at 41 weeks. This may also help to explain why more babies born in a private hospital were readmitted with, respiratory, feeding, jaundice and sleep and behavioural problems. However there is also evidence that there are increased adverse perinatal outcomes for babies born following 41 completed weeks, but we did not examine this population [43].

Circumcision

Babies born in a private hospital were significantly more likely to be circumcised in the first 28 days of life. This may be due to different information being given in private hospitals about the procedure or easy access to providers who perform the procedure. Circumcision rates are estimated to be between 10-20% in Australia [44] and are decreasing. A recent position statement of the Royal Australian College of Physicians states "that the frequency of diseases modifiable by circumcision, the level of protection offered by circumcision and the complication rates of circumcision do not warrant routine infant circumcision in Australia and New Zealand" [44].

Socioeconomic circumstances

The difference in the sociecononomic status of the women giving birth in public compared to private hospitals appears to be demonstrated by the significantly higher rates of public hospital babies with a morbidity attached to the birth admission or readmission in the first 28 days for socioeconomic circumstances, including housing, distance, adoption and assumption of care. This again confirms what is already known that the two populations are very different sociodemographically with greater disadvantage in the public sector.

For peer review only - http://bmjopen.bmj.com/site/about/guidelines.xhtml

Limitations

Our study is limited to providing a snapshot of perinatal outcomes in the most populous state in Australia in a defined time period for women who have no indicated risk at birth. However, this study provides useful data following on from our previous paper looking at obstetric intervention in private and public hospitals in NSW providing the reader with a detailed picture of perinatal mortality and morbidity. The advantages of using population-based datasets such as the PDC and the linkage to four other population-based databases include the size of the sample and the high level of accuracy of a validated dataset. The limitations are the restricted number of variables that are included and the scarcity of specific information on potential influencing variables. A small number of cases with a low linkage rate (0.3%) were not included and so there is the possibility of missing adverse outcomes. A previous study showed that where stillbirths are excluded due to low linkage these are at lower gestational ages and not term infants as were the focus in this study [45]. Previous validation studies have reported high levels of data accuracy for the majority of diagnoses and procedures conducted during labour and delivery in the state-wide data base [46, 47], although the recording of medical conditions and smoking are overall generally underreported [46, 48]. Having a linked data set provides a much richer picture than we have had previously of the morbidity and mortality associated with birth interventions. While we could not control for obesity due to lack of data, women who have private health insurance have lower rates of obesity and higher socioeconomic status, hence these health disadvantages are most likely over represented in the public women [49]. There are also several other socio-demographic factors we could not control for, such as education and income, that increase risk for the women giving birth in public hospitals. This study can only provide an overview of possible associations between obstetric interventions and neonatal outcomes and does not imply causality, which could be better obtained from prospective cohort studies.

For peer review only - http://bmjopen.bmj.com/site/about/guidelines.xhtml

Conclusion

The continual rise in obstetric intervention for low risk women in Australia may be contributing to increased morbidity for healthy women and babies and higher cost of health care. The fact that these procedures which were initially life-saving are now so commonplace and do not appear to be associated with improved rates of perinatal mortality or morbidity demands close review. Early term delivery and instrumental births may be associated with increased morbidity in neonates and this requires urgent attention.

For peer review only

For peer review only - http://bmjopen.bmj.com/site/about/guidelines.xhtml

Table 1. Maternal characteristics, interventions and outcomes for low risk primiparous women in NSW (2000-2008)

Low risk Primiparous women	Private Hospital (n=29597)	Public Hospital (n=79792)
**Maternal age (years)**		
20-24	6.2%	28.9%
25-29	39.9%	40.9%
30-34	53.9%	30.2%
Weeks gestation at delivery		
37	4.5%	4.3%
38	15.4%	11.8%
39	27.8%	25.0%
40	43.2%	39.7%
41	9.1%	19.2%
**Type of labour**		
Spontaneous	71.9%	89.9%
Induced	19.2%	7.1%
No labour	8.9%	3.0%
**Delivery**		
Normal vaginal	44.9%	64.8%

For peer review only

For peer review only - http://bmjopen.bmj.com/site/about/guidelines.xhtml

Forceps	11.5%	6.7%
Vacuum	18.9%	12.9%
Total caesarean section	24.7%	15.6%
Caesarean section (after labour)	15.9%	12.6%
Caesarean section before the onset of labour	8.8%	3.0%
**Epidural**	70.8%	35.4%
**Episiotomy**	42.4%	23.3%
**Severe perineal trauma**	4.7%	5.4%

For peer review only

For peer review only - http://bmjopen.bmj.com/site/about/guidelines.xhtml

Table 2. Maternal characteristics, interventions and outcomes for low risk multiparous women in NSW (2000-2008)

Low risk Multiparous women	Private Hospital (n=28703)	Public Hospital (n=99212)
**Maternal age (years)**		
20-24	2.1%	16.8%
25-29	25.8%	38.8%
30-34	72.1%	44.4%
Weeks gestation at delivery		
37	4.1%	4.0%
38	18.7%	13.0%
39	31.6%	26.9%
40	40.2%	40.8%
41	5.4%	15.3%
**Type of labour**		
Spontaneous	64.0%	87.4%
Induced	32.1%	10.1%
No labour	3.9%	2.5%
**Delivery**		
Normal vaginal	86.1%	92.7%

For peer review only - http://bmjopen.bmj.com/site/about/guidelines.xhtml

Forceps	1.9%	0.7%
Vacuum	6.1%	2.1%
Total caesarean section	5.9%	4.5%
Caesarean section after labour	2.0%	2.0%
Caesarean section before the onset of labour	3.9%	2.5%#
**Epidural**	34.4%	9.5%
**Episiotomy**	16.2%	5.1%
**Severe perineal trauma**	0.9%	0.9%

For peer review only

For peer review only - http://bmjopen.bmj.com/site/about/guidelines.xhtml

Figure 1. Comparison of gestation at delivery between all low risk women stratified by hospital type

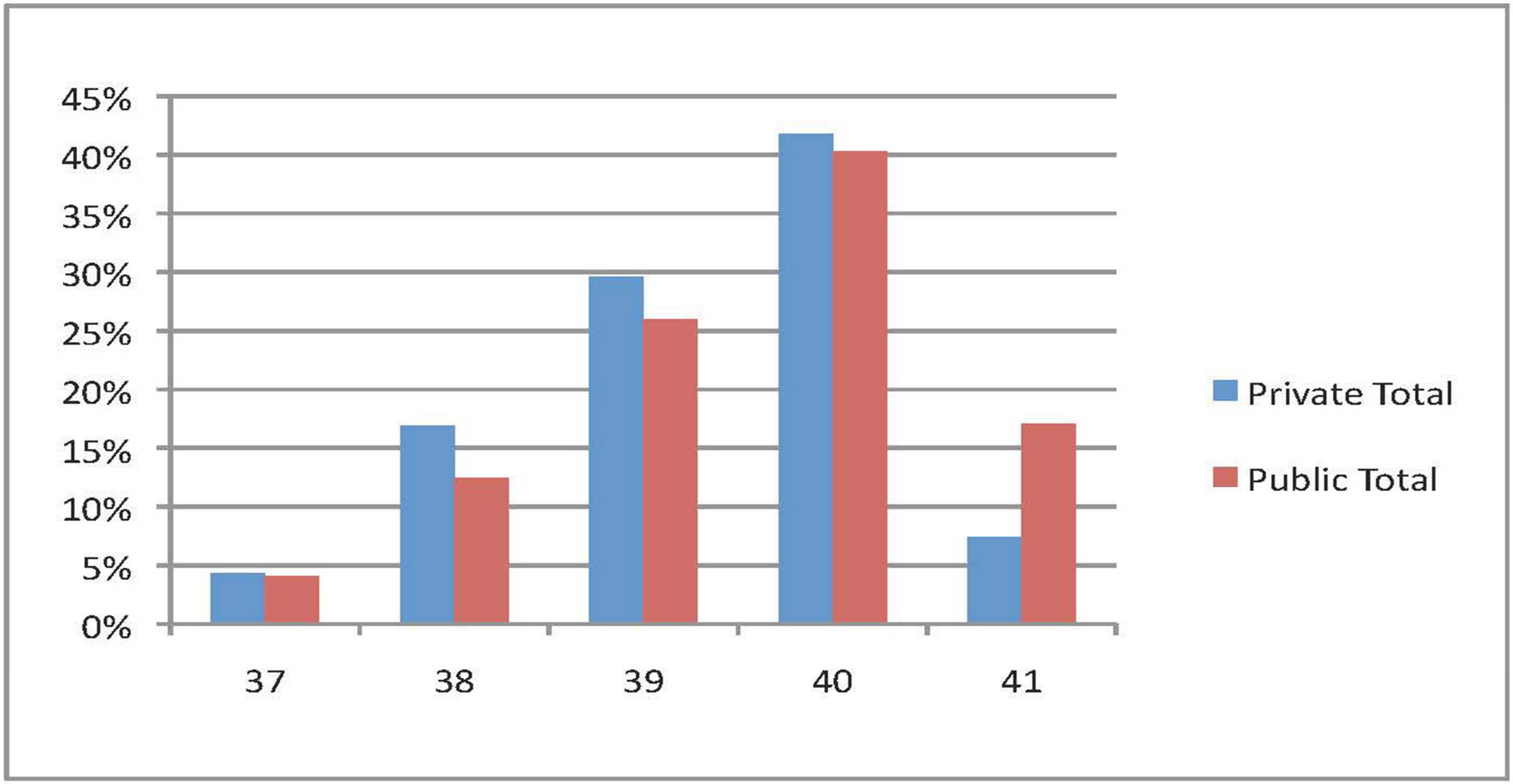

For peer review only - http://bmjopen.bmj.com/site/about/guidelines.xhtml

Table 3. Perinatal outcomes adjusted for maternal age and gestation at birth for low risk primiparous women

	Private	Public			
	(n=29597)	(n=79791)	OR**	AOR**	p
Apgar<7 at 5 minutes	296 (1.0%)	1037 (1.3%)	1.36 (1.12-1.54)	1.34(1.18-1.53)	<0.001
Any resuscitation*	18498 (62.5%)	30560 (38.3%)	0.372 (0.36-0.38)	0.364 (0.354-0.37)	<0.001
Admitted to SCN and/or NICU	3078 (10.4%)	8139 (10.2%)	1.00 (0.96-1.05)	1.03 (0.98-1.08)	0.210
Transferred	178 (0.6%)	3351 (4.2%)	7.30 (6.29-8.40)	7.55 (6.52-8.74)	<0.001
Total Perinatal Mortality	22 (0.74/1000)	85 (1.06/1000)	1.40 (0.93-2.01)	1.49 (0.93-2.41)	0.100

*Any resuscitation includes: Suction, oxygen, IPPR by bag and mask, Intubation and IPPR, external cardiac massage and ventilation and other

** Private hospital is the reference category

Table 4. Perinatal outcomes adjusted for maternal age and gestation at birth for low risk multiparous women

For peer review only - http://bmjopen.bmj.com/site/about/guidelines.xhtml

	Private	Public			
	(n=28703)	(n=99212)	OR**	AOR**	p
Apgar<7 at 5 minutes	67 (0.2%)	149 (0.2%)	1.56 (0.980-2.10)	1.42 (0.96-2.21)	0.562
Any resuscitation*	14820 (51.6%)	29867 (30.1%)	0.404 (0.39-0.42)	0.399 (0.39-0.46)	<0.001
Admitted to SCN and/or NICU	1775 (6.2%)	5870 (5.9%)	0.957 (0.91-1.01)	1.027 (0.97-1.09)	0.363
Transferred	232 (0.8%)	4375 (4.4%)	5.661 (4.96-6.47)	6.516 (5.70-7.45)	<0.001
Total Perinatal Mortality	17 (0.59/1000)	76 (0.77/1000)	1.294 (0.77-2.19)	1.294 (0.75-2.23)	0.355

*Any resuscitation includes: Suction, oxygen, IPPR by bag and mask, Intubation and IPPR, external cardiac massage and ventilation and other

** Private hospital is the reference category

For peer review only

Table 5. Morbidity associated with birth admission coded on neonatal birth admission record

		**Private**		**Public**		
**ICD-10-AM Grouping**		n=58300		n=179003		
**Certain conditions originating in the perinatal period (P00-P96)**		Count	%	Count	%	p
	Fetus and newborn affected by maternal infectious and parasitic diseases (P00.2)	41	0.07%	989	0.55%	<0.001
	Fetus and newborn affected by forceps delivery (P03.2)	473	0.81%	1108	0.62%	<0.001
	Fetus and newborn affected by delivery by vacuum extractor (P03.3)	511	0.88%	1509	0.84%	0.46
	**Birth trauma (all body systems) (P10-P15)**	**2948**	5.06%	**6447**	3.60%	<0.001
	- specifically to scalp (included in above total) (P12)	1880	3.22%	3965	2.22%	<0.001
	Intrauterine hypoxia (P20)	993	1.70%	2170	1.21%	<0.001
	Other specified respiratory conditions of newborn (P28)	562	0.96%	1015	0.57%	<0.001
	Benign and innocent cardiac murmurs in newborn (P29.82)	139	0.24%	303	0.17%	=0.001
	Neonatal conjunctivitis specific to the perinatal period (P39.1)	1740	2.98%	2267	1.27%	=0.001
	**Jaundice related conditions (P58-P59)**	**2728**	4.68%	**5166**	2.89%	<0.001
	Transitory disorders of carbohydrate metabolism specific to fetus and newborn (P70)	502	0.86%	942	0.53%	<0.001
	Conditions involving the integument & temperature regulation of fetus and newborn (P80-P83)	1214	2.08%	2275	1.27%	<0.001

For peer review only - http://bmjopen.bmj.com/site/about/guidelines.xhtml

	Vomiting in newborn (P92.0)	320	0.55%	693	0.39%	<0.001
	Feeding problems in newborn (P92)	2231	3.83%	4157	2.32%	<0.001
**factors influencing health status and contact with health services (Z00-Z99)**						
	Routine and ritual circumcision (Z41.2)*	1552	5.31%	187	0.21%	<0.001
	Observation and evaluation of newborn (Z03)	2187	3.75%	11372	6.35%	<0.001
	Prophylactic chemotherapy (antibiotics) (Z29.2)	93	0.16%	935	0.52%	<0.001
	Socioeconomic circumstances (housing, distance, adoption, assumption of care) (Z76)	32	0.05%	1020	0.57%	<0.001

* as a % of male babies

For peer review only - http://bmjopen.bmj.com/site/about/guidelines.xhtml

Table 6. Morbidity associated with readmission of the baby <28 days of age

		Private		Public		
		n=58300		n=179003		
**ICD-10-AM Grouping**		Count	%	Count	%	p
**Certain infectious and parasitic diseases (A00-B99)**		**121**	0.21%	**217**	0.12%	<0.001
**Endocrine nutritional and metabolic diseases (E00-E89)**		**30**	0.05%	**38**	0.02%	<0.001
	Volume depletion (E86)	18	0.03%	12	0.01%	<0.001
**Mental and behavioural disorders (F00-F99)**		**33**	0.06%	**30**	0.02%	<0.001
	Non-organic hypersomnia (F51.1)	17	0.03%	19	0.01%	=0.002
**Diseases of the nervous system (G00-G99)**		**34**	0.06%	**38**	0.02%	<0.001
	Disorders of the sleep wake schedule (G47.2)	18	0.03%	9	0.01%	<0.001
**Diseases of the ear and mastoid process (H60-H95)**		**19**	0.03%	**23**	0.01%	=0.002
**Diseases of the respiratory system (J00-J99)**		**155**	0.27%	**351**	0.20%	=0.002
	Acute obstructive laryngitis (croup) (J05)	6	0.01%	16	0.01%	=0.96
	Acute upper respiratory infection unspecified (J06)	16	0.03%	52	0.03%	=0.96

For peer review only - http://bmjopen.bmj.com/site/about/guidelines.xhtml

	Pneumonia (J10-J18)	6	0.01%	19	0.01%	=0.96
	Acute bronchiolitis (J21)	70	0.12%	175	0.10%	=0.17
	Unspecified acute lower respiratory tract infection (J22)	3	0.01%	12	0.01%	*
**Diseases of the digestive system (K00-K93)**		**53**	0.09%	**92**	0.05%	=0.001
	Gastro-oesophageal reflux disease (K21)	26	0.04%	37	0.02%	=0.003
**Certain conditions originating in the perinatal period (P00-P96)**		**474**	0.81%	**1011**	0.56%	<0.001
	Cephalohaematoma due to birth trauma (P12.0)	14	0.02%	13	0.01%	=0.002
	Total birth trauma to scalp (P12)	30	0.05%	32	0.02%	<0.001
	Intrauterine hypoxia (P20)	4	0.01%	25	0.01%	*
	Other neonatal hypoglycaemia (P70.4)	14	0.02%	14	0.01%	=0.002
	Other transitory neonatal electrolyte and metabolic disturbances (P70.8)	5	0.01%	21	0.01%	=0.527
	Fever of newborn (P81.9)	5	0.01%	18	0.01%	0.753
	Feeding problems of newborn (P92)	40	0.07%	100	0.06%	=0.271
	**Neonatal jaundice (P58)**	**193**	0.33%	**90**	0.05%	<0.001
**Symptoms, signs and abnormal findings not elsewhere classified (R00-R99)**		**198**	0.34%	**340**	0.19%	<0.001
	Fever (R50)	19	0.03%	27	0.02%	=0.008

For peer review only - http://bmjopen.bmj.com/site/about/guidelines.xhtml

	Feeding difficulties and mismanagement (R63.3)	25	0.04%	37	0.02%	=0.003
	Excessive crying (R68.1)	83	0.14%	117	0.07%	<0.001
**Injury, poisoning and certain other consequences of external causes (S00-T98)**		**15**	0.03%	**94**	0.05%	=0.009
	Burns (T20-T31)	0	0.00%	30	0.02%	*
**Factors influencing health status and contact with health services (Z00-Z99)**		**192**	0.33%	**691**	0.39%	=0.051
	Observation and evaluation in newborn (Z03)	34	0.06%	102	0.06%	=0.907
	Prophylactic chemotherapy (antibiotics) (Z29.2)	5	0.01%	49	0.03%	=0.009
	Routine and ritual circumcision (Z41.2)	68	0.20%	119	0.13%	<0.001
	Attention to surgical dressings and sutures (Z48.0)	0	0.00%	28	0.02%	*
	Socio-economic circumstances (housing, distance, adoption, assumption of care) (Z76)	22	0.04%	156	0.09%	<0.001

***cell size too small to calculate chi-square ** as a % of male babies**

For peer review only

Table 7. Combined birth and readmission neonatal morbidity for selected codes

	Private	Public	P*
Total feeding difficulties	2314 (4.0%)	4306 (2.4%)	<0.0001
Total circumcision**	1620 (5.6%)	306 (0.3%)	<0.0001
Total socio-economic circumstances	54 (0.1%)	1176 (0.7%)	<0.0001
Total birth trauma	2922 (5.0%)	6492 (3.6%)	<0.0001
Total hypoxia	997 (1.7%)	2195 (1.2%)	<0.0001
Total jaundice	2818 (4.8%)	5359 (3.0%)	<0.0001
Total respiratory	717 (1.2%)	1366 (0.8%)	<0.0001
Total sleep/behavioural issues	118 (0.2%)	145 (0.1%)	<0.0001
Prophylactic antibiotics	98 (0.2%)	982 (0.6%)	<0.001

*chi-square ** as a % of male babies

For peer review only

Figure 2. Birth trauma as a percentage of all births in Private and Public Hospitals

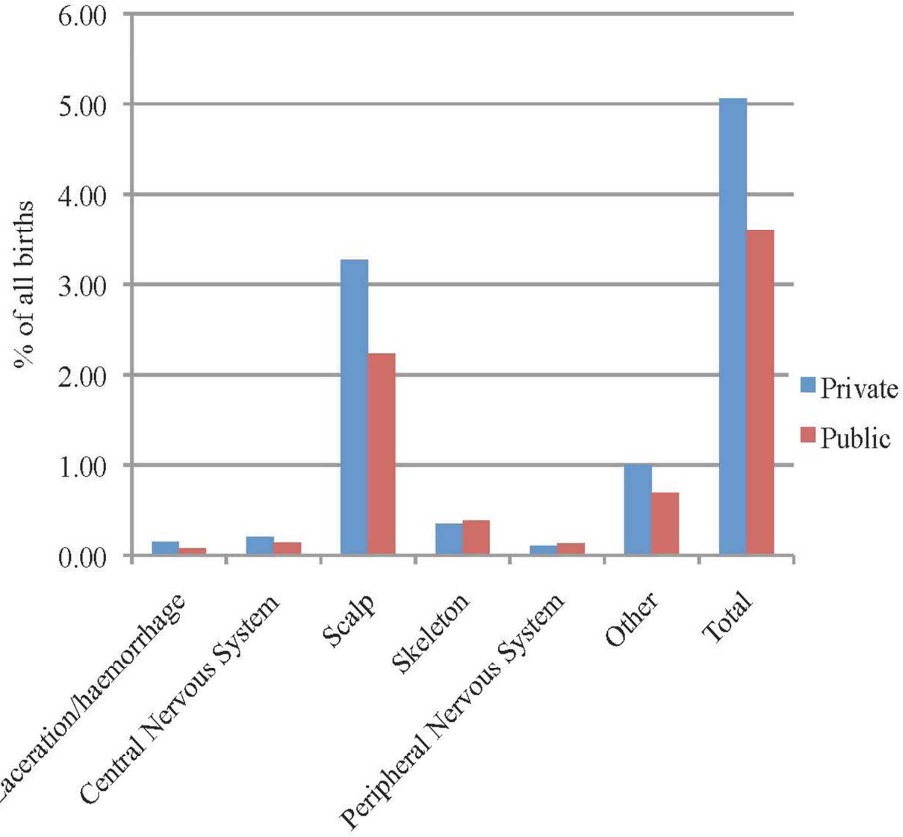

References

1 Li Z, Zeki R, Hilder L, Sullivan EA. Australia's mothers and babies 2011. Perinatal statistics series no. 28. Cat. no. PER 59. In: Unit ANPEaS, editor. Canberra2013.

2 Robson SJ, Laws P, Sullivan EA. Adverse outcomes of labour in public and private hospitals in Australia: a population based descriptive study. The Medical Journal of Australia 2009;190(9):474-7.

3 Tracy S, Welsh A, Dahlen H, Tracy M. Letter to the Editor re Robson SJ, Laws P, Sullivan EA. Adverse outcomes of labour in public and private hospitals in Australia: a population-based descriptive study. Med J Aust 2009; 190: 474-477. Medical Journal of Australia. 2009;191(10):579-80.

4 Clark E.A.S, Silver RM. Long-term maternal morbidity associated with repeat caesarean delivery. American Journal of Obstetrics and Gynecology. 2011;S2(December).

5 Hyde MJ, Mostyn A, Modi N, Kemp PR. The health implications of birth by caesarean section. Biological Reviews. 2012;87(1):229-43.

6 Tracy S, Tracy M, Sullivan E. Admission of Term Infants to Neonatal Intensive Care: A Population-Based Study. Birth 2007;34(4):301-7.

7 Tracy S, K , Tracy M. Costing the cascade: estimating the cost of increased obstetric intervention in childbirth using population data. British Journal of Obstetrics and Gynaecology. 2003;110:717-24.

8 Allen VM, O'Connell CM, Farrell SA, Baskett TF. Economic implications of method of delivery. Am J Obstet Gynecol 2005;193(1):192-7.

9 Dahlen HG, Tracy S, Tracy M, Bisits A, Brown C, Thornton C. Rates of obstetric intervention among low-risk women giving birth in private and public hospitals in NSW: a population-based descriptive study. BMJ Open. 2012;2:e001723 doi:10.1136/bmjopen-2012-001723.

10 Roberts C, L,, Tracy S, Peat B. Rates of obstetric intervention among private and public patients in Australia: population based descriptive study. British Medical Journal. 2000;312:137-41.

11 Buist R. Letter to the Editor: Private Obstetrics-again. BMJ Open. 2012;2(5):e001723 doi:10.1136/bmjopen-2012-.

12 CHeReL. Centre for Health Record Linkage (CHeReL). Quality Assurance Report 2012. http://wwwcherelorgau/media/24160/qa_report_2012pdf. 2012.

For peer review only

13 Lain SJ, Hadfield RM, Raynes-Greenow CH, Ford JB, Mealing NM, Algert CS, et al. Quality of data in perinatal population health databases: a systematic review. Med Care. 2012;50(4):e7-e20.

14 Roberts CL, Tracy S, Peat B. Rates for obstetric intervention among private and public patients in Australia: population based descriptive study. British Medical Journal. 2000 2000;321(7254):137-41.

15 Einarsdóttir K, Stock S, Haggar F, Hammond G, Langridge AT, Preen DB, et al. Neonatal complications in public and private patients: a retrospective cohort study. BMJ Open. 2013;Downloaded from bmjopen.bmj.com on November 11, 2013.

16 Evans N, Malcolm G, Gordon A. Letter to the Editor re Adverse outcomes of labour in public and private hospitals in Australia. MJA. 2009;191(10):579.

17 Chambers D. Letter to the Editor re Adverse outcomes of labour in public and private hospitals in Australia. MJA. 2009;191(10):578-9.

18 Wyllie J. Recent changes to UK newborn resuscitation guidelines. Arch Dis Child Fetal Neonatal Ed. 2012;97(1):F4-F7.

19 Kannapiran R, MKennea N. Resuscitation of the newborn. Obstetrics, Gynecology & Reproductive Medicine. 2012;22(4):92-7.

20 Doumouchtsis SK, Arulkumaran S. Head injuries after instrumental vaginal deliveries. Current Opion in Obstetrics and Gynaecology. 2006;18:129-34.

21 Towner D, Castro MA, Wilkens EE, Gilbert WM. Effect of Mode of Delivery in Nulliparous Women on Neonatal Intracranial Injury. The New England Journal of Medicine 1999;341:1709-14.

22 Linder N, Linder I, Fridman E, Kouadio F, Lubin D, Merlob P, et al. Birth trauma – risk factors and short-term neonatal outcome. The journal of Maternal-fetal & Neonatal Medicine. 2013;DOI: 10.3109/14767058.2013.789850.

23 Bertini G, Dani C, Tronchin M, Rubaltelli FF. Is breastfeeding really favouring early neonatal jaundice? Pediatrics. 2006;107(3):e41.

24 Bhutani VK, Stark AR, Lazzeroni LC, Poland R, Gourley GR, Kazmierczak S, et al. Predischarge Screening for Severe Neonatal Hyperbilirubinemia Identifies Infants Who Need Phototherapy. The Journal of Pediatrics. 2013;162(3):477-82.

25 Young PC, Korgenski K, Buchi KF. Early Readmission of Newborns in a Large Health Care System. Pediatrics. 2013;131(5):e1538-e44.

26 Brown A, Jordan S. Impact of birth complications on breastfeeding duration: an internet survey. Journal of Advanced Nursing. 2012;69(4):828-39.

27 Chapman D.J, Perez-Escamilla R. Identification of risk factors for delayed onset oflactation. Journal of the American Dietetic Association. 2003;99:450-4.

For peer review only

28 Dewey K.G., Nommsen-Rivers L.A., Heinig M.J, Cohen R.J. Risk factors for suboptimal infant breastfeeding behavior, delayed onset of lactation and excess neonatal weight loss. Pediatrics 2003;112(3):607-19.

29 Scott J.A., Binns C.W, Oddy W.H. Predictors of delayed onset of lactation. Maternal and Child Nutrition. 2007;3(3):186-93.

30 Jordan S., Emery S., Watkins A., Evans J.D., Storey M, Morgan G. Associations of drugs routinely given in labour with breastfeeding at 48 hours: analysis of the Cardiff Births Survey. British Journal of Gynecology. 2009;116(12):1622-9.

31 Leung G.M, Lam T.H, Ho L.M. Breast-feeding and its relation to smoking and mode of delivery. Obstetrics and Gynecology 2002;99(5):785-94.

32 Jordan S. Infant feeding and analgesia in labour: the evidence is accumulating. International Breastfeeding Journal Research. 2006;1(25):doi: 10.1186/746-4358-1-25.

33 Henderson J.J., Dickinson J.E., Evans S.F., McDonald S.J, M.J. P. Impact of intrapartum epidural analgesia on breast- feeding duration. Australian and New Zealand Journal of Obstetrics and Gynaecology. 2003;43(5):372–7.

34 Beilin Y., Bodian C., Weiser J., Sabera H., Ittamar A., Feierman D., et al. Effect of labor analgesia with and without fentanyl on infant breastfeeding: a prospective, randomized double-blind study. Anesthesiology. 2005;103(6):1211– 7.

35 Ohnsorg T, Schiff J. Preventing elective induction before 39 weeks. , 93(11), 44-46. Minnesota Medicine. 2010;93(11):44-6.

36 Oshiro BT, Henry E, Wilson J, Branch DW, Varner MW. Decreasing elective deliveries before 39 weeks of gestation in an integrated health care system. Obstetrics & Gynecology. 2009;113:804-11.

37 Reddy UM, Ko CW, Willinger M. “Early term” births (37-38 weeks) are associated with increased mortality. American Journal of Obstetrics & Gynecology. 2006;195(S202).

38 McLaughlin FJ, O’Connor S, Deni R. Infant state and behavior during the first postpartum hour. The Psychological Record 1981;31:455–8.

39 Widstrom A-M, Ransjo-Arvidson AB, Christensson K, Matthiesen A- S, Winberg J, Uvnas-Moberg K. Gastric suction in healthy newborn infants. Effects on circulation and developing feeding behaviour. Acta Paediatr. 1987;76:566–72.

40 Righard L. How do newborns find their mother’s breast? . Birth. 1995;22:174–5.

41 Fisher J, Astbury J, Smith A. Adverse psychological impact of opera- tive obstetric interventions: a prospective study. Aust NZ J Psychiatry 1997;31:728–38.

For peer review only

42 Adams-Chapman I. Insults to the developing brain and impact on neurodevelopmental outcome. Journal of Communication Disorders. Journal of Communication Disorders. 2009;42:256-62

43. Gülmezoglu AM, Crowther CA, Middleton P, Heatley E. Induction of labour for improving birth outcomes for women at or beyond term. *Cochrane Database of Systematic Reviews* 2012;Issue 6. Art.No.:CD004945. DOI: 10.1002/14651858.CD004945.pub3.

44. The Royal Australian College of Physicians. Circumcision of infant males. In: Physicians TRACo, editor. Sydney: The Royal Australasian College of Physicians; 2010.

45. Bentley JP, Ford JB, Taylor LK, Irvine KA, Roberts CL. Investigating linkage rates among probabilistic linked births and hospital records. BMC Medical Research Methodology. 12:149, 2012.

46. Taylor L, Travis S, Pym M, Olive E, Henderson-Smart D. How useful are hospital morbidity data for monitoring conditions occurring in the perinatal period? Australian & New Zealand Journal of Obstetrics & Gynaecology. 2005;45:36 –41.

47 Roberts C, Bell J, Ford J, Morris J. Monitoring the quality of maternity care: how well are labour and delivery events reported in population health data? . Paediatirc and Perinatal Epidemiology. 2008;23:144-52.

48 Thornton C, Makris A, Ogle R, Hennessy A. Generic obstetric database systems are unreliable for reporting the Hypertensive Disorders of Pregnancy. Australian and New Zealand Journal Obstetrics and Gynaecology. 2004;44(505-509).

49 Finkelstein A, Fiebelkorn IC, Wang G. National medical Spending attributable to overweight and obesity: How much and who's paying. Health Affairs, 2003 Project HOPE–The People-to-People Health Foundation, Inc., 219-226. 2003.

For peer review only

Figure 1. Comparison of gestation at delivery between all low risk women stratified by hospital type

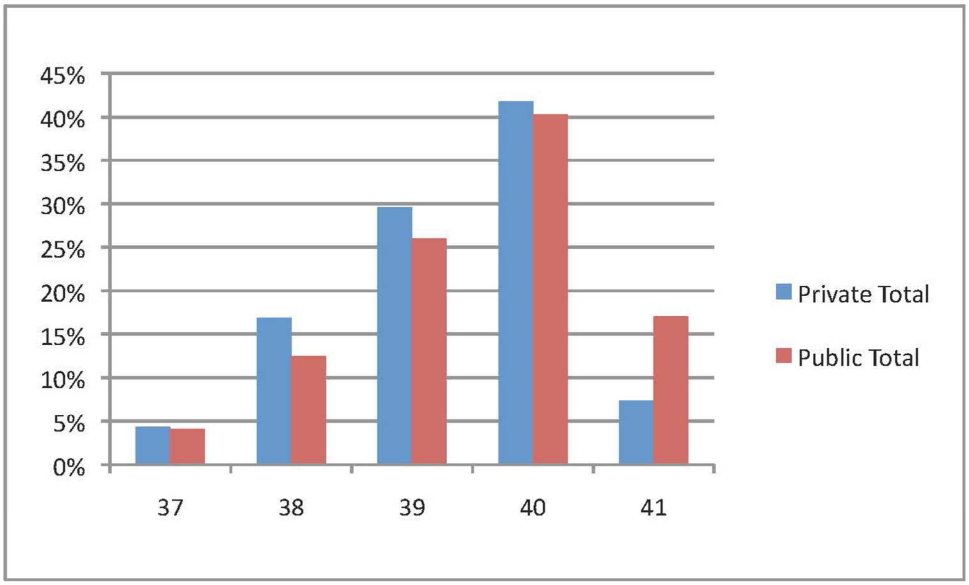

Figure 1: Comparison of gestation at delivery between all low risk women stratified bu hospital type.
118x91mm (300 x 300 DPI)

ew only

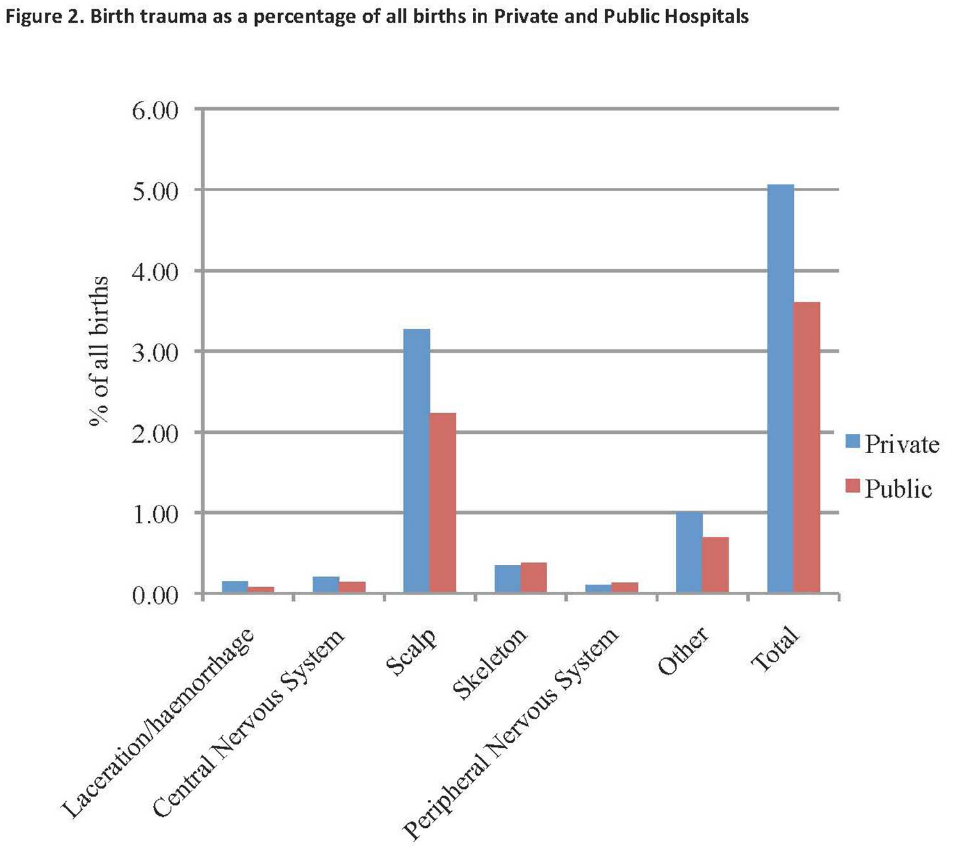

Figure 2: Birth trauma as a percentage of all births in Private and Public Hospitals.
116x100mm (300 x 300 DPI)

Rates of obstetric intervention and associated perinatal mortality and morbidity amongst low risk women giving birth in private and public hospitals in NSW (2000-2008): A linked data population based cohort study.

Journal:	*BMJ Open*
Manuscript ID:	bmjopen-2013-004551.R3
Article Type:	Research
Date Submitted by the Author:	29-Apr-2014
Complete List of Authors:	Dahlen, Hannah; University of Western Sydney, School of Nursing and Midwifery<br>Tracy, Sally; University of Sydney, Nursing and Midwifery<br>Tracy, Mark; University of Sydney, Medicine<br>Bisits, Andrew; Royal Hospital for Women, Royal Hopsital for Women<br>Brown, Chris; University of Sydney, NHMRC Clinical Trials Centre<br>Thornton, Charlene; University of Western Sydney, School of Nursing and Midwifery
**Primary Subject Heading**:	Obstetrics and gynaecology
Secondary Subject Heading:	Epidemiology, Health policy, Paediatrics
Keywords:	Health policy < HEALTH SERVICES ADMINISTRATION & MANAGEMENT, NEONATOLOGY, OBSTETRICS

For peer review only

Rates of obstetric intervention and associated perinatal mortality and morbidity amongst low risk women giving birth in private and public hospitals in NSW (2000-2008): A linked data population based cohort study.

Hannah G. Dahlen RN, RM, BN (Hons), MCommN, PhD, FACM

Associate Professor of Midwifery

School of Nursing and Midwifery

Family and Community Health Group

Locked Bag 1797

Penrith South DC

NSW

Australia

Ph: +61 2 96859118

h.dahlen@uws.edu.au

Sally Tracy

Professor Midwifery

Professor of Midwifery

University of Sydney,

Royal Hospital for Women, Sydney

Tel: 0420277106

For peer review only

For peer review only - http://bmjopen.bmj.com/site/about/guidelines.xhtml

Mark Tracy

Senior Newborn Intensive Care Specialist and HOD

CNBC Westmead Hospital

Senior Clinical Lecturer Paediatrics and Child Health

University of Sydney

Tel (02) 9845 6568

Andrew Bisits Mb bs(unsw)franzcog ,mmed stat ,dip clin epi

Acting Medical Co Director of Maternity, Lead Clinician Birthing Suite

Royal Hospital for Women

Conjoint Associate Professor School of Women and Children's Health University of NSW

Randwick NSW 2033

0419418492

Chris Brown, BSc, MBiostat

Biostatistician

NHMRC Clinical Trials Centre

Locked Bag 77

Camperdown NSW 1450

For peer review only - http://bmjopen.bmj.com/site/about/guidelines.xhtml

Charlene Thornton BN, Grad.Dip.Mid, M Sc Med (Research), M Sc Med (Clin Epi), PhD

Post Doctoral Research Fellow

School of Nursing and Midwifery

Family and Community Health Group

Locked Bag 1797

Penrith South DC

NSW

Australia

Keywords: low risk primiparous, low risk multiparous , induction, vaginal birth, obstetric care, caesarean, birth trauma

Word count 4596

Ethical approval was obtained from the NSW Population and Health Services Research Ethics Committee, Protocol No.2010/12/291.

For peer review only - http://bmjopen.bmj.com/site/about/guidelines.xhtml

Rates of obstetric intervention and associated perinatal mortality and morbidity amongst low risk women giving birth in private and public hospitals in NSW (2000-2008): A linked data population based cohort study.

Abstract

Objectives: To examine the rates of obstetric intervention and associated perinatal mortality and morbidity in the first 28 days amongst low risk women giving birth in private and public hospitals in NSW (2000-2008).

Design: Linked data population based retrospective cohort study involving five data sets.

Setting: New South Wales, Australia.

Participants: 691,738 women giving birth to a singleton baby during the period 2000 to 2008.

Main outcome measures: Rates of neonatal resuscitation, perinatal mortality, neonatal admission following birth and readmission to hospital in the first 28 days of life in public and private obstetric units.

Results: Rates of obstetric intervention amongst low risk women were higher in private hospitals, with primiparous women 20% less likely to have a normal vaginal birth compared to the public sector. Neonates born in private hospitals were more likely to be less than 40 weeks; more likely to have some form of resuscitation; less likely to have an Apgar <7 at five minutes. Neonates born in private hospitals to low risk mothers were more likely to have a morbidity attached to the birth admission and to be readmitted to hospital in the first 28 days for birth trauma (5% vs 3.6%); hypoxia (1.7% vs 1.2%) ; jaundice (4.8% vs 3%); feeding difficulties (4% vs 2.4%) ; sleep /behavioural issues (0.2% vs 0.1%); respiratory conditions (1.2% vs 0.8%) and circumcision (5.6 vs 0.3%) but they were less likely to be admitted for prophylactic antibiotics (0.2% vs 0.6%) and for socioeconomic circumstances (0.1% vs 0.7%). Rates of perinatal mortality were not statistically different between the two groups.

For peer review only - http://bmjopen.bmj.com/site/about/guidelines.xhtml

Conclusion:

For low risk women, care in a private hospital, which includes higher rates of intervention, appears to be associated with higher rates of morbidity seen in the neonate, and no evidence of a reduction in perinatal mortality

For peer review only

For peer review only - http://bmjopen.bmj.com/site/about/guidelines.xhtml

Article focus:

- To examine the rates of obstetric intervention and associated perinatal mortality and morbidity in the first 28 days amongst low risk women giving birth in private and public hospitals in NSW (2000-2008) using linked data.
- Rates of neonatal resuscitation, perinatal mortality, neonatal admission following birth and readmission to hospital in the first 28 days of life amongst those infants born in public and private obstetric units.

Key messages

- Babies born to low risk mothers in private hospitals are more likely to require some resuscitation compared to those born in public hospitals.
- For babies born in private hospitals to low risk mothers there are higher rates of morbidity such as birth trauma, hypoxia, jaundice, feeding difficulties, sleep/behavioural issues, respiratory conditions and circumcision. For babies born in public hospitals there are higher rates of antibiotic use and admission for socio-economic reasons (eg housing, distance, adoption and assumption of care)
- Rates of perinatal mortality were comparable between babies born to low risk women giving birth in private and public hospitals

Strengths and limitations of this study

- The strength of this study lies in the large sample size of linked birth data admissions associated with these births.
- The use of data from five population based datasets which have been linked to enhance validity and ascertainment
- Limitations are the restricted number of variables that are included and the scarcity of specific information on potential confounders.
- Body mass index and key sociodemographic risk factors could not be controlled for and this would have added risk to women giving birth in public hospitals.

For peer review only - http://bmjopen.bmj.com/site/about/guidelines.xhtml

Introduction

In Australia, the national statistics reveal that 29% (n =83,573) of women who gave birth in hospital gave birth in private hospitals directly under private obstetric care [1}. The remaining 71% (n=204,399) of women gave birth in public hospitals in Australia. Women who are privately insured have been reported to have better maternal and perinatal outcomes compared to women who give birth in public hospitals as public patients [2]; but it has been argued that these women tend to be less socioeconomically disadvantaged and healthier [3] and therefore might be expected to have better outcomes. Arguments about the impact of private status on health outcomes are in reality complex.

What is not disputed are the much higher rates of obstetric intervention that occur in private hospitals in Australia. At a national level, the intervention rates in childbirth, such as caesarean section, are significantly higher in the private sector (43.1% vs 28.4%) and the rates of normal vaginal birth significantly lower (42.7% vs 61%) [1]. Despite the rising intervention rates over the past decade, the perinatal mortality rate overall has not shown a corresponding decline [1]. There is also growing concern that the short and long term morbidity associated with major obstetric interventions, such as caesarean, may not be insignificant for the mother [4] and the baby [5, 6]. The cost to the tax payer of the rising intervention in childbirth is also significant [7, 8].

A recent study in New South Wales, Australia [9] found among 293,840 low-risk women, rates of obstetric intervention were highest in private hospitals and lowest in public hospitals. Low-risk primiparous women giving birth in a private hospital compared to a public hospital had higher rates of induction (31% vs 23%); instrumental birth (29% vs 18%); caesarean section (27% vs 18%), epidural (53% vs 32%), episiotomy (28% vs 12%) and lower normal vaginal birth rates (44% vs 64%). Low-risk multiparous women had higher rates of instrumental birth (7% vs 3%), caesarean section (27% vs 16%), epidural (35% vs 12%), episiotomy (8% vs 2%) and lower normal vaginal birth rates (66% vs 81%). Following a comparison with data from the decade previously [10], these interventions were found to have increased by 5% for women in public hospitals

For peer review only - http://bmjopen.bmj.com/site/about/guidelines.xhtml
For peer review only

and by over 10% for women in private hospitals [9]. Among low-risk primiparous women giving birth in private hospitals, 15 per 100 women had a vaginal birth with no obstetric intervention compared to 35 per 100 women giving birth in a public hospital [9]. Concern was expressed that perinatal mortality and morbidity was not reported in that paper [11]. In this study we aim to address this through examining the rates of obstetric intervention and associated perinatal mortality and morbidity attached to the birth admission and readmission to hospital in the first 28 days of life for low risk women giving birth in private and public hospitals in NSW (2000-2008).

Methods

Data Sources

The New South Wales Centre for Health Record Linkage conducted linkage of several datasets via the Health Record Linkage (CHeReL). Pregnancy and birth data for the time period July 1st 2000 till June 2nd 2008 of all singleton births were provided by New South Wales (NSW), Ministry of Health as recorded in the NSW Midwives Data Collection (MDC), now the Perinatal Data Collection (PDC). This population based surveillance system contains maternal and infant data on all births of greater than 400 grams birth weight and/or 20 completed weeks gestation. Hospitals are coded either as private or public in the data set. However, the data identifying women who received care in public hospitals under private accommodation status is no longer collected as it had been in the years 1996-97 and for this reason patients who are under private obstetric care in public hospitals are not able to be differentiated from their public counterparts, so for this study we analysed the data by hospital (private/public). A previous study published in 2000 [10] showed that there was a moderating factor on intervention rates when women with private insurance status gave birth in a public hospital, leading to lower intervention rates than when they gave birth in private hospitals.

The NSW PDC contains statistics on all births in New South Wales - which amounts to one third of all births which occur in Australia annually. Data is provided for maternal age, maternal hypertension, maternal diabetes, parity, private/public patient status, fetal presentation, onset

For peer review only - http://bmjopen.bmj.com/site/about/guidelines.xhtml

of labour, gestation at birth, delivery type, Apgar scores and admission to neonatal intensive care and resuscitation details for the neonate. This dataset (NSW PDC) was linked to the Admitted Patient Data Collection (APDC) for the time period July 1st 2000 – June 30th 2008. The APDC records all admitted patient services provided by NSW Public Hospitals, Public Psychiatric Hospitals, Public Multi-Purpose Services, Private Hospitals, and Private Day Procedures Centres. The APDC provided additional information, such as data on maternal medical conditions, which was used to exclude further maternal cases and was used to calculate admission and readmission details for neonates. Further linkage occurred to the NSW Registry of Births, Deaths and Marriages (RBDM) and the Australian Bureau of Statistics Death Data, which provided mortality data. The NSW Register of congenital conditions provided cases of congenital conditions, as did the coding in the APDC. Any neonate (and mother pair) with a recorded congenital condition (ICD-10-AM codes Q0.0-Q99.9) on either dataset was removed from the dataset due to their high-risk status. Probabilistic data linkage techniques were utilised for data linkage and de-identified datasets were provided for analysis. Probabilistic record linkage software assigns a 'linkage weight' to pairs of records. For example, records that match perfectly or nearly perfectly on first name, surname, date of birth and address have a high linkage weight, and records that match only on date of birth have a low linkage weight. If the linkage weight is high it is likely that the records truly match, and if the linkage weight is low it is likely that the records are not truly a match. This technique has been shown to have a false positive rate of 0.3% of records [12].

Gestation is recorded at birth and is also recorded in the database according to the woman's menstrual history, usually combined with a routine scan at 12-13 weeks.

Admission to neonatal intensive care refers to admission to special care nursery (SCN) or neonatal intensive care unit (NICU).

Any resuscitation includes suction of the mouth or nostrils at birth; oxygen administered by mask; intermittent positive pressure respiration (IPPR) by bag and mask or by intubation; external cardiac massage and ventilation.

Ethical approval was obtained from the NSW Population and Health Services Research Ethics Committee, Protocol No.2010/12/291.

For peer review only - http://bmjopen.bmj.com/site/about/guidelines.xhtml

Subjects

We classified the low risk primipara as a first time mother aged 20-34 years, who had no pre-existing or pregnancy related hypertension or diabetes, was a non-smoker, and gave birth at 37-41 completed weeks gestation to a singleton baby in a cephalic presentation within the 10th and 90th centiles for gestation and birth weight. The low risk multipara was a woman having her second or subsequent baby aged 20-34 years, who had no pre-existing or pregnancy related hypertension or diabetes, was a non-smoker, gave birth at 37-41 completed weeks gestation to a singleton baby in a cephalic presentation within the 10th and 90th centiles for gestation and birth weight. We excluded women with a previous caesarean section or who were induced for a medical indication, or who underwent a caesarean section for a pre-existing medical indication or gave birth without a trained birth attendant (born before arrival). If a caesarean section was undertaken during labour however for non-reassuring heart rate, dystocia etc these women were included in the study. These characteristics were defined firstly from the PDC with additional medical conditions identified in the APDC being used to exclude cases.

Outcomes

Any neonatal admission including the ICD-10-AM codes Z37.0 (single live birth), Z37.1 (single stillbirth) or Z38.0 (singleton born in hospital) was deemed the birth admission and any ICD-10-AM codes referring to conditions which arise in the perinatal period (P00-P96) and those referring to factors influencing health status and contact with health services (Z00-Z99) which were included in this admission were deemed morbidities associated with the birth admission. Any other admission following this discharge from the initial birth admission to home or another hospital was deemed a readmission and included transfers to a hospital other than that where the birth occurred. When examining readmission data, all ICD-10-AM codes recorded were reviewed and those where ≥10 events occurred in either private or public hospitals were marked for analysis. Events were grouped in body systems where appropriate or under headings such as infection for ease of analysis and interpretation.

For peer review only - http://bmjopen.bmj.com/site/about/guidelines.xhtml

For peer review only

Morbidity was recorded with the birth admission and rates of events were calculated using the number of babies who had any morbidity recorded with their birth.

Stillbirth and neonatal deaths were calculated from multiple sources but were limited to those that occurred within 28 days of birth and they were only counted once. Death may have been detected on any one of the following four datasets. The PDC 'Discharge status' variable or admissions in the APDC where the case mode separation was coded as 'Died' or the NSW RBDM or ABS Death Data where a death had been recorded. The maternal admission data for any admission that occurred during the pregnancy, as well as the birth admission for all cases of stillbirth or neonatal death were examined to determine any maternal medical or pregnancy related condition. This methodology of utilising multiple data sources to identify cases has been shown by Lain et al (2012) to be the most reliable way to increase ascertainment of cases [13].

Obstetric intervention was defined to include induction, epidural use, episiotomy, instrumental delivery (requiring the use of forceps or vacuum) and delivery via caesarean section.

Data analysis

The cohort was divided into primiparous and multiparous women for the primary analysis of birth outcomes. When examining neonatal status at birth mortality odds ratios were calculated using logistic regression with and without adjustment for age and gestation. For neonatal morbidity at birth and readmission, chi-square statistics were calculated for observed events. The total number of babies born in a public or a private hospital were used as the denominator when calculating the percentage of babies born with a morbidity code attached to their birth record or the number of babies readmitted with a designated morbidity code. This methodology provides for comparison between place of birth taking into consideration the fact that up to 55 morbidity codes can be attached to any one birth or readmission record. Taking into account the size of the cohort and the number of analyses undertaken, results were considered significant at the level $p<0.01$. Analysis was undertaken with IBM SPSS v.20®

For peer review only - http://bmjopen.bmj.com/site/about/guidelines.xhtml

Results

Maternal characteristics, interventions and outcomes

The PDC dataset for the time period July 1st 2000 to 2nd June 2008 contained the antenatal, birth and postnatal details on 691 738 births. The APDC for the time period July 1st 2000 to 30th June 2008 contained >1.1 million admissions for the neonates/children of these women.

From the total population of primiparous women (288,309 women), 29,597 low risk primiparous women gave birth in private hospitals in NSW and 79,792 low risk primiparous women gave birth in public hospitals. The rates of obstetric intervention were much higher amongst those who gave birth in private hospitals compared to those who gave birth in public hospitals when all interventions for pre-specified medical reasons were removed. Low risk primiparous women giving birth in private hospitals compared to low risk primiparous women giving birth in public hospitals had higher rates of induction for non-medical reasons (19% vs 7%), instrumental birth (30% vs 20%), caesarean section (25% vs 16%), epidural (71% vs 35%) and episiotomy (42% vs 23%). Severe perineal trauma (defined as third and fourth degree perineal trauma) was lower in a private hospital in first time mothers (4.7% vs 5.4%) (Table 1).

Amongst the total population of multiparous women (403,429 women), 28,703 low risk multiparous women gave birth in private hospitals and 99,212 low risk multiparous women gave birth in public hospitals The rate of obstetric intervention was significantly higher amongst those who gave birth in private hospitals in NSW compared to those who gave birth in public hospitals when all interventions for specific medical reasons were removed. Low risk multiparous women who gave birth in private hospitals compared to low risk primiparous women giving birth in public hospitals had higher rates of induction for non- medical reasons (32% vs 10%), instrumental birth (8% vs 3%), epidural (34% vs 10%) and episiotomy (16% vs 5.%) and similar rates of severe perineal trauma (0.9%). The caesarean section rate still remained higher in the private cohort (5.9% vs 4.5%) though this was mostly associated with elective caesarean section (Table 2).

For peer review only - http://bmjopen.bmj.com/site/about/guidelines.xhtml

Perinatal characteristics, interventions and outcomes

There was no difference in birth weight between babies born in a private and public hospital. Babies born in a private hospital were more likely to be born at 37, 38, 39 and 40 weeks and less likely to be born at 41 weeks gestation (Figure 1.).

Babies of primiparous women who gave birth in a private hospital were less likely to have an Apgar of <7 at five minutes (AOR 1.34 95% CI 1.18-1.53; p<0.001) as were babies of multiparous women who gave birth in private hospitals (AOR 1.37 95% CI 1.14-1.64). babies born in private hopsitals were less likely to have no resuscitation (AOR 0.36 95% CI 0.35-0.37; p<0.001). Babies born to low risk primiparous women in a private hospital were no more likely to be admitted to special care and/or neonatal intensive care (AOR 1.03 95% CI 0.98-1.08; p 0.210) and were less likely to have their baby transferred to another hospital (AOR 7.55 95% CI 6.52-8.74; p<0.001). There was no difference in the perinatal mortality rate for babies of primiparous women born in private or public hospitals (AOR 1.49 95% CI 0.93-2.41; p 0.10) (Table 3). Similar outcomes were seen for babies born to multiparous women in private and public hospitals however there was no difference in the incidence of Apgars of <7 at five minutes (Table 4).

Reason for birth admission of neonate

We examined neonatal morbidity as coded on the neonatal birth admission record and found fewer babies overall had a morbidity recorded (ICD-10-AM code other than the birth code) in the private sector compared to the public sector (40.0% vs 53.9%) due to the added clinical and social maternal complexity in the public sector. There were however some significant differences noted under the main ICD-10-AM Grouping *Certain conditions originating in the perinatal period (P00-P96)*. Babies born in a private hospital were more likely to have been affected by a forceps or vacuum delivery and were more likely to have trauma to the scalp (3.22% vs 2.22%), intrauterine hypoxia (1.70% vs 1.21%), jaundice (4.68% vs 2.89%), minor cardiac murmurs (0.24% vs 0.17%), conjunctivitis (2.98% vs 1.27%), respiratory conditions (0.96% vs 0.57%), temperature regulation issues (2.08% vs 1.27%) feeding difficulties (3.83% vs 2.32%), carbohydrate metabolism issues (0.86% vs 0.53%),

For peer review only - http://bmjopen.bmj.com/site/about/guidelines.xhtml

vomiting (0.55% vs 0.39%) and to be circumcised during the birth admission if a male (5.31% vs 0.21%)). Babies born in a public hospital were more likely to be admitted for observation and evaluation (6.35% vs 3.75%) have prophylactic antibiotics (0.52% vs 0.16%) and be admitted for socioeconomic circumstances (eg. housing, distance, adoption, assumption of care) (0.57% vs 0.05%) (Table 5)

Reason for birth re-admission of neonate up to 28 days of age

We examined the reasons for transfer or readmission of babies up until 28 days of age and found, that though the numbers are small, more babies born in private hospitals were readmitted compared to babies born in a public hospital (0.95% vs 0.65%) (Table 6). Babies born in private hospitals were more likely to be readmitted for infectious diseases (0.21% vs 0.12%) endocrine, nutritional and metabolic disorders (0.05% vs 0.02%), sleep disorders (0.03% vs 0.01%), hypoglycaemia (0.02% vs 0.01%), birth trauma such as cephalohaematoma (0.02% vs 0.01%); trauma involving the scalp (0.05% vs 0.02%), excessive crying (0.14% vs 0.07%), behavioural disorders (0.06% vs 0.02%) and for circumcision if a male (0.20 vs 0.13)). Babies born in public hospitals were more likely to be readmitted with respiratory disorders (0.27% vs 0.20%), injury and poisoning (eg burns) (0.05% vs 0.03%), antibiotic therapy (0.03% vs 0.01%) and socioeconomic circumstances (housing, distance, adoption, assumption of care (0.09% vs 0.04%).

Combined birth and readmission neonatal morbidity for selected codes

When we combined major birth and readmission morbidities for key selected codes we found that in the first 28 days following birth, babies born in private hospitals were significantly more likely to be admitted for feeding difficulties (4% vs 2.4%), circumcision if a male (5.6 vs 0.3)), birth trauma (mostly scalp trauma) (5% vs 3.6%), jaundice (4.8% vs 3.0%), hypoxia (1.7% vs 1.2%), respiratory disorders (1.2% vs 0.8%) and sleep/behavioural issues (0.2% vs 0.1%). Babies born in public hospitals were more likely to be admitted for socioeconomic circumstances such as housing, distance, adoption or assumption of care (0.7% vs 0.1%) and prophylactic antibiotics (0.6% vs 0.2%) (Table 7 & Figure 2).

For peer review only - http://bmjopen.bmj.com/site/about/guidelines.xhtml

For peer review only

Discussion

Intervention rates

Despite being an extremely low risk cohort, less than half the primiparous women in this study giving birth in a private hospital had a normal vaginal birth (45% vs 65%); this was 20% lower than in the public cohort. One in five primiparous women giving birth in a private hospital were induced and nearly one in two had an episiotomy. For low risk multiparous women giving birth in a private hospital nearly one in three were induced. The trend for higher intervention rates has been reported for low risk women giving birth in the private sector in Australia previously and continues to show an increase [9, 14, 15]. In a recent publication we showed that the rate of caesarean section had increased in both the private and public sector in the past decade in low risk women [9]. It has been argued in a previous publication that these high intervention rates in the private sector led to better perinatal outcomes than in the public sector [2]. This publication received significant criticism in letters to the editor [3, 16, 17] for several methodological flaws, including most significantly the failure to adjust for low birth weight, inadequate ascertainment of congenital abnormalities and failure to look at perinatal morbidity. In this study we included only low risk women, adjusting for maternal age and gestational age differences. We also removed all babies with congenital abnormalities from this data set. We found that the perinatal mortality rate was not statistically different when the populations were matched in this data set for maternal risk.

Neonatal resuscitation and admission to SCN/NICU

We found that babies born in a private hospital were much more likely to experience some form of resuscitation, in particular twice the rate of suctioning at birth. Routine suctioning for infants born with clear and/or meconium stained amniotic fluid is not recommended [18] as it can cause a bradycardia [19] and there is no evidence of benefit. We are unsure why such a high rate of newborn suctioning continues in the private sector. Rates of Apgar scores of $\leq$7 at five minutes were slightly higher amongst low risk women who gave birth in public hospital, and

For peer review only - http://bmjopen.bmj.com/site/about/guidelines.xhtml

this has been demonstrated in another recent Australian publication [15], overall the babies were no more likely to be admitted to SCN/NICU compared to babies born in private hospitals.

Neonatal admission and readmission

We found some interesting differences in morbidity however when examining morbidity attached to the birth admission and readmission to hospital in the first 28 days for codes that may be associated with the higher rates of obstetric intervention in the private sector and a different sociodemographic profile in the public sector. While increasingly preterm babies >35 weeks/ and or >2.2k and some cases of jaundice may be managed at the bedside in some hospitals, this is less likely to occur in a private hospital. It is more likely to occur in large maternity units.

Birth trauma, in particular injuries to the scalp, were significantly more common in the private sector and these are generally associated with instrumental birth, including vacuum extraction [20-22]. With more women (nearly one in three primiparous women) experiencing an instrumental birth in the private cohort and one in five women in the public sector this is not surprising. Birth trauma is associated with a longer hospital stay and increased risk of admission to SCN/NICU as well as higher rates of neonatal morbidity including neurological morbidity (hypotonia, jitteriness, convulsions and hypoxic ischaemic encephalopathy) and jaundice [22].

Jaundice was observed to be higher in the private sector, which may be related to several factors, such as the elective delivery of babies at an earlier gestation, the increased scalp trauma due to the high instrumental birth rate, as discussed above, and potential breastfeeding difficulties due to higher use of epidural analgesia. Jaundice has been associated with birth trauma, in particular delivery by vacuum extraction, and problems with feeding, especially supplementary feeding [22, 23]. Earlier gestational age <39 weeks has also be found to be associated with jaundice, with this decreasing with each week of additional gestation [24]. The gestational age of babies born in private hospitals in this

For peer review only - http://bmjopen.bmj.com/site/about/guidelines.xhtml

study was significantly lower than in the public sector possibly due to the high rates of non-medical induction of labour and non-medically indicated caesarean section before the onset of labour.

While there have been studies associating difficulties with breastfeeding and higher rates of jaundice, the recent publication from the Universal Screening for Hyperbilirubinemia Study Group found difficulties with breastfeeding was a minimal risk factor [24].

Nearly twice as many babies who were born in a private hospital in this study were admitted or re-admitted with feeding problems compared to babies born in a public hospital. Feeding difficulties are associated with operative birth interventions and being early term [25, 26]. Breastfeeding outcomes are positively associated with uncomplicated unassisted vaginal birth where the mother and infant remain together and breastfeeding is started within an hour of the birth and following skin-to-skin contact. Interventions during labour and birth can impact on the initiation and duration of breastfeeding. Caesarean section [27-30], instrumental birth [31], epidural anaesthesia and opioid analgesia [32-34] use have all been associated with breastfeeding difficulties. All these birth interventions were higher in the private cohort in this study. In addition early term birth, which is mainly due to induction of labour and elective caesarean section (35, 36) is associated with increased breastfeeding difficulties along with other serious morbidities [37]. Unmedicated newborns are more highly aroused immediately following the birth [38] and able to breastfeed without assistance if given skin to skin contact and freedom from intrusive procedures [39, 40]. Following caesarean section there can be a significantly longer period of time until a mother touches and holds her newborn compared to an unassisted vaginal birth [41].

In a previous paper [6] using national Australian population data we found that among low-risk women who had an unassisted vaginal birth with spontaneous onset of labour and no labour augmentation, the odds of admission to neonatal intensive care or special care nursery were significantly increased when the baby was 37 weeks gestation at the time of birth compared to later gestations. Some claim that during the final weeks of gestation the fetal brain goes through a marked increase in mass and nerve growth (corticoneurogenesis) which may be best left

For peer review only - http://bmjopen.bmj.com/site/about/guidelines.xhtml

undisturbed by allowing the normal gestational length to occur [42]. In this study low risk women giving birth in private hospitals in NSW were much more likely to give birth at earlier gestations than their public hospital counterparts for every week up to and including 40 weeks, but they were significantly less likely to deliver at 41 weeks. This may also help to explain why more babies born in a private hospital were readmitted with, respiratory, feeding, jaundice and sleep and behavioural problems. However there is also evidence that there are increased adverse perinatal outcomes for babies born following 41 completed weeks, but we did not examine this population [43].

Circumcision

Babies born in a private hospital were significantly more likely to be circumcised in the first 28 days of life. This may be due to different information being given in private hospitals about the procedure or easy access to providers who perform the procedure. Circumcision rates are estimated to be between 10-20% in Australia [44] and are decreasing. A recent position statement of the Royal Australian College of Physicians states “that the frequency of diseases modifiable by circumcision, the level of protection offered by circumcision and the complication rates of circumcision do not warrant routine infant circumcision in Australia and New Zealand” [44].

Socioeconomic circumstances

The difference in the sociocononomic status of the women giving birth in public compared to private hospitals appears to be demonstrated by the significantly higher rates of public hospital babies with a morbidity attached to the birth admission or readmission in the first 28 days for socioeconomic circumstances, including housing, distance, adoption and assumption of care. This again confirms what is already known that the two populations are very different sociodemographically with greater disadvantage in the public sector.

For peer review only - http://bmjopen.bmj.com/site/about/guidelines.xhtml

For peer review only

Limitations

Our study is limited to providing a snapshot of perinatal outcomes in the most populous state in Australia in a defined time period for women who have no indicated risk at birth. However, this study provides useful data following on from our previous paper looking at obstetric intervention in private and public hospitals in NSW providing the reader with a detailed picture of perinatal mortality and morbidity. The advantages of using population-based datasets such as the PDC and the linkage to four other population-based databases include the size of the sample and the high level of accuracy of a validated dataset. The limitations are the restricted number of variables that are included and the scarcity of specific information on potential influencing variables. A small number of cases with a low linkage (false/positive) rate (0.3%) were not included and so there is the possibility of missing adverse outcomes. A previous study showed that where stillbirths are excluded due to low linkage these are at lower gestational ages and not term infants as were the focus in this study [45]. Previous validation studies have reported high levels of data accuracy for the majority of diagnoses and procedures conducted during labour and delivery in the state-wide data base [46, 47], although the recording of medical conditions and smoking are overall generally underreported [46, 48]. Having a linked data set provides a much richer picture than we have had previously of the morbidity and mortality associated with birth interventions. While we could not control for obesity due to lack of data, women who have private health insurance have lower rates of obesity and higher socioeconomic status, hence these health disadvantages are most likely over represented in the public women [49]. There are also several other socio-demographic factors we could not control for, such as education and income, that increase risk for the women giving birth in public hospitals. This study can only provide an overview of possible associations between obstetric interventions and neonatal outcomes and does not imply causality, which could be better obtained from prospective cohort studies.

For peer review only - http://bmjopen.bmj.com/site/about/guidelines.xhtml

Conclusion

The continual rise in obstetric intervention for low risk women in Australia, especially in private hospitals, may be contributing to increased morbidity for healthy women and babies and higher cost of health care. The fact that these procedures which were initially life-saving are now so commonplace and do not appear to be associated with improved rates of perinatal mortality or morbidity demands close review. Early term delivery and instrumental births may be associated with increased morbidity in neonates and this requires urgent attention. Previous claims that high intervention rates in private hospitals lead to better perinatal outcomes than those seen in public hospitals need to be questioned.

For peer review only

Table 1. Maternal characteristics, interventions and outcomes for low risk primiparous women in NSW (2000-2008)

**Low risk Primiparous women**	**Private Hospital (n=29597)**	**Public Hospital (n=79792)**
**Maternal age (years)**		
20-24	6.2%	28.9%
25-29	39.9%	40.9%
30-34	53.9%	30.2%
Weeks gestation at delivery		
37	4.5%	4.3%
38	15.4%	11.8%
39	27.8%	25.0%
40	43.2%	39.7%
41	9.1%	19.2%
**Type of labour**		
Spontaneous	71.9%	89.9%
Induced	19.2%	7.1%
No labour	8.9%	3.0%
**Delivery**		
Normal vaginal	44.9%	64.8%

For peer review only - http://bmjopen.bmj.com/site/about/guidelines.xhtml

Forceps	11.5%	6.7%
Vacuum	18.9%	12.9%
Total caesarean section	24.7%	15.6%
Caesarean section (after labour)	15.9%	12.6%
Caesarean section before the onset of labour	8.8%	3.0%
**Epidural**	70.8%	35.4%
**Episiotomy**	42.4%	23.3%
**Severe perineal trauma**	4.7%	5.4%

For peer review only

For peer review only - http://bmjopen.bmj.com/site/about/guidelines.xhtml

Table 2. Maternal characteristics, interventions and outcomes for low risk multiparous women in NSW (2000-2008)

**Low risk Multiparous women**	**Private Hospital (n=28703)**	**Public Hospital (n=99212)**
**Maternal age (years)**		
20-24	2.1%	16.8%
25-29	25.8%	38.8%
30-34	72.1%	44.4%
Weeks gestation at delivery		
37	4.1%	4.0%
38	18.7%	13.0%
39	31.6%	26.9%
40	40.2%	40.8%
41	5.4%	15.3%
**Type of labour**		
Spontaneous	64.0%	87.4%
Induced	32.1%	10.1%
No labour	3.9%	2.5%
**Delivery**		
Normal vaginal	86.1%	92.7%

For peer review only - http://bmjopen.bmj.com/site/about/guidelines.xhtml

Forceps	1.9%	0.7%
Vacuum	6.1%	2.1%
Total caesarean section	5.9%	4.5%
Caesarean section after labour	2.0%	2.0%
Caesarean section before the onset of labour	3.9%	2.5%
**Epidural**	34.4%	9.5%
**Episiotomy**	16.2%	5.1%
**Severe perineal trauma**	0.9%	0.9%

For peer review only

For peer review only - http://bmjopen.bmj.com/site/about/guidelines.xhtml

Table 3. Perinatal outcomes adjusted for maternal age and gestation at birth for low risk primiparous women

	Private	Public			
	(n=29597)	(n=79791)	OR**	AOR**	p
Apgar<7 at 5 minutes	296 (1.0%)	1037 (1.3%)	1.36 (1.12-1.54)	1.34(1.18-1.53)	<0.001
Any resuscitation*	18498 (62.5%)	30560 (38.3%)	0.372 (0.36-0.38)	0.364 (0.36-0.37)	<0.001
Admitted to SCN and/or NICU	3078 (10.4%)	8139 (10.2%)	1.00 (0.96-1.05)	1.03 (0.98-1.08)	0.210
Transferred	178 (0.6%)	3351 (4.2%)	7.30 (6.29-8.40)	7.55 (6.52-8.74)	<0.001
Total Perinatal Mortality	22 (0.74/1000)	85 (1.06/1000)	1.40 (0.93-2.01)	1.49 (0.93-2.41)	0.100

*Any resuscitation includes: Suction, oxygen, IPPR by bag and mask, Intubation and IPPR, external cardiac massage and ventilation and other

** Private hospital is the reference category

For peer review only - http://bmjopen.bmj.com/site/about/guidelines.xhtml

Table 4. Perinatal outcomes adjusted for maternal age and gestation at birth for low risk multiparous women

	Private	Public			
	(n=28703)	(n=99212)	OR**	AOR**	p
Apgar<7 at 5 minutes	149 (0.5%)	676 (0.7%)	1.32 (1.10-1.57)	1.37 (1.14-1.64)	0.001
Any resuscitation*	14820 (51.6%)	29867 (30.1%)	0.404 (0.39-0.42)	0.399 (0.39-0.46)	<0.001
Admitted to SCN and/or NICU	1775 (6.2%)	5870 (5.9%)	0.957 (0.91-1.01)	1.027 (0.97-1.09)	0.363
Transferred	232 (0.8%)	4375 (4.4%)	5.661 (4.96-6.47)	6.516 (5.70-7.45)	<0.001
Total Perinatal Mortality	17 (0.59/1000)	76 (0.77/1000)	1.294 (0.77-2.19)	1.294 (0.75-2.23)	0.355

*Any resuscitation includes: Suction, oxygen, IPPR by bag and mask, Intubation and IPPR, external cardiac massage and ventilation and other

** Private hospital is the reference category

For peer review only

For peer review only

Table 5. Morbidity associated with birth admission coded on neonatal birth admission record

		Private		Public		
**ICD-10-AM Grouping**		n=58300		n=179003		
**Certain conditions originating in the perinatal period (P00-P96)**		Count	%	Count	%	p
	Fetus and newborn affected by maternal infectious and parasitic diseases (P00.2)	41	0.07%	989	0.55%	<0.001
	Fetus and newborn affected by forceps delivery (P03.2)	473	0.81%	1108	0.62%	<0.001
	Fetus and newborn affected by delivery by vacuum extractor (P03.3)	511	0.88%	1509	0.84%	0.46
	**Birth trauma (all body systems) (P10-P15)**	**2948**	5.06%	**6447**	3.60%	<0.001
	- specifically to scalp (included in above total) (P12)	1880	3.22%	3965	2.22%	<0.001
	Intrauterine hypoxia (P20)	993	1.70%	2170	1.21%	<0.001
	Other specified respiratory conditions of newborn (P28)	562	0.96%	1015	0.57%	<0.001
	Benign and innocent cardiac murmurs in newborn (P29.82)	139	0.24%	303	0.17%	=0.001
	Neonatal conjunctivitis specific to the perinatal period (P39.1)	1740	2.98%	2267	1.27%	=0.001
	**Jaundice related conditions (P58-P59)**	**2728**	4.68%	**5166**	2.89%	<0.001

	Transitory disorders of carbohydrate metabolism specific to fetus and newborn (P70)	502	0.86%	942	0.53%	<0.001
	Conditions involving the integument & temperature regulation of fetus and newborn (P80-P83)	1214	2.08%	2275	1.27%	<0.001
	Vomiting in newborn (P92.0)	320	0.55%	693	0.39%	<0.001
	Feeding problems in newborn (P92)	2231	3.83%	4157	2.32%	<0.001
**factors influencing health status and contact with health services (Z00-Z99)**						
	Routine and ritual circumcision (Z41.2)*	1552	5.31%	187	0.21%	<0.001
	Observation and evaluation of newborn (Z03)	2187	3.75%	11372	6.35%	<0.001
	Prophylactic chemotherapy (antibiotics) (Z29.2)	93	0.16%	935	0.52%	<0.001
	Socioeconomic circumstances (housing, distance, adoption, assumption of care) (Z76)	32	0.05%	1020	0.57%	<0.001

* as a % of male babies

For peer review only

For peer review only - http://bmjopen.bmj.com/site/about/guidelines.xhtml

Table 6. Morbidity associated with readmission of the baby ≤28 days of age

		**Private**		**Public**		
		n=58300		n=179003		
**ICD-10-AM Grouping**		Count	%	Count	%	**p**
**Certain infectious and parasitic diseases (A00-B99)**		**121**	0.21%	**217**	0.12%	<0.001
**Endocrine nutritional and metabolic diseases (E00-E89)**		**30**	0.05%	**38**	0.02%	<0.001
	Volume depletion (E86)	18	0.03%	12	0.01%	<0.001
**Mental and behavioural disorders (F00-F99)**		**33**	0.06%	**30**	0.02%	<0.001
	Non-organic hypersomnia (F51.1)	17	0.03%	19	0.01%	=0.002
**Diseases of the nervous system (G00-G99)**		**34**	0.06%	**38**	0.02%	<0.001
	Disorders of the sleep wake schedule (G47.2)	18	0.03%	9	0.01%	<0.001
**Diseases of the ear and mastoid process (H60-H95)**		**19**	0.03%	**23**	0.01%	=0.002
**Diseases of the respiratory system (J00-J99)**		**155**	0.27%	**351**	0.20%	=0.002
	Acute obstructive laryngitis (croup) (J05)	6	0.01%	16	0.01%	=0.96
	Acute upper respiratory infection unspecified (J06)	16	0.03%	52	0.03%	=0.96

For peer review only

For peer review only - http://bmjopen.bmj.com/site/about/guidelines.xhtml

	Pneumonia (J10-J18)	6	0.01%	19	0.01%	=0.96
	Acute bronchiolitis (J21)	70	0.12%	175	0.10%	=0.17
	Unspecified acute lower respiratory tract infection (J22)	3	0.01%	12	0.01%	*
**Diseases of the digestive system (K00-K93)**		**53**	0.09%	**92**	0.05%	=0.001
	Gastro-oesophageal reflux disease (K21)	26	0.04%	37	0.02%	=0.003
**Certain conditions originating in the perinatal period (P00-P96)**		**474**	0.81%	**1011**	0.56%	<0.001
	Cephalohaematoma due to birth trauma (P12.0)	14	0.02%	13	0.01%	=0.002
	Total birth trauma to scalp (P12)	30	0.05%	32	0.02%	<0.001
	Intrauterine hypoxia (P20)	4	0.01%	25	0.01%	*
	Other neonatal hypoglycaemia (P70.4)	14	0.02%	14	0.01%	=0.002
	Other transitory neonatal electrolyte and metabolic disturbances (P70.8)	5	0.01%	21	0.01%	=0.527
	Fever of newborn (P81.9)	5	0.01%	18	0.01%	0.753
	Feeding problems of newborn (P92)	40	0.07%	100	0.06%	=0.271
	**Neonatal jaundice (P58)**	**193**	0.33%	**90**	0.05%	<0.001
**Symptoms, signs and abnormal findings not elsewhere classified (R00-R99)**		**198**	0.34%	**340**	0.19%	<0.001
	Fever (R50)	19	0.03%	27	0.02%	=0.008

For peer review only - http://bmjopen.bmj.com/site/about/guidelines.xhtml

	Feeding difficulties and mismanagement (R63.3)	25	0.04%	37	0.02%	=0.003
	Excessive crying (R68.1)	83	0.14%	117	0.07%	<0.001
**Injury, poisoning and certain other consequences of external causes (S00-T98)**		**15**	0.03%	**94**	0.05%	=0.009
	Burns (T20-T31)	0	0.00%	30	0.02%	*
**Factors influencing health status and contact with health services (Z00-Z99)**		**192**	0.33%	**691**	0.39%	=0.051
	Observation and evaluation in newborn (Z03)	34	0.06%	102	0.06%	=0.907
	Prophylactic chemotherapy (antibiotics) (Z29.2)	5	0.01%	49	0.03%	=0.009
	Routine and ritual circumcision (Z41.2)	68	0.20%	119	0.13%	<0.001
	Attention to surgical dressings and sutures (Z48.0)	0	0.00%	28	0.02%	*
	Socio-economic circumstances (housing, distance, adoption, assumption of care) (Z76)	22	0.04%	156	0.09%	<0.001

***cell size too small to calculate chi-square ** as a % of male babies**

For peer review only

Table 7. Combined birth and readmission neonatal morbidity for selected codes

	Private	Public	P*
Total feeding difficulties	2314 (4.0%)	4306 (2.4%)	<0.0001
Total circumcision**	1620 (5.6%)	306 (0.3%)	<0.0001
Total socio-economic circumstances	54 (0.1%)	1176 (0.7%)	<0.0001
Total birth trauma	2922 (5.0%)	6492 (3.6%)	<0.0001
Total hypoxia	997 (1.7%)	2195 (1.2%)	<0.0001
Total jaundice	2818 (4.8%)	5359 (3.0%)	<0.0001
Total respiratory	717 (1.2%)	1366 (0.8%)	<0.0001
Total sleep/behavioural issues	118 (0.2%)	145 (0.1%)	<0.0001
Prophylactic antibiotics	98 (0.2%)	982 (0.6%)	<0.001

*chi-square ** as a % of male babies

For peer review only

Contributorship Statement: HD led the study and wrote the paper. ST helped in constructing the study design and writing the paper. MT helped in constructing the study design. AB helped in writing the paper. CB gave biostatistical support and helped in writing the paper. CT helped with study design, analysis and writing of the paper.

Funding: Nil

Competing Interests: None

Data Sharing Statement: No additional data available

For peer review only

Figure legends

Figure 1: Comparison of gestation at delivery between all low risk women stratified bu hospital type.

Figure 2: Birth trauma as a percentage of all births in Private and Public Hospitals.

References

1 Li Z, Zeki R, Hilder L, Sullivan EA. Australia's mothers and babies 2011. Perinatal statistics series no. 28. Cat. no. PER 59. In: Unit ANPEaS, editor. Canberra2013.

2 Robson SJ, Laws P, Sullivan EA. Adverse outcomes of labour in public and private hospitals in Australia: a population based descriptive study. The Medical Journal of Australia 2009;190(9):474-7.

3 Tracy S, Welsh A, Dahlen H, et al. Letter to the Editor re Robson SJ, Laws P, Sullivan EA. Adverse outcomes of labour in public and private hospitals in Australia: a population-based descriptive study. Med J Aust 2009; 190: 474-477. Medical Journal of Australia. 2009;191(10):579-80.

4 Clark E.A.S, Silver RM. Long-term maternal morbidity associated with repeat caesarean delivery. American Journal of Obstetrics and Gynecology. 2011;S2(December).

5 Hyde MJ, Mostyn A, Modi N, et al. The health implications of birth by caesarean section. Biological Reviews. 2012;87(1):229-43.

6 Tracy S, Tracy M, Sullivan E. Admission of Term Infants to Neonatal Intensive Care: A Population-Based Study. Birth 2007;34(4):301-7.

7 Tracy S, K , Tracy M. Costing the cascade: estimating the cost of increased obstetric intervention in childbirth using population data. British Journal of Obstetrics and Gynaecology. 2003;110:717-24.

8 Allen VM, O'Connell CM, Farrell SA, et al. Economic implications of method of delivery. Am J Obstet Gynecol 2005;193(1):192-7.

9 Dahlen HG, Tracy S, Tracy M, et al. Rates of obstetric intervention among low-risk women giving birth in private and public hospitals in NSW: a population-based descriptive study. BMJ Open. 2012;2:e001723 doi:10.1136/bmjopen-2012-001723.

10 Roberts C, L,, Tracy S, Peat B. Rates of obstetric intervention among private and public patients in Australia: population based descriptive study. British Medical Journal. 2000;312:137-41.

For peer review only

11 Buist R. Letter to the Editor: Private Obstetrics-again. BMJ Open. 2012;2(5):e001723 doi:10.1136/bmjopen-2012-.

12 CHeReL. Centre for Health Record Linkage (CHeReL). Quality Assurance Report 2012. http://wwwcherelorgau/media/24160/qa_report_2012pdf. 2012.

13 Lain SJ, Hadfield RM, Raynes-Greenow CH, et al. Quality of data in perinatal population health databases: a systematic review. Med Care. 2012;50(4):e7-e20.

14 Roberts CL, Tracy S, Peat B. Rates for obstetric intervention among private and public patients in Australia: population based descriptive study. British Medical Journal. 2000 2000;321(7254):137-41.

15 Einarsdóttir K, Stock S, Haggar F, et al. Neonatal complications in public and private patients: a retrospective cohort study. BMJ Open. 2013;Downloaded from bmjopen.bmj.com on November 11, 2013.

16 Evans N, Malcolm G, Gordon A. Letter to the Editor re Adverse outcomes of labour in public and private hospitals in Australia. MJA. 2009;191(10):579.

17 Chambers D. Letter to the Editor re Adverse outcomes of labour in public and private hospitals in Australia. MJA. 2009;191(10):578-9.

18 Wyllie J. Recent changes to UK newborn resuscitation guidelines. Arch Dis Child Fetal Neonatal Ed. 2012;97(1):F4-F7.

19 Kannapiran R, MKennea N. Resuscitation of the newborn. Obstetrics, Gynecology & Reproductive Medicine. 2012;22(4):92-7.

20 Doumouchtsis SK, Arulkumaran S. Head injuries after instrumental vaginal deliveries. Current Opion in Obstetrics and Gynaecology. 2006;18:129-34.

21 Towner D, Castro MA, Wilkens EE, et al. Effect of Mode of Delivery in Nulliparous Women on Neonatal Intracranial Injury. The New England Journal of Medicine 1999;341:1709-14.

22 Linder N, Linder I, Fridman E, et al. Birth trauma – risk factors and short-term neonatal outcome. The journal of Maternal-fetal & Neonatal Medicine. 2013;DOI: 10.3109/14767058.2013.789850.

23 Bertini G, Dani C, Tronchin M, et al. Is breastfeeding really favouring early neonatal jaundice? Pediatrics. 2006;107(3):e41.

24 Bhutani VK, Stark AR, Lazzeroni LC, et al. Predischarge Screening for Severe Neonatal Hyperbilirubinemia Identifies Infants Who Need Phototherapy. The Journal of Pediatrics. 2013;162(3):477-82.

25 Young PC, Korgenski K, Buchi KF. Early Readmission of Newborns in a Large Health Care System. Pediatrics. 2013;131(5):e1538-e44.

For peer review only

26 Brown A, Jordan S. Impact of birth complications on breastfeeding duration: an internet survey. Journal of Advanced Nursing. 2012;69(4):828-39.

27 Chapman D.J, Perez-Escamilla R. Identification of risk factors for delayed onset oflactation. Journal of the American Dietetic Association. 2003;99:450-4.

28 Dewey K.G., Nommsen-Rivers L.A., Heinig M.J, et al. Risk factors for suboptimal infant breastfeeding behavior, delayed onset of lactation and excess neonatal weight loss. Pediatrics 2003;112(3):607-19.

29 Scott J.A., Binns C.W, Oddy W.H. Predictors of delayed onset of lactation. Maternal and Child Nutrition. 2007;3(3):186-93.

30 Jordan S., Emery S., Watkins A., et al. Associations of drugs routinely given in labour with breastfeeding at 48 hours: analysis of the Cardiff Births Survey. British Journal of Gynecology. 2009;116(12):1622-9.

31 Leung G.M, Lam T.H, Ho L.M. Breast-feeding and its relation to smoking and mode of delivery. Obstetrics and Gynecology 2002;99(5):785-94.

32 Jordan S. Infant feeding and analgesia in labour: the evidence is accumulating. International Breastfeeding Journal Research. 2006;1(25):doi: 10.1186/746-4358-1-25.

33 Henderson J.J., Dickinson J.E., Evans S.F., et al. Impact of intrapartum epidural analgesia on breast- feeding duration. Australian and New Zealand Journal of Obstetrics and Gynaecology. 2003;43(5):372–7.

34 Beilin Y., Bodian C., Weiser J., et al. Effect of labor analgesia with and without fentanyl on infant breastfeeding: a prospective, randomized double-blind study. Anesthesiology. 2005;103(6):1211– 7.

35 Ohnsorg T, Schiff J. Preventing elective induction before 39 weeks. , 93(11), 44-46. Minnesota Medicine. 2010;93(11):44-6.

36 Oshiro BT, Henry E, Wilson J, et al. Decreasing elective deliveries before 39 weeks of gestation in an integrated health care system. Obstetrics & Gynecology. 2009;113:804-11.

37 Reddy UM, Ko CW, Willinger M. “Early term” births (37-38 weeks) are associated with increased mortality. American Journal of Obstetrics & Gynecology. 2006;195(S202).

38 McLaughlin FJ, O’Connor S, Deni R. Infant state and behavior during the first postpartum hour. The Psychological Record 1981;31:455–8.

For peer review only

39 Widstrom A-M, Ransjo-Arvidson AB, Christensson K, et al. Gastric suction in healthy newborn infants. Effects on circulation and developing feeding behaviour. Acta Paediatr. 1987;76:566–72.

40 Righard L. How do newborns find their mother's breast? . Birth. 1995;22:174–5.

41 Fisher J, Astbury J, Smith A. Adverse psychological impact of opera- tive obstetric interventions: a prospective study. Aust NZ J Psychiatry 1997;31:728–38.

42 Adams-Chapman I. Insults to the developing brain and impact on neurode- velopmental outcome. Journal of Communication Disorders. Journal of Communication Disorders. 2009;42:256-62

43. Gülmezoglu AM, Crowther CA, Middleton P, et al. Induction of labour for improving birth outcomes for women at or beyond term. *Cochrane Database of Systematic Reviews* 2012;Issue 6. Art.No.:CD004945. DOI: 10.1002/14651858.CD004945.pub3.

44. The Royal Australian College of Physicians. Circumcision of infant males. In: Physicians TRACo, editor. Sydney: The Royal Australasian College of Physicians; 2010.

45. Bentley JP, Ford JB, Taylor LK, et al. Investigating linkage rates among probabilistic linked births and hospital records. BMC Medical Research Methodology. 12:149, 2012.

46. Taylor L, Travis S, Pym M, et al. How useful are hospital morbidity data for monitoring conditions occurring in the perinatal period? Australian & New Zealand Journal of Obstetrics & Gynaecology. 2005;45:36 –41.

47 Roberts C, Bell J, Ford J, et al. Monitoring the quality of maternity care: how well are labour and delivery events reported in population health data? . Paediatirc and Perinatal Epidemiology. 2008;23:144-52.

48 Thornton C, Makris A, Ogle R, et al. Generic obstetric database systems are unreliable for reporting the Hypertensive Disorders of Pregnancy. Australian and New Zealand Journal Obstetrics and Gynaecology. 2004;44(505-509).

49 Finkelstein A, Fiebelkorn IC, Wang G. National medical Spending attributable to overweight and obesity: How much and who's paying. Health Affairs, 2003 Project HOPE–The People-to-People Health Foundation, Inc., 219-226. 2003.

For peer review only

For peer review only

Rates of obstetric intervention and associated perinatal mortality and morbidity amongst low risk women giving birth in private and public hospitals in NSW (2000-2008): A linked data population based cohort study.

Hannah G. Dahlen RN, RM, BN (Hons), MCommN, PhD, FACM

Associate Professor of Midwifery

School of Nursing and Midwifery

Family and Community Health Group

Locked Bag 1797

Penrith South DC

NSW

Australia

Ph: +61 2 96859118

h.dahlen@uws.edu.au

Sally Tracy

Professor Midwifery

Professor of Midwifery

University of Sydney,

Royal Hospital for Women, Sydney

Tel: 0420277106

For peer review only

For peer review only - http://bmjopen.bmj.com/site/about/guidelines.xhtml

Mark Tracy

Senior Newborn Intensive Care Specialist and HOD

CNBC Westmead Hospital

Senior Clinical Lecturer Paediatrics and Child Health

University of Sydney

Tel (02) 9845 6568

Andrew Bisits Mb bs(unsw)franzcog ,mmed stat ,dip clin epi

Acting Medical Co Director of Maternity, Lead Clinician Birthing Suite

Royal Hospital for Women

Conjoint Associate Professor School of Women and Children's Health University of NSW

Randwick NSW 2033

0419418492

Chris Brown, BSc, MBiostat

Biostatistician

NHMRC Clinical Trials Centre

Locked Bag 77

Camperdown NSW 1450

For peer review only - http://bmjopen.bmj.com/site/about/guidelines.xhtml

Charlene Thornton BN, Grad.Dip.Mid, M Sc Med (Research), M Sc Med (Clin Epi), PhD

Post Doctoral Research Fellow

School of Nursing and Midwifery

Family and Community Health Group

Locked Bag 1797

Penrith South DC

NSW

Australia

Keywords: low risk primiparous, low risk multiparous , induction, vaginal birth, obstetric care, caesarean, birth trauma

Word count 4596

Ethical approval was obtained from the NSW Population and Health Services Research Ethics Committee, Protocol No.2010/12/291.

Ni funds allocated

Nil extra data

For peer review only - http://bmjopen.bmj.com/site/about/guidelines.xhtml

Rates of obstetric intervention and associated perinatal mortality and morbidity amongst low risk women giving birth in private and public hospitals in NSW (2000-2008): A linked data population based cohort study.

Abstract

Objectives: To examine the rates of obstetric intervention and associated perinatal mortality and morbidity in the first 28 days amongst low risk women giving birth in private and public hospitals in NSW (2000-2008).

Design: Linked data population based retrospective cohort study involving five data sets.

Setting: New South Wales, Australia.

Participants: 691,738 women giving birth to a singleton baby during the period 2000 to 2008.

Main outcome measures: Rates of neonatal resuscitation, perinatal mortality, neonatal admission following birth and readmission to hospital in the first 28 days of life in public and private obstetric units.

Results: Rates of obstetric intervention amongst low risk women were higher in private hospitals, with primiparous women 20% less likely to have a normal vaginal birth compared to the public sector. Neonates born in private hospitals were more likely to be less than 40 weeks; more likely to have some form of resuscitation; less likely to have an Apgar <7 at five minutes. Neonates born in private hospitals to low risk mothers were more likely to have a morbidity attached to the birth admission and to be readmitted to hospital in the first 28 days for birth trauma (5% vs 3.6%); hypoxia (1.7% vs 1.2%) ; jaundice (4.8% vs 3%); feeding difficulties (4% vs 2.4%) ; sleep /behavioural issues (0.2% vs 0.1%); respiratory conditions (1.2% vs 0.8%) and circumcision (5.6 vs 0.3%) but they were less likely to be admitted for prophylactic antibiotics (0.2% vs 0.6%) and for socioeconomic circumstances (0.1% vs 0.7%). Rates of perinatal mortality were not statistically different between the two groups.

For peer review only - http://bmjopen.bmj.com/site/about/guidelines.xhtml

For peer review only

Conclusion:

For low risk women, care in a private hospital, which includes higher rates of intervention, appears to be associated with higher rates of morbidity seen in the neonate, and no evidence of a reduction in perinatal mortality

For peer review only

For peer review only - http://bmjopen.bmj.com/site/about/guidelines.xhtml

Article focus:

- To examine the rates of obstetric intervention and associated perinatal mortality and morbidity in the first 28 days amongst low risk women giving birth in private and public hospitals in NSW (2000-2008) using linked data.
- Rates of neonatal resuscitation, perinatal mortality, neonatal admission following birth and readmission to hospital in the first 28 days of life amongst those infants born in public and private obstetric units.

Key messages

- Babies born to low risk mothers in private hospitals are more likely to require some resuscitation compared to those born in public hospitals.
- For babies born in private hospitals to low risk mothers there are higher rates of morbidity such as birth trauma, hypoxia, jaundice, feeding difficulties, sleep/behavioural issues, respiratory conditions and circumcision. For babies born in public hospitals there are higher rates of antibiotic use and admission for socio-economic reasons (eg housing, distance, adoption and assumption of care)
- Rates of perinatal mortality were comparable between babies born to low risk women giving birth in private and public hospitals

Strengths and limitations of this study

- The strength of this study lies in the large sample size of linked birth data admissions associated with these births.
- The use of data from five population based datasets which have been linked to enhance validity and ascertainment
- Limitations are the restricted number of variables that are included and the scarcity of specific information on potential confounders.
- Body mass index and key sociodemographic risk factors could not be controlled for and this would have added risk to women giving birth in public hospitals.

For peer review only - http://bmjopen.bmj.com/site/about/guidelines.xhtml

Introduction

In Australia, the national statistics reveal that 29% (n =83,573) of women who gave birth in hospital gave birth in private hospitals directly under private obstetric care [1}. The remaining 71% (n=204,399) of women gave birth in public hospitals in Australia. Women who are privately insured have been reported to have better maternal and perinatal outcomes compared to women who give birth in public hospitals as public patients [2]; but it has been argued that these women tend to be less socioeconomically disadvantaged and healthier [3] and therefore might be expected to have better outcomes. Arguments about the impact of private status on health outcomes are in reality complex.

What is not disputed are the much higher rates of obstetric intervention that occur in private hospitals in Australia. At a national level, the intervention rates in childbirth, such as caesarean section, are significantly higher in the private sector (43.1% vs 28.4%) and the rates of normal vaginal birth significantly lower (42.7% vs 61%) [1]. Despite the rising intervention rates over the past decade, the perinatal mortality rate overall has not shown a corresponding decline [1]. There is also growing concern that the short and long term morbidity associated with major obstetric interventions, such as caesarean, may not be insignificant for the mother [4] and the baby [5, 6]. The cost to the tax payer of the rising intervention in childbirth is also significant [7, 8].

A recent study in New South Wales, Australia [9] found among 293,840 low-risk women, rates of obstetric intervention were highest in private hospitals and lowest in public hospitals. Low-risk primiparous women giving birth in a private hospital compared to a public hospital had higher rates of induction (31% vs 23%); instrumental birth (29% vs 18%); caesarean section (27% vs 18%), epidural (53% vs 32%), episiotomy (28% vs 12%) and lower normal vaginal birth rates (44% vs 64%). Low-risk multiparous women had higher rates of instrumental birth (7% vs 3%), caesarean section (27% vs 16%), epidural (35% vs 12%), episiotomy (8% vs 2%) and lower normal vaginal birth rates (66% vs 81%). Following a comparison with data from the decade previously [10], these interventions were found to have increased by 5% for women in public hospitals

For peer review only - http://bmjopen.bmj.com/site/about/guidelines.xhtml

and by over 10% for women in private hospitals [9]. Among low-risk primiparous women giving birth in private hospitals, 15 per 100 women had a vaginal birth with no obstetric intervention compared to 35 per 100 women giving birth in a public hospital [9]. Concern was expressed that perinatal mortality and morbidity was not reported in that paper [11]. In this study we aim to address this through examining the rates of obstetric intervention and associated perinatal mortality and morbidity attached to the birth admission and readmission to hospital in the first 28 days of life for low risk women giving birth in private and public hospitals in NSW (2000-2008).

Methods

Data Sources

The New South Wales Centre for Health Record Linkage conducted linkage of several datasets via the Health Record Linkage (CHeReL). Pregnancy and birth data for the time period July 1st 2000 till June 2nd 2008 of all singleton births were provided by New South Wales (NSW), Ministry of Health as recorded in the NSW Midwives Data Collection (MDC), now the Perinatal Data Collection (PDC). This population based surveillance system contains maternal and infant data on all births of greater than 400 grams birth weight and/or 20 completed weeks gestation. Hospitals are coded either as private or public in the data set. However, the data identifying women who received care in public hospitals under private accommodation status is no longer collected as it had been in the years 1996-97 and for this reason patients who are under private obstetric care in public hospitals are not able to be differentiated from their public counterparts, so for this study we analysed the data by hospital (private/public). A previous study published in 2000 [10] showed that there was a moderating factor on intervention rates when women with private insurance status gave birth in a public hospital, leading to lower intervention rates than when they gave birth in private hospitals.

The NSW PDC contains statistics on all births in New South Wales - which amounts to one third of all births which occur in Australia annually. Data is provided for maternal age, maternal hypertension, maternal diabetes, parity, private/public patient status, fetal presentation, onset

For peer review only - http://bmjopen.bmj.com/site/about/guidelines.xhtml

of labour, gestation at birth, delivery type, Apgar scores and admission to neonatal intensive care and resuscitation details for the neonate. This dataset (NSW PDC) was linked to the Admitted Patient Data Collection (APDC) for the time period July 1st 2000 – June 30th 2008. The APDC records all admitted patient services provided by NSW Public Hospitals, Public Psychiatric Hospitals, Public Multi-Purpose Services, Private Hospitals, and Private Day Procedures Centres. The APDC provided additional information, such as data on maternal medical conditions, which was used to exclude further maternal cases and was used to calculate admission and readmission details for neonates. Further linkage occurred to the NSW Registry of Births, Deaths and Marriages (RBDM) and the Australian Bureau of Statistics Death Data, which provided mortality data. The NSW Register of congenital conditions provided cases of congenital conditions, as did the coding in the APDC. Any neonate (and mother pair) with a recorded congenital condition (ICD-10-AM codes Q0.0-Q99.9) on either dataset was removed from the dataset due to their high-risk status. Probabilistic data linkage techniques were utilised for data linkage and de-identified datasets were provided for analysis. Probabilistic record linkage software assigns a 'linkage weight' to pairs of records. For example, records that match perfectly or nearly perfectly on first name, surname, date of birth and address have a high linkage weight, and records that match only on date of birth have a low linkage weight. If the linkage weight is high it is likely that the records truly match, and if the linkage weight is low it is likely that the records are not truly a match. This technique has been shown to have a false positive rate of 0.3% of records [12].

Gestation is recorded at birth and is also recorded in the database according to the woman's menstrual history, usually combined with a routine scan at 12-13 weeks.

Admission to neonatal intensive care refers to admission to special care nursery (SCN) or neonatal intensive care unit (NICU).

Any resuscitation includes suction of the mouth or nostrils at birth; oxygen administered by mask; intermittent positive pressure respiration (IPPR) by bag and mask or by intubation; external cardiac massage and ventilation.

Ethical approval was obtained from the NSW Population and Health Services Research Ethics Committee, Protocol No.2010/12/291.

For peer review only - http://bmjopen.bmj.com/site/about/guidelines.xhtml

Subjects

We classified the low risk primipara as a first time mother aged 20-34 years, who had no pre-existing or pregnancy related hypertension or diabetes, was a non-smoker, and gave birth at 37-41 completed weeks gestation to a singleton baby in a cephalic presentation within the 10th and 90th centiles for gestation and birth weight. The low risk multipara was a woman having her second or subsequent baby aged 20-34 years, who had no pre-existing or pregnancy related hypertension or diabetes, was a non-smoker, gave birth at 37-41 completed weeks gestation to a singleton baby in a cephalic presentation within the 10th and 90th centiles for gestation and birth weight. We excluded women with a previous caesarean section or who were induced for a medical indication, or who underwent a caesarean section for a pre-existing medical indication or gave birth without a trained birth attendant (born before arrival). If a caesarean section was undertaken during labour however for non-reassuring heart rate, dystocia etc these women were included in the study. These characteristics were defined firstly from the PDC with additional medical conditions identified in the APDC being used to exclude cases.

Outcomes

Any neonatal admission including the ICD-10-AM codes Z37.0 (single live birth), Z37.1 (single stillbirth) or Z38.0 (singleton born in hospital) was deemed the birth admission and any ICD-10-AM codes referring to conditions which arise in the perinatal period (P00-P96) and those referring to factors influencing health status and contact with health services (Z00-Z99) which were included in this admission were deemed morbidities associated with the birth admission. Any other admission following this discharge from the initial birth admission to home or another hospital was deemed a readmission and included transfers to a hospital other than that where the birth occurred. When examining readmission data, all ICD-10-AM codes recorded were reviewed and those where ≥10 events occurred in either private or public hospitals were marked for analysis. Events were grouped in body systems where appropriate or under headings such as infection for ease of analysis and interpretation.

For peer review only - http://bmjopen.bmj.com/site/about/guidelines.xhtml

Morbidity was recorded with the birth admission and rates of events were calculated using the number of babies who had any morbidity recorded with their birth.

Stillbirth and neonatal deaths were calculated from multiple sources but were limited to those that occurred within 28 days of birth and they were only counted once. Death may have been detected on any one of the following four datasets. The PDC 'Discharge status' variable or admissions in the APDC where the case mode separation was coded as 'Died' or the NSW RBDM or ABS Death Data where a death had been recorded. The maternal admission data for any admission that occurred during the pregnancy, as well as the birth admission for all cases of stillbirth or neonatal death were examined to determine any maternal medical or pregnancy related condition. This methodology of utilising multiple data sources to identify cases has been shown by Lain et al (2012) to be the most reliable way to increase ascertainment of cases [13].

Obstetric intervention was defined to include induction, epidural use, episiotomy, instrumental delivery (requiring the use of forceps or vacuum) and delivery via caesarean section.

Data analysis

The cohort was divided into primiparous and multiparous women for the primary analysis of birth outcomes. When examining neonatal status at birth mortality odds ratios were calculated using logistic regression with and without adjustment for age and gestation. For neonatal morbidity at birth and readmission, chi-square statistics were calculated for observed events. The total number of babies born in a public or a private hospital were used as the denominator when calculating the percentage of babies born with a morbidity code attached to their birth record or the number of babies readmitted with a designated morbidity code. This methodology provides for comparison between place of birth taking into consideration the fact that up to 55 morbidity codes can be attached to any one birth or readmission record. Taking into account the size of the cohort and the number of analyses undertaken, results were considered significant at the level $p<0.01$. Analysis was undertaken with IBM SPSS v.20®

For peer review only - http://bmjopen.bmj.com/site/about/guidelines.xhtml

Results

Maternal characteristics, interventions and outcomes

The PDC dataset for the time period July 1st 2000 to 2nd June 2008 contained the antenatal, birth and postnatal details on 691 738 births. The APDC for the time period July 1st 2000 to 30th June 2008 contained >1.1 million admissions for the neonates/children of these women.

From the total population of primiparous women (288,309 women), 29,597 low risk primiparous women gave birth in private hospitals in NSW and 79,792 low risk primiparous women gave birth in public hospitals. The rates of obstetric intervention were much higher amongst those who gave birth in private hospitals compared to those who gave birth in public hospitals when all interventions for pre-specified medical reasons were removed. Low risk primiparous women giving birth in private hospitals compared to low risk primiparous women giving birth in public hospitals had higher rates of induction for non-medical reasons (19% vs 7%), instrumental birth (30% vs 20%), caesarean section (25% vs 16%), epidural (71% vs 35%) and episiotomy (42% vs 23%). Severe perineal trauma (defined as third and fourth degree perineal trauma) was lower in a private hospital in first time mothers (4.7% vs 5.4%) (Table 1).

Amongst the total population of multiparous women (403,429 women), 28,703 low risk multiparous women gave birth in private hospitals and 99,212 low risk multiparous women gave birth in public hospitals The rate of obstetric intervention was significantly higher amongst those who gave birth in private hospitals in NSW compared to those who gave birth in public hospitals when all interventions for specific medical reasons were removed. Low risk multiparous women who gave birth in private hospitals compared to low risk primiparous women giving birth in public hospitals had higher rates of induction for non- medical reasons (32% vs 10%), instrumental birth (8% vs 3%), epidural (34% vs 10%) and

For peer review only - http://bmjopen.bmj.com/site/about/guidelines.xhtml

episiotomy (16% vs 5.%) and similar rates of severe perineal trauma (0.9%). The caesarean section rate still remained higher in the private cohort (5.9% vs 4.5%) though this was mostly associated with elective caesarean section (Table 2).

Perinatal characteristics, interventions and outcomes

There was no difference in birth weight between babies born in a private and public hospital. Babies born in a private hospital were more likely to be born at 37, 38, 39 and 40 weeks and less likely to be born at 41 weeks gestation (Figure 1.).

Babies of primiparous women who gave birth in a private hospital were less likely to have an Apgar of <7 at five minutes (AOR 1.34 95% CI 1.18-1.53; p<0.001) as were babies of multiparous women who gave birth in private hospitals (AOR 1.37 95% CI 1.14-1.64). babies born in private hopsitals were less likely to have no resuscitation (AOR 0.36 95% CI 0.35-0.37; p<0.001). Babies born to low risk primiparous women in a private hospital were no more likely to be admitted to special care and/or neonatal intensive care (AOR 1.03 95% CI 0.98-1.08; p 0.210) and were less likely to have their baby transferred to another hospital (AOR 7.55 95% CI 6.52-8.74; p<0.001). There was no difference in the perinatal mortality rate for babies of primiparous women born in private or public hospitals (AOR 1.49 95% CI 0.93-2.41; p 0.10) (Table 3). Similar outcomes were seen for babies born to multiparous women in private and public hospitals however there was no difference in the incidence of Apgars of <7 at five minutes (Table 4).

Reason for birth admission of neonate

We examined neonatal morbidity as coded on the neonatal birth admission record and found fewer babies overall had a morbidity recorded (ICD-10-AM code other than the birth code) in the private sector compared to the public sector (40.0% vs 53.9%) due to the added clinical and social maternal complexity in the public sector. There were however some significant differences noted under the main ICD-10-AM Grouping *Certain conditions originating in the perinatal period (P00-P96)*. Babies born in a private hospital were more likely to have been affected by a

For peer review only - http://bmjopen.bmj.com/site/about/guidelines.xhtml

forceps or vacuum delivery and were more likely to have trauma to the scalp (3.22% vs 2.22%), intrauterine hypoxia (1.70% vs 1.21%), jaundice (4.68% vs 2.89%), minor cardiac murmurs (0.24% vs 0.17%), conjunctivitis (2.98% vs 1.27%), respiratory conditions (0.96% vs 0.57%), temperature regulation issues (2.08% vs 1.27%) feeding difficulties (3.83% vs 2.32%), carbohydrate metabolism issues (0.86% vs 0.53%), vomiting (0.55% vs 0.39%) and to be circumcised during the birth admission if a male (5.31% vs 0.21%)). Babies born in a public hospital were more likely to be admitted for observation and evaluation (6.35% vs 3.75%) have prophylactic antibiotics (0.52% vs 0.16%) and be admitted for socioeconomic circumstances (eg. housing, distance, adoption, assumption of care) (0.57% vs 0.05%) (Table 5)

Reason for birth re-admission of neonate up to 28 days of age

We examined the reasons for transfer or readmission of babies up until 28 days of age and found, that though the numbers are small, more babies born in private hospitals were readmitted compared to babies born in a public hospital (0.95% vs 0.65%) (Table 6). Babies born in private hospitals were more likely to be readmitted for infectious diseases (0.21% vs 0.12%) endocrine, nutritional and metabolic disorders (0.05% vs 0.02%), sleep disorders (0.03% vs 0.01%), hypoglycaemia (0.02% vs 0.01%), birth trauma such as cephalohaematoma (0.02% vs 0.01%); trauma involving the scalp (0.05% vs 0.02%), excessive crying (0.14% vs 0.07%), behavioural disorders (0.06% vs 0.02%) and for circumcision if a male (0.20 vs 0.13)). Babies born in public hospitals were more likely to be readmitted with respiratory disorders (0.27% vs 0.20%), injury and poisoning (eg burns) (0.05% vs 0.03%), antibiotic therapy (0.03% vs 0.01%) and socioeconomic circumstances (housing, distance, adoption, assumption of care (0.09% vs 0.04%).

Combined birth and readmission neonatal morbidity for selected codes

When we combined major birth and readmission morbidities for key selected codes we found that in the first 28 days following birth, babies born in private hospitals were significantly more likely to be admitted for feeding difficulties (4% vs 2.4%), circumcision if a male (5.6 vs 0.3)), birth trauma (mostly scalp trauma) (5% vs 3.6%), jaundice (4.8% vs 3.0%), hypoxia (1.7% vs 1.2%), respiratory disorders (1.2% vs 0.8%) and

For peer review only - http://bmjopen.bmj.com/site/about/guidelines.xhtml

sleep/behavioural issues (0.2% vs 0.1%). Babies born in public hospitals were more likely to be admitted for socioeconomic circumstances such as housing, distance, adoption or assumption of care (0.7% vs 0.1%) and prophylactic antibiotics (0.6% vs 0.2%) (Table 7 & Figure 2).

Discussion

Intervention rates

Despite being an extremely low risk cohort, less than half the primiparous women in this study giving birth in a private hospital had a normal vaginal birth (45% vs 65%); this was 20% lower than in the public cohort. One in five primiparous women giving birth in a private hospital were induced and nearly one in two had an episiotomy. For low risk multiparous women giving birth in a private hospital nearly one in three were induced. The trend for higher intervention rates has been reported for low risk women giving birth in the private sector in Australia previously and continues to show an increase [9, 14, 15]. In a recent publication we showed that the rate of caesarean section had increased in both the private and public sector in the past decade in low risk women [9]. It has been argued in a previous publication that these high intervention rates in the private sector led to better perinatal outcomes than in the public sector [2]. This publication received significant criticism in letters to the editor [3, 16, 17] for several methodological flaws, including most significantly the failure to adjust for low birth weight, inadequate ascertainment of congenital abnormalities and failure to look at perinatal morbidity. In this study we included only low risk women, adjusting for maternal age and gestational age differences. We also removed all babies with congenital abnormalities from this data set. We found that the perinatal mortality rate was not statistically different when the populations were matched in this data set for maternal risk.

Neonatal resuscitation and admission to SCN/NICU

We found that babies born in a private hospital were much more likely to experience some form of resuscitation, in particular twice the rate of suctioning at birth. Routine suctioning for infants born with clear and/or meconium stained amniotic fluid is not recommended [18] as it can cause a bradycardia [19] and there is no evidence of benefit. We are unsure why such a high rate of newborn suctioning continues in the

For peer review only - http://bmjopen.bmj.com/site/about/guidelines.xhtml

private sector. Rates of Apgar scores of ≤7 at five minutes were slightly higher amongst low risk women who gave birth in public hospital, and this has been demonstrated in another recent Australian publication [15], overall the babies were no more likely to be admitted to SCN/NICU compared to babies born in private hospitals.

Neonatal admission and readmission

We found some interesting differences in morbidity however when examining morbidity attached to the birth admission and readmission to hospital in the first 28 days for codes that may be associated with the higher rates of obstetric intervention in the private sector and a different sociodemographic profile in the public sector. While increasingly preterm babies >35 weeks/ and or >2.2k and some cases of jaundice may be managed at the bedside in some hospitals, this is less likely to occur in a private hospital. It is more likely to occur in large maternity units.

Birth trauma, in particular injuries to the scalp, were significantly more common in the private sector and these are generally associated with instrumental birth, including vacuum extraction [20-22]. With more women (nearly one in three primiparous women) experiencing an instrumental birth in the private cohort and one in five women in the public sector this is not surprising. Birth trauma is associated with a longer hospital stay and increased risk of admission to SCN/NICU as well as higher rates of neonatal morbidity including neurological morbidity (hypotonia, jitteriness, convulsions and hypoxic ischaemic encephalopathy) and jaundice [22].

Jaundice was observed to be higher in the private sector, which may be related to several factors, such as the elective delivery of babies at an earlier gestation, the increased scalp trauma due to the high instrumental birth rate, as discussed above, and potential breastfeeding difficulties due to higher use of epidural analgesia. Jaundice has been associated with birth trauma, in particular delivery by vacuum extraction, and problems with feeding, especially supplementary feeding [22, 23]. Earlier gestational age <39 weeks has also be found to be associated with jaundice, with this decreasing with each week of additional gestation [24]. The gestational age of babies born in private hospitals in this

For peer review only - http://bmjopen.bmj.com/site/about/guidelines.xhtml

study was significantly lower than in the public sector possibly due to the high rates of non-medical induction of labour and non-medically indicated caesarean section before the onset of labour.

While there have been studies associating difficulties with breastfeeding and higher rates of jaundice, the recent publication from the Universal Screening for Hyperbilirubinemia Study Group found difficulties with breastfeeding was a minimal risk factor [24].

Nearly twice as many babies who were born in a private hospital in this study were admitted or re-admitted with feeding problems compared to babies born in a public hospital. Feeding difficulties are associated with operative birth interventions and being early term [25, 26]. Breastfeeding outcomes are positively associated with uncomplicated unassisted vaginal birth where the mother and infant remain together and breastfeeding is started within an hour of the birth and following skin-to-skin contact. Interventions during labour and birth can impact on the initiation and duration of breastfeeding. Caesarean section [27-30], instrumental birth [31], epidural anaesthesia and opioid analgesia [32-34] use have all been associated with breastfeeding difficulties. All these birth interventions were higher in the private cohort in this study. In addition early term birth, which is mainly due to induction of labour and elective caesarean section (35, 36) is associated with increased breastfeeding difficulties along with other serious morbidities [37]. Unmedicated newborns are more highly aroused immediately following the birth [38] and able to breastfeed without assistance if given skin to skin contact and freedom from intrusive procedures [39, 40]. Following caesarean section there can be a significantly longer period of time until a mother touches and holds her newborn compared to an unassisted vaginal birth [41].

In a previous paper [6] using national Australian population data we found that among low-risk women who had an unassisted vaginal birth with spontaneous onset of labour and no labour augmentation, the odds of admission to neonatal intensive care or special care nursery were significantly increased when the baby was 37 weeks gestation at the time of birth compared to later gestations. Some claim that during the final weeks of gestation the fetal brain goes through a marked increase in mass and nerve growth (corticoneurogenesis) which may be best left

For peer review only - http://bmjopen.bmj.com/site/about/guidelines.xhtml

For peer review only

undisturbed by allowing the normal gestational length to occur [42]. In this study low risk women giving birth in private hospitals in NSW were much more likely to give birth at earlier gestations than their public hospital counterparts for every week up to and including 40 weeks, but they were significantly less likely to deliver at 41 weeks. This may also help to explain why more babies born in a private hospital were readmitted with, respiratory, feeding, jaundice and sleep and behavioural problems. However there is also evidence that there are increased adverse perinatal outcomes for babies born following 41 completed weeks, but we did not examine this population [43].

Circumcision

Babies born in a private hospital were significantly more likely to be circumcised in the first 28 days of life. This may be due to different information being given in private hospitals about the procedure or easy access to providers who perform the procedure. Circumcision rates are estimated to be between 10-20% in Australia [44] and are decreasing. A recent position statement of the Royal Australian College of Physicians states “that the frequency of diseases modifiable by circumcision, the level of protection offered by circumcision and the complication rates of circumcision do not warrant routine infant circumcision in Australia and New Zealand” [44].

Socioeconomic circumstances

The difference in the socioecononomic status of the women giving birth in public compared to private hospitals appears to be demonstrated by the significantly higher rates of public hospital babies with a morbidity attached to the birth admission or readmission in the first 28 days for socioeconomic circumstances, including housing, distance, adoption and assumption of care. This again confirms what is already known that the two populations are very different sociodemographically with greater disadvantage in the public sector.

For peer review only - http://bmjopen.bmj.com/site/about/guidelines.xhtml

Limitations

Our study is limited to providing a snapshot of perinatal outcomes in the most populous state in Australia in a defined time period for women who have no indicated risk at birth. However, this study provides useful data following on from our previous paper looking at obstetric intervention in private and public hospitals in NSW providing the reader with a detailed picture of perinatal mortality and morbidity. The advantages of using population-based datasets such as the PDC and the linkage to four other population-based databases include the size of the sample and the high level of accuracy of a validated dataset. The limitations are the restricted number of variables that are included and the scarcity of specific information on potential influencing variables. A small number of cases with a low linkage (false/positive) rate (0.3%) were not included and so there is the possibility of missing adverse outcomes. A previous study showed that where stillbirths are excluded due to low linkage these are at lower gestational ages and not term infants as were the focus in this study [45]. Previous validation studies have reported high levels of data accuracy for the majority of diagnoses and procedures conducted during labour and delivery in the state-wide data base [46, 47], although the recording of medical conditions and smoking are overall generally underreported [46, 48]. Having a linked data set provides a much richer picture than we have had previously of the morbidity and mortality associated with birth interventions. While we could not control for obesity due to lack of data, women who have private health insurance have lower rates of obesity and higher socioeconomic status, hence these health disadvantages are most likely over represented in the public women [49]. There are also several other socio-demographic factors we could not control for, such as education and income, that increase risk for the women giving birth in public hospitals. This study can only provide an overview of possible associations between obstetric interventions and neonatal outcomes and does not imply causality, which could be better obtained from prospective cohort studies.

For peer review only - http://bmjopen.bmj.com/site/about/guidelines.xhtml

Conclusion

The continual rise in obstetric intervention for low risk women in Australia, especially in private hospitals, may be contributing to increased morbidity for healthy women and babies and higher cost of health care. The fact that these procedures which were initially life-saving are now so commonplace and do not appear to be associated with improved rates of perinatal mortality or morbidity demands close review. Early term delivery and instrumental births may be associated with increased morbidity in neonates and this requires urgent attention. Previous claims that high intervention rates in private hospitals lead to better perinatal outcomes than those seen in public hospitals need to be questioned.

For peer review only

Table 1. Maternal characteristics, interventions and outcomes for low risk primiparous women in NSW (2000-2008)

Low risk Primiparous women	Private Hospital (n=29597)	Public Hospital (n=79792)
**Maternal age (years)**		
20-24	6.2%	28.9%
25-29	39.9%	40.9%
30-34	53.9%	30.2%
Weeks gestation at delivery		
37	4.5%	4.3%
38	15.4%	11.8%
39	27.8%	25.0%
40	43.2%	39.7%
41	9.1%	19.2%
**Type of labour**		
Spontaneous	71.9%	89.9%
Induced	19.2%	7.1%
No labour	8.9%	3.0%
**Delivery**		
Normal vaginal	44.9%	64.8%

For peer review only - http://bmjopen.bmj.com/site/about/guidelines.xhtml

Forceps	11.5%	6.7%
Vacuum	18.9%	12.9%
Total caesarean section	24.7%	15.6%
Caesarean section (after labour)	15.9%	12.6%
Caesarean section before the onset of labour	8.8%	3.0%
**Epidural**	70.8%	35.4%
**Episiotomy**	42.4%	23.3%
**Severe perineal trauma**	4.7%	5.4%

For peer review only

For peer review only - http://bmjopen.bmj.com/site/about/guidelines.xhtml

Table 2. Maternal characteristics, interventions and outcomes for low risk multiparous women in NSW (2000-2008)

Low risk Multiparous women	Private Hospital (n=28703)	Public Hospital (n=99212)
**Maternal age (years)**		
20-24	2.1%	16.8%
25-29	25.8%	38.8%
30-34	72.1%	44.4%
Weeks gestation at delivery		
37	4.1%	4.0%
38	18.7%	13.0%
39	31.6%	26.9%
40	40.2%	40.8%
41	5.4%	15.3%
**Type of labour**		
Spontaneous	64.0%	87.4%
Induced	32.1%	10.1%
No labour	3.9%	2.5%
**Delivery**		
Normal vaginal	86.1%	92.7%

For peer review only - http://bmjopen.bmj.com/site/about/guidelines.xhtml

For peer review only

Forceps	1.9%	0.7%
Vacuum	6.1%	2.1%
Total caesarean section	5.9%	4.5%
Caesarean section after labour	2.0%	2.0%
Caesarean section before the onset of labour	3.9%	2.5%
**Epidural**	34.4%	9.5%
**Episiotomy**	16.2%	5.1%
**Severe perineal trauma**	0.9%	0.9%

For peer review only

For peer review only - http://bmjopen.bmj.com/site/about/guidelines.xhtml

Table 3. Perinatal outcomes adjusted for maternal age and gestation at birth for low risk primiparous women

	Private	Public			
	(n=29597)	(n=79791)	OR**	AOR**	p
Apgar<7 at 5 minutes	296 (1.0%)	1037 (1.3%)	1.36 (1.12-1.54)	1.34(1.18-1.53)	<0.001
Any resuscitation*	18498 (62.5%)	30560 (38.3%)	0.372 (0.36-0.38)	0.364 (0.36-0.37)	<0.001
Admitted to SCN and/or NICU	3078 (10.4%)	8139 (10.2%)	1.00 (0.96-1.05)	1.03 (0.98-1.08)	0.210
Transferred	178 (0.6%)	3351 (4.2%)	7.30 (6.29-8.40)	7.55 (6.52-8.74)	<0.001
Total Perinatal Mortality	22 (0.74/1000)	85 (1.06/1000)	1.40 (0.93-2.01)	1.49 (0.93-2.41)	0.100

*Any resuscitation includes: Suction, oxygen, IPPR by bag and mask, Intubation and IPPR, external cardiac massage and ventilation and other

** Private hospital is the reference category

For peer review only - http://bmjopen.bmj.com/site/about/guidelines.xhtml

Table 4. Perinatal outcomes adjusted for maternal age and gestation at birth for low risk multiparous women

	Private	Public			
	(n=28703)	(n=99212)	OR**	AOR**	p
Apgar<7 at 5 minutes	149 (0.5%)	676 (0.7%)	1.32 (1.10-1.57)	1.37 (1.14-1.64)	0.001
Any resuscitation*	14820 (51.6%)	29867 (30.1%)	0.404 (0.39-0.42)	0.399 (0.39-0.46)	<0.001
Admitted to SCN and/or NICU	1775 (6.2%)	5870 (5.9%)	0.957 (0.91-1.01)	1.027 (0.97-1.09)	0.363
Transferred	232 (0.8%)	4375 (4.4%)	5.661 (4.96-6.47)	6.516 (5.70-7.45)	<0.001
Total Perinatal Mortality	17 (0.59/1000)	76 (0.77/1000)	1.294 (0.77-2.19)	1.294 (0.75-2.23)	0.355

*Any resuscitation includes: Suction, oxygen, IPPR by bag and mask, Intubation and IPPR, external cardiac massage and ventilation and other

** Private hospital is the reference category

For peer review only

Table 5. Morbidity associated with birth admission coded on neonatal birth admission record

ICD-10-AM Grouping		Private n=58300		Public n=179003		
**Certain conditions originating in the perinatal period (P00-P96)**		Count	%	Count	%	p
	Fetus and newborn affected by maternal infectious and parasitic diseases (P00.2)	41	0.07%	989	0.55%	<0.001
	Fetus and newborn affected by forceps delivery (P03.2)	473	0.81%	1108	0.62%	<0.001
	Fetus and newborn affected by delivery by vacuum extractor (P03.3)	511	0.88%	1509	0.84%	0.46
	**Birth trauma (all body systems) (P10-P15)**	**2948**	5.06%	**6447**	3.60%	<0.001
	- specifically to scalp (included in above total) (P12)	1880	3.22%	3965	2.22%	<0.001
	Intrauterine hypoxia (P20)	993	1.70%	2170	1.21%	<0.001
	Other specified respiratory conditions of newborn (P28)	562	0.96%	1015	0.57%	<0.001
	Benign and innocent cardiac murmurs in newborn (P29.82)	139	0.24%	303	0.17%	=0.001
	Neonatal conjunctivitis specific to the perinatal period (P39.1)	1740	2.98%	2267	1.27%	=0.001
	**Jaundice related conditions (P58-P59)**	**2728**	4.68%	**5166**	2.89%	<0.001

For peer review only - http://bmjopen.bmj.com/site/about/guidelines.xhtml

	Transitory disorders of carbohydrate metabolism specific to fetus and newborn (P70)	502	0.86%	942	0.53%	<0.001
	Conditions involving the integument & temperature regulation of fetus and newborn (P80-P83)	1214	2.08%	2275	1.27%	<0.001
	Vomiting in newborn (P92.0)	320	0.55%	693	0.39%	<0.001
	Feeding problems in newborn (P92)	2231	3.83%	4157	2.32%	<0.001
**factors influencing health status and contact with health services (Z00-Z99)**						
	Routine and ritual circumcision (Z41.2)*	1552	5.31%	187	0.21%	<0.001
	Observation and evaluation of newborn (Z03)	2187	3.75%	11372	6.35%	<0.001
	Prophylactic chemotherapy (antibiotics) (Z29.2)	93	0.16%	935	0.52%	<0.001
	Socioeconomic circumstances (housing, distance, adoption, assumption of care) (Z76)	32	0.05%	1020	0.57%	<0.001

* as a % of male babies

For peer review only - http://bmjopen.bmj.com/site/about/guidelines.xhtml

Table 6. Morbidity associated with readmission of the baby <28 days of age

ICD-10-AM Grouping		Private n=58300 Count	%	Public n=179003 Count	%	p
**Certain infectious and parasitic diseases (A00-B99)**		**121**	0.21%	**217**	0.12%	<0.001
**Endocrine nutritional and metabolic diseases (E00-E89)**		**30**	0.05%	**38**	0.02%	<0.001
	Volume depletion (E86)	18	0.03%	12	0.01%	<0.001
**Mental and behavioural disorders (F00-F99)**		**33**	0.06%	**30**	0.02%	<0.001
	Non-organic hypersomnia (F51.1)	17	0.03%	19	0.01%	=0.002
**Diseases of the nervous system (G00-G99)**		**34**	0.06%	**38**	0.02%	<0.001
	Disorders of the sleep wake schedule (G47.2)	18	0.03%	9	0.01%	<0.001
**Diseases of the ear and mastoid process (H60-H95)**		**19**	0.03%	**23**	0.01%	=0.002
**Diseases of the respiratory system (J00-J99)**		**155**	0.27%	**351**	0.20%	=0.002
	Acute obstructive laryngitis (croup) (J05)	6	0.01%	16	0.01%	=0.96
	Acute upper respiratory infection unspecified (J06)	16	0.03%	52	0.03%	=0.96

For peer review only - http://bmjopen.bmj.com/site/about/guidelines.xhtml

	Pneumonia (J10-J18)	6	0.01%	19	0.01%	=0.96
	Acute bronchiolitis (J21)	70	0.12%	175	0.10%	=0.17
	Unspecified acute lower respiratory tract infection (J22)	3	0.01%	12	0.01%	*
**Diseases of the digestive system (K00-K93)**		**53**	0.09%	**92**	0.05%	=0.001
	Gastro-oesophageal reflux disease (K21)	26	0.04%	37	0.02%	=0.003
**Certain conditions originating in the perinatal period (P00-P96)**		**474**	0.81%	**1011**	0.56%	<0.001
	Cephalohaematoma due to birth trauma (P12.0)	14	0.02%	13	0.01%	=0.002
	Total birth trauma to scalp (P12)	30	0.05%	32	0.02%	<0.001
	Intrauterine hypoxia (P20)	4	0.01%	25	0.01%	*
	Other neonatal hypoglycaemia (P70.4)	14	0.02%	14	0.01%	=0.002
	Other transitory neonatal electrolyte and metabolic disturbances (P70.8)	5	0.01%	21	0.01%	=0.527
	Fever of newborn (P81.9)	5	0.01%	18	0.01%	0.753
	Feeding problems of newborn (P92)	40	0.07%	100	0.06%	=0.271
	**Neonatal jaundice (P58)**	**193**	0.33%	**90**	0.05%	<0.001
**Symptoms, signs and abnormal findings not elsewhere classified (R00-R99)**		**198**	0.34%	**340**	0.19%	<0.001
	Fever (R50)	19	0.03%	27	0.02%	=0.008

For peer review only - http://bmjopen.bmj.com/site/about/guidelines.xhtml

	Feeding difficulties and mismanagement (R63.3)	25	0.04%	37	0.02%	=0.003
	Excessive crying (R68.1)	83	0.14%	117	0.07%	<0.001
**Injury, poisoning and certain other consequences of external causes (S00-T98)**		**15**	0.03%	**94**	0.05%	=0.009
	Burns (T20-T31)	0	0.00%	30	0.02%	*
**Factors influencing health status and contact with health services (Z00-Z99)**		**192**	0.33%	**691**	0.39%	=0.051
	Observation and evaluation in newborn (Z03)	34	0.06%	102	0.06%	=0.907
	Prophylactic chemotherapy (antibiotics) (Z29.2)	5	0.01%	49	0.03%	=0.009
	Routine and ritual circumcision (Z41.2)	68	0.20%	119	0.13%	<0.001
	Attention to surgical dressings and sutures (Z48.0)	0	0.00%	28	0.02%	*
	Socio-economic circumstances (housing, distance, adoption, assumption of care) (Z76)	22	0.04%	156	0.09%	<0.001

***cell size too small to calculate chi-square ** as a % of male babies**

For peer review only - http://bmjopen.bmj.com/site/about/guidelines.xhtml

Table 7. Combined birth and readmission neonatal morbidity for selected codes

	Private	Public	P*
Total feeding difficulties	2314 (4.0%)	4306 (2.4%)	<0.0001
Total circumcision**	1620 (5.6%)	306 (0.3%)	<0.0001
Total socio-economic circumstances	54 (0.1%)	1176 (0.7%)	<0.0001
Total birth trauma	2922 (5.0%)	6492 (3.6%)	<0.0001
Total hypoxia	997 (1.7%)	2195 (1.2%)	<0.0001
Total jaundice	2818 (4.8%)	5359 (3.0%)	<0.0001
Total respiratory	717 (1.2%)	1366 (0.8%)	<0.0001
Total sleep/behavioural issues	118 (0.2%)	145 (0.1%)	<0.0001
Prophylactic antibiotics	98 (0.2%)	982 (0.6%)	<0.001

*chi-square ** as a % of male babies

For peer review only

References

1 Li Z, Zeki R, Hilder L, Sullivan EA. Australia's mothers and babies 2011. Perinatal statistics series no. 28. Cat. no. PER 59. In: Unit ANPEaS, editor. Canberra2013.

2 Robson SJ, Laws P, Sullivan EA. Adverse outcomes of labour in public and private hospitals in Australia: a population based descriptive study. The Medical Journal of Australia 2009;190(9):474-7.

3 Tracy S, Welsh A, Dahlen H, Tracy M. Letter to the Editor re Robson SJ, Laws P, Sullivan EA. Adverse outcomes of labour in public and private hospitals in Australia: a population-based descriptive study. Med J Aust 2009; 190: 474-477. Medical Journal of Australia. 2009;191(10):579-80.

4 Clark E.A.S, Silver RM. Long-term maternal morbidity associated with repeat caesarean delivery. American Journal of Obstetrics and Gynecology. 2011;S2(December).

5 Hyde MJ, Mostyn A, Modi N, Kemp PR. The health implications of birth by caesarean section. Biological Reviews. 2012;87(1):229-43.

6 Tracy S, Tracy M, Sullivan E. Admission of Term Infants to Neonatal Intensive Care: A Population-Based Study. Birth 2007;34(4):301-7.

7 Tracy S, K , Tracy M. Costing the cascade: estimating the cost of increased obstetric intervention in childbirth using population data. British Journal of Obstetrics and Gynaecology. 2003;110:717-24.

8 Allen VM, O'Connell CM, Farrell SA, Baskett TF. Economic implications of method of delivery. Am J Obstet Gynecol 2005;193(1):192-7.

9 Dahlen HG, Tracy S, Tracy M, Bisits A, Brown C, Thornton C. Rates of obstetric intervention among low-risk women giving birth in private and public hospitals in NSW: a population-based descriptive study. BMJ Open. 2012;2:e001723 doi:10.1136/bmjopen-2012-001723.

10 Roberts C, L,, Tracy S, Peat B. Rates of obstetric intervention among private and public patients in Australia: population based descriptive study. British Medical Journal. 2000;312:137-41.

11 Buist R. Letter to the Editor: Private Obstetrics-again. BMJ Open. 2012;2(5):e001723 doi:10.1136/bmjopen-2012-.

12 CHeReL. Centre for Health Record Linkage (CHeReL). Quality Assurance Report 2012. http://wwwcherelorgau/media/24160/qa_report_2012pdf. 2012.

For peer review only

13 Lain SJ, Hadfield RM, Raynes-Greenow CH, Ford JB, Mealing NM, Algert CS, et al. Quality of data in perinatal population health databases: a systematic review. Med Care. 2012;50(4):e7-e20.

14 Roberts CL, Tracy S, Peat B. Rates for obstetric intervention among private and public patients in Australia: population based descriptive study. British Medical Journal. 2000 2000;321(7254):137-41.

15 Einarsdóttir K, Stock S, Haggar F, Hammond G, Langridge AT, Preen DB, et al. Neonatal complications in public and private patients: a retrospective cohort study. BMJ Open. 2013;Downloaded from bmjopen.bmj.com on November 11, 2013.

16 Evans N, Malcolm G, Gordon A. Letter to the Editor re Adverse outcomes of labour in public and private hospitals in Australia. MJA. 2009;191(10):579.

17 Chambers D. Letter to the Editor re Adverse outcomes of labour in public and private hospitals in Australia. MJA. 2009;191(10):578-9.

18 Wyllie J. Recent changes to UK newborn resuscitation guidelines. Arch Dis Child Fetal Neonatal Ed. 2012;97(1):F4-F7.

19 Kannapiran R, MKennea N. Resuscitation of the newborn. Obstetrics, Gynecology & Reproductive Medicine. 2012;22(4):92-7.

20 Doumouchtsis SK, Arulkumaran S. Head injuries after instrumental vaginal deliveries. Current Opion in Obstetrics and Gynaecology. 2006;18:129-34.

21 Towner D, Castro MA, Wilkens EE, Gilbert WM. Effect of Mode of Delivery in Nulliparous Women on Neonatal Intracranial Injury. The New England Journal of Medicine 1999;341:1709-14.

22 Linder N, Linder I, Fridman E, Kouadio F, Lubin D, Merlob P, et al. Birth trauma – risk factors and short-term neonatal outcome. The journal of Maternal-fetal & Neonatal Medicine. 2013;DOI: 10.3109/14767058.2013.789850.

23 Bertini G, Dani C, Tronchin M, Rubaltelli FF. Is breastfeeding really favouring early neonatal jaundice? Pediatrics. 2006;107(3):e41.

24 Bhutani VK, Stark AR, Lazzeroni LC, Poland R, Gourley GR, Kazmierczak S, et al. Predischarge Screening for Severe Neonatal Hyperbilirubinemia Identifies Infants Who Need Phototherapy. The Journal of Pediatrics. 2013;162(3):477-82.

25 Young PC, Korgenski K, Buchi KF. Early Readmission of Newborns in a Large Health Care System. Pediatrics. 2013;131(5):e1538-e44.

26 Brown A, Jordan S. Impact of birth complications on breastfeeding duration: an internet survey. Journal of Advanced Nursing. 2012;69(4):828-39.

27 Chapman D.J, Perez-Escamilla R. Identification of risk factors for delayed onset oflactation. Journal of the American Dietetic Association. 2003;99:450-4.

For peer review only

28 Dewey K.G., Nommsen-Rivers L.A., Heinig M.J, Cohen R.J. Risk factors for suboptimal infant breastfeeding behavior, delayed onset of lactation and excess neonatal weight loss. Pediatrics 2003;112(3):607-19.

29 Scott J.A., Binns C.W, Oddy W.H. Predictors of delayed onset of lactation. Maternal and Child Nutrition. 2007;3(3):186-93.

30 Jordan S., Emery S., Watkins A., Evans J.D., Storey M, Morgan G. Associations of drugs routinely given in labour with breastfeeding at 48 hours: analysis of the Cardiff Births Survey. British Journal of Gynecology. 2009;116(12):1622-9.

31 Leung G.M, Lam T.H, Ho L.M. Breast-feeding and its relation to smoking and mode of delivery. Obstetrics and Gynecology 2002;99(5):785-94.

32 Jordan S. Infant feeding and analgesia in labour: the evidence is accumulating. International Breastfeeding Journal Research. 2006;1(25):doi: 10.1186/746-4358-1-25.

33 Henderson J.J., Dickinson J.E., Evans S.F., McDonald S.J, M.J. P. Impact of intrapartum epidural analgesia on breast- feeding duration. Australian and New Zealand Journal of Obstetrics and Gynaecology. 2003;43(5):372–7.

34 Beilin Y., Bodian C., Weiser J., Sabera H., Ittamar A., Feierman D., et al. Effect of labor analgesia with and without fentanyl on infant breastfeeding: a prospective, randomized double-blind study. Anesthesiology. 2005;103(6):1211– 7.

35 Ohnsorg T, Schiff J. Preventing elective induction before 39 weeks. , 93(11), 44-46. Minnesota Medicine. 2010;93(11):44-6.

36 Oshiro BT, Henry E, Wilson J, Branch DW, Varner MW. Decreasing elective deliveries before 39 weeks of gestation in an integrated health care system. Obstetrics & Gynecology. 2009;113:804-11.

37 Reddy UM, Ko CW, Willinger M. “Early term” births (37-38 weeks) are associated with increased mortality. American Journal of Obstetrics & Gynecology. 2006;195(S202).

38 McLaughlin FJ, O’Connor S, Deni R. Infant state and behavior during the first postpartum hour. The Psychological Record 1981;31:455–8.

39 Widstrom A-M, Ransjo-Arvidson AB, Christensson K, Matthiesen A- S, Winberg J, Uvnas-Moberg K. Gastric suction in healthy newborn infants. Effects on circulation and developing feeding behaviour. Acta Paediatr. 1987;76:566–72.

40 Righard L. How do newborns find their mother’s breast? . Birth. 1995;22:174–5.

41 Fisher J, Astbury J, Smith A. Adverse psychological impact of opera- tive obstetric interventions: a prospective study. Aust NZ J Psychiatry 1997;31:728–38.

For peer review only

42 Adams-Chapman I. Insults to the developing brain and impact on neurodevelopmental outcome. Journal of Communication Disorders. Journal of Communication Disorders. 2009;42:256-62

43. Gülmezoglu AM, Crowther CA, Middleton P, Heatley E. Induction of labour for improving birth outcomes for women at or beyond term. *Cochrane Database of Systematic Reviews* 2012;Issue 6. Art.No.:CD004945. DOI: 10.1002/14651858.CD004945.pub3.

44. The Royal Australian College of Physicians. Circumcision of infant males. In: Physicians TRACo, editor. Sydney: The Royal Australasian College of Physicians; 2010.

45. Bentley JP, Ford JB, Taylor LK, Irvine KA, Roberts CL. Investigating linkage rates among probabilistic linked births and hospital records. BMC Medical Research Methodology. 12:149, 2012.

46. Taylor L, Travis S, Pym M, Olive E, Henderson-Smart D. How useful are hospital morbidity data for monitoring conditions occurring in the perinatal period? Australian & New Zealand Journal of Obstetrics & Gynaecology. 2005;45:36 –41.

47 Roberts C, Bell J, Ford J, Morris J. Monitoring the quality of maternity care: how well are labour and delivery events reported in population health data? . Paediatirc and Perinatal Epidemiology. 2008;23:144-52.

48 Thornton C, Makris A, Ogle R, Hennessy A. Generic obstetric database systems are unreliable for reporting the Hypertensive Disorders of Pregnancy. Australian and New Zealand Journal Obstetrics and Gynaecology. 2004;44(505-509).

49 Finkelstein A, Fiebelkorn IC, Wang G. National medical Spending attributable to overweight and obesity: How much and who's paying. Health Affairs, 2003 Project HOPE–The People-to-People Health Foundation, Inc., 219-226. 2003.

For peer review only

Figure 1. Comparison of gestation at delivery between all low risk women stratified by hospital type

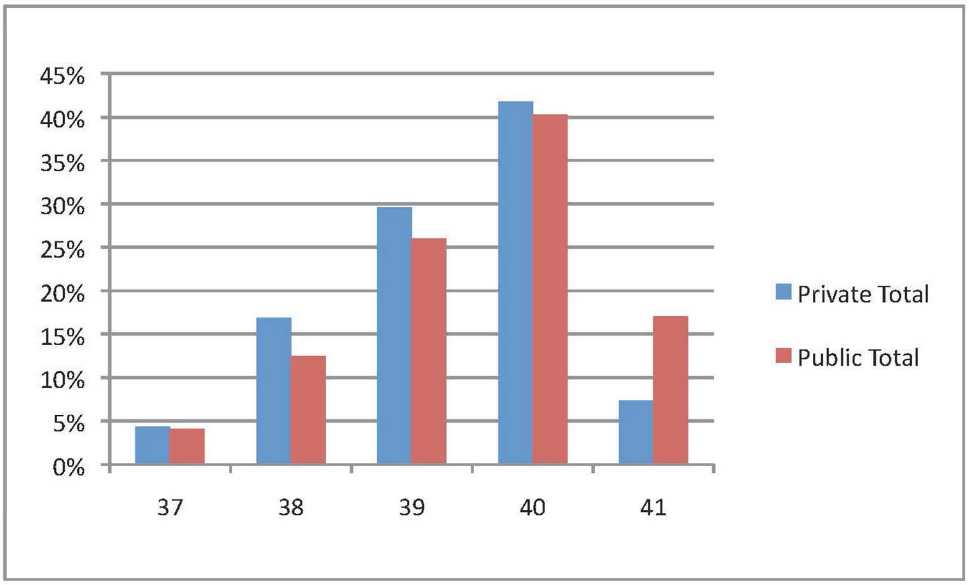

118x91mm (300 x 300 DPI)

ew only

Figure 2. Birth trauma as a percentage of all births in Private and Public Hospitals

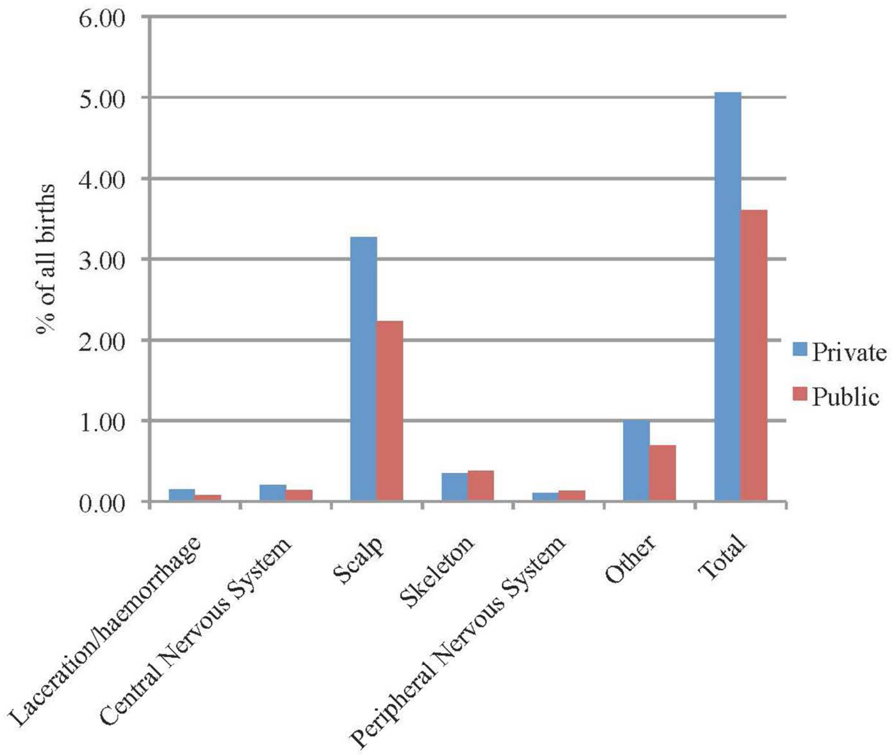

116x100mm (300 x 300 DPI)

For peer review only
